# A unicellular relative links aggregative multicellularity to animal origins

**DOI:** 10.1038/s41586-026-10748-5

**Published:** 2026-06-09

**Authors:** Ruibao Li, Jennah E. Dharamshi, Kyle Kwok, Iñaki Ruiz-Trillo, Joseph P. Gerdt

**Affiliations:** 1 Department of Chemistry, Indiana University Bloomington, Bloomington, IN USA; 2 Department of Organismal Biology, Systematic Biology, Uppsala University, Uppsala, Sweden; 3 Institut de Biologia Evolutiva (CSIC-Universitat Pompeu Fabra), Barcelona, Spain; 4 ICREA, Barcelona, Spain

**Keywords:** Evolutionary developmental biology, Evolutionary genetics, Cell adhesion, Nutrient signalling, Symbiosis

## Abstract

How animals evolved complex multicellularity from their unicellular ancestors remains unanswered. Unicellular relatives of animals exhibit simple multicellularity through clonal division, formation of multinucleate coenocytes or aggregation^1^. Animal multicellularity may therefore have evolved from one (or a combination) of these behaviours. Aggregation has classically been dismissed as a means to complex multicellularity^2^. However, aggregation occurs in many extant animal cells and has also been recently described in three close unicellular relatives of animals (the choanoflagellates *Salpingoeca rosetta* and *Choanoeca flexa*, and the filasterean *Capsaspora owczarzaki*)^3–5^. It is unclear whether aggregation in these species is derived or ancestral, and its relevance for animal origins remains unclear. Here, to fill this gap, we investigated whether an additional close unicellular relative of animals can undergo aggregation. We found that the marine free-living bacterivorous filasterean *Ministeria vibrans*^6^ forms homogeneous aggregates with reproducible kinetics that have long-term stability, and that improved feeding and mating may be evolutionary drivers of this aggregation. Notably, we found that homologues of many animal multicellularity genes involved in cell adhesion, signalling and transcriptional regulation were deployed during the aggregation process, indicating that they may have been used for aggregation in the unicellular ancestors of animals before being co-opted into animal multicellular development. Thus, our results are consistent with aggregative multicellularity being key to the evolution of the multicellular animal genetic toolkit.

## Abstract (web summary)

Aggregative multicellularity was key to the evolution of the multicellular animal genetic toolkit.

## Main

**Fig. 1:**
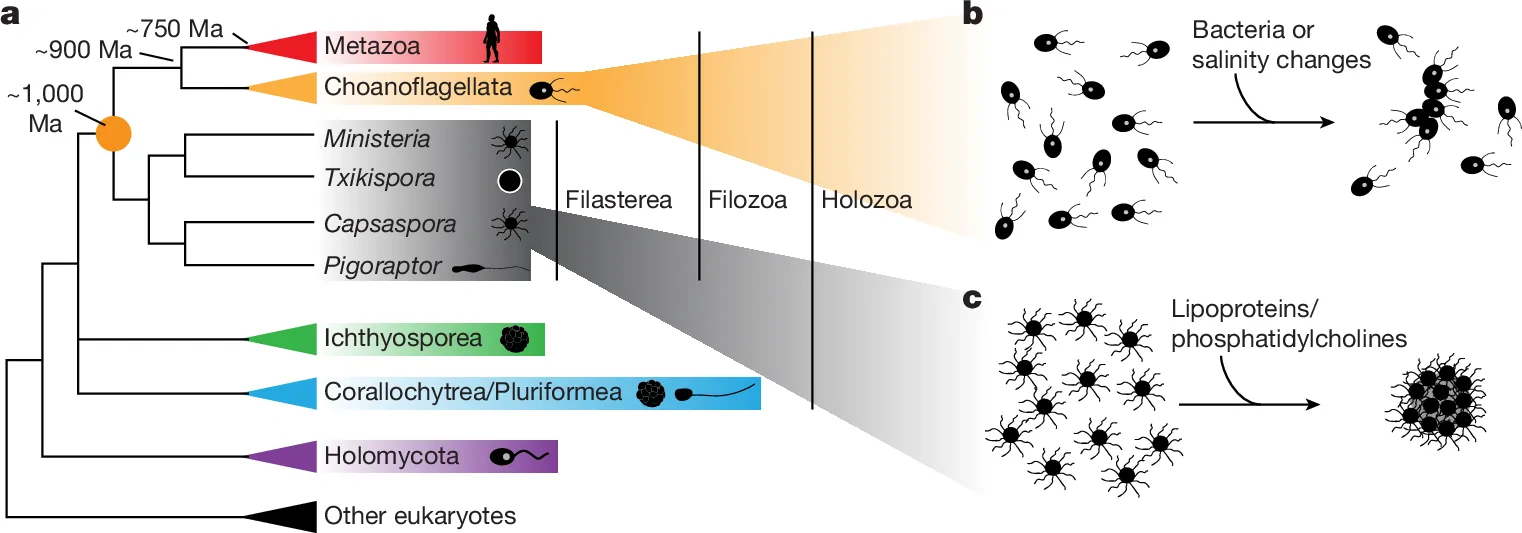
Multicellular aggregation in unicellular holozoans. **a**, Schematic phylogeny of holozoans. The orange circle represents the ancestor of Filozoa—the clade that comprises metazoans, choanoflagellates and filastereans. Approximate ages of the last common ancestors of Filozoa, Choanozoa and Metazoa (in million years ago, Ma) are displayed^7^. **b**, The choanoflagellate *S. rosetta* forms moderately sized, stringy, multicellular aggregates in the presence of bacterial chondroitinases^3^. A fraction of *C. flexa* choanoflagellate cells also aggregate after changes in salinity^4^. **c**, The filasterean *C. owczarzaki* homogeneously forms large multicellular aggregates in the presence of lipoproteins and phosphatidylcholine lipids from animal hosts^5,13^. The diagrams in **a**–**c** were created using BioRender; Gerdt, J. P. https://BioRender.com/bitcuve (2026).

How animals (Metazoa) evolved from their unicellular ancestors remains a mystery. One of the most contentious debates about the transition is how the last unicellular ancestor of animals looked and behaved. Data from diverse unicellular animal relatives (that is, choanoflagellates, filastereans, ichthyosporeans and corallochytreans, which together with Metazoa form the Holozoa clade) suggest that the last unicellular animal ancestor had the capacity for simple regulated multicellularity (see Fig. 1a for holozoan phylogeny with estimated ages of the last common ancestors of constituent clades^7^). Namely, several choanoflagellate species can form multicellular colonies through clonal division, the filasterean *C. owczarzaki* can form temporary multicellular aggregates, and most ichthyosporeans and corallochytreans can form multinucleated coenocytes^4,5,8–10^.

Aggregation has been largely neglected as a foundation for evolving multicellularity in the first animal. Evolving complex multicellularity through aggregation has often been excluded due to the theoretical costs of preventing cheating^11^. However, finding multicellularity through regulated cell aggregation in close relatives of animals^4,5^, together with research showing that spatiality combined with group selection facilitates the survival of cooperators against cheaters in an aggregate, challenged those views^12^. However, to date, our knowledge on aggregation in unicellular animal relatives is limited and primarily based on the filasterean *C. owczarzaki* and two case studies in choanoflagellates^3–5^. As *C. owczarzaki* aggregates in response to lipid cues from its putative animal host^13^, this behaviour may have been a relatively recent adaptation to its lifestyle as a symbiont. Two choanoflagellates have been reported to form moderate-sized aggregates due to bacteria and salinity^3,4^. However, these recently identified behaviours have not yet been studied extensively (Fig. 1b,c). Furthermore, gene expression has not been studied dynamically throughout the process of aggregation in any of these three organisms—possibly due to the technical challenges of consistently inducing aggregation with reproducible kinetics. It is therefore still unclear whether aggregation was ancestral in unicellular animal relatives and whether genes critical for animal multicellularity and development are deployed during their aggregative behaviours. If they are, it would suggest that the unicellular ancestor of animals had the capacity for multicellular aggregation.

To address this question, here we studied the filasterean relative of *C. owczarzaki*,* M. vibrans*^6,14^ (Fig. 1a). It is a marine free-living bacteriovore—the lifestyle that is typically ascribed to the last unicellular ancestor of animals^15^. However, no notable *M. vibrans* aggregates have been reported beyond tiny clumps of around 5 cells^6,16^. Finding aggregation in a second filasterean (which is free-living) would suggest that aggregation is ancestral in Filozoa (Fig. 1a). We found that *M. vibrans* formed large aggregates homogeneously in monoxenic culture with the bacterium *Thalassospira lucentensis* while upregulating many genes associated with animal multicellular cell adhesion, signalling and transcriptional regulation. We also found that aggregation appeared to improve feeding and plausibly mating. Overall, these findings suggest that the last unicellular ancestor of animals had the capacity to form multicellular aggregates, using many genes that were later crucial for multicellularity and development in extant animals.

## Bacteria induce *M. vibrans* clusters

**Extended Data Fig. 1:**
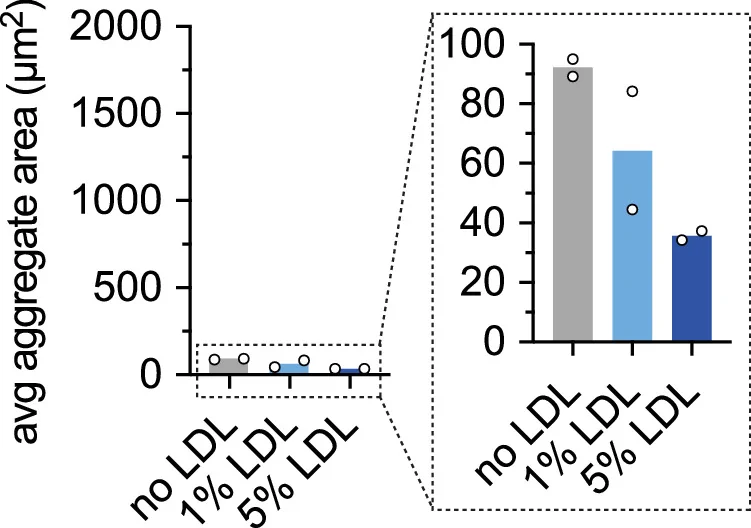
LDL does not induce aggregation in *M. vibrans.* Average (avg) aggregate area quantified for the xenic *M. vibrans* culture in the absence and presence of low density lipoproteins (LDLs, which induce aggregation in *C. owczarzaki*). Error bars represent the range of a biological duplicate. Individual replicates are displayed with circles.

To use *M. vibrans* as a model for the involvement of cellular aggregation in a free-living unicellular holozoan, we first needed to identify a condition in which it reproducibly aggregated. Consistent with other reports of only occasional tiny clusters of a few cells^16^, we too found no significant aggregates in the *M. vibrans* culture obtained from the American Type Culture Collection (strain Tong). Given the previous finding that *C. owczarzaki* aggregation is induced by lipoprotein complexes^5,13^, we applied low-density lipoproteins to the *M. vibrans* culture. However, this treatment did not induce aggregation (Extended Data Fig. 1).

**Fig. 2:**
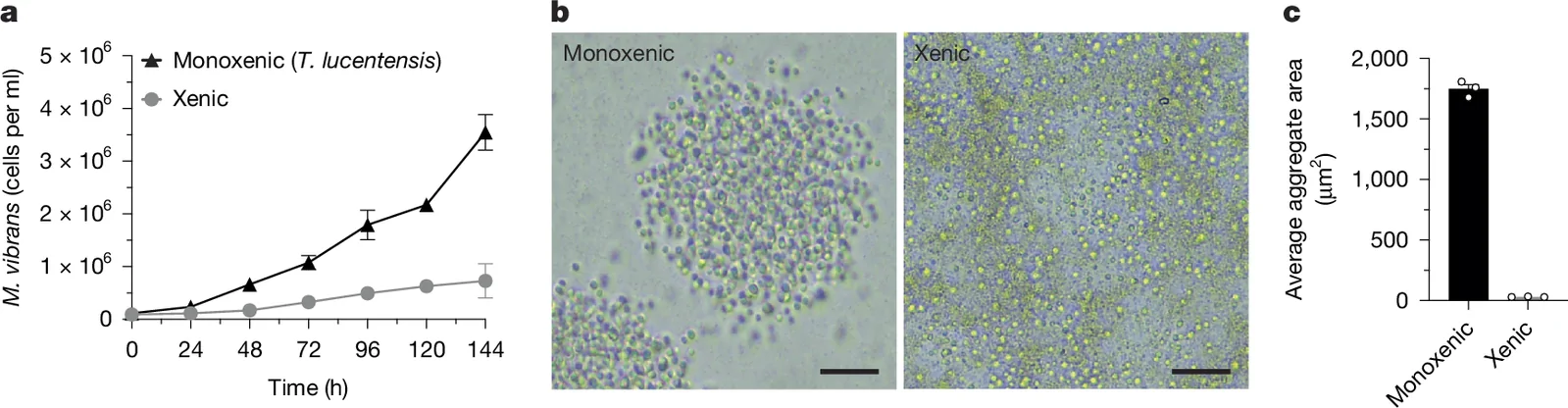
*M. vibrans* forms multicellular clusters in monoxenic culture with an alphaproteobacterium. **a**, Growth curves of the monoxenic *M. vibrans* culture with only *T. lucentensis* bacteria compared with the original xenic culture. Data are mean ± s.e.m. of a biological triplicate. **b**, Representative images of aggregates formed in the monoxenic culture compared to the original xenic culture. Images are representative of three biological replicates. Scale bars, 20 μm.** c**, Average aggregate area quantified for the monoxenic *M. vibrans* culture compared with the original xenic culture. Data are mean ± s.e.m. of a biological triplicate. Individual replicates are displayed with circles.

**Extended Data Fig. 2:**
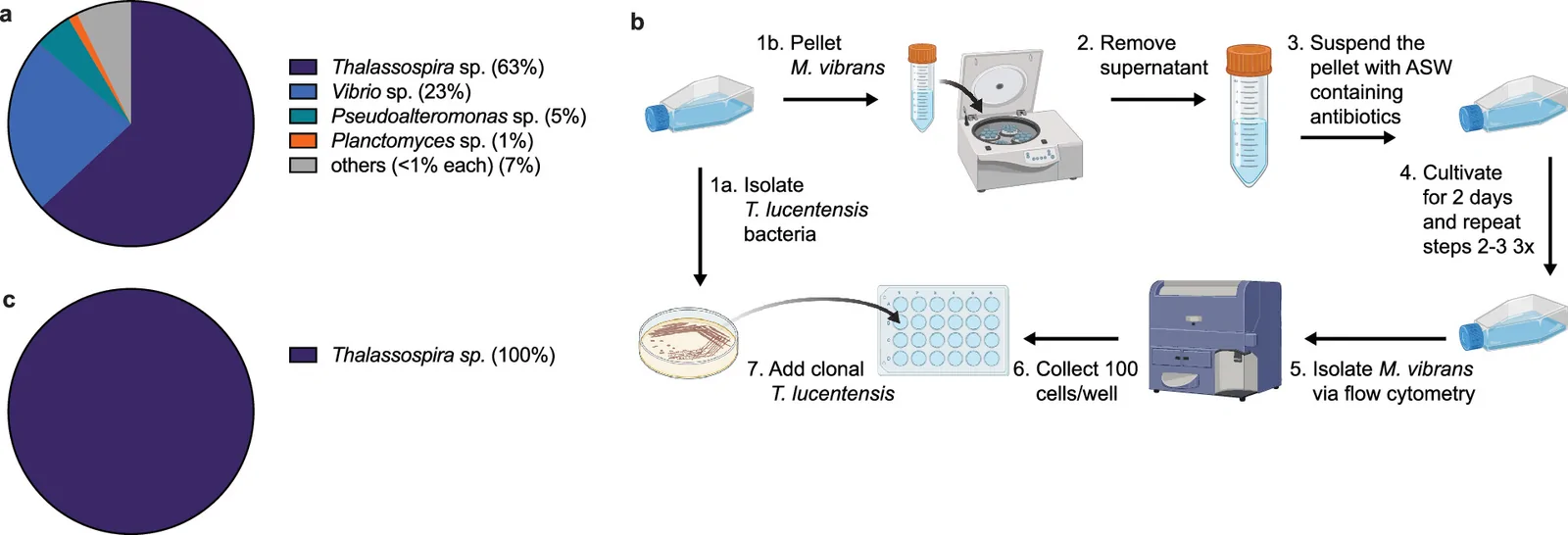
Generating a monoxenic *M. vibrans* culture. a) Results of 16S rDNA amplicon sequencing of the original xenic *M. vibrans* culture (Data S1). The four bacterial genera present at >1% of the population are indicated. b) Procedure to generate a monoxenic culture of *M. vibrans* with *T. lucentensis* Mv1 (Data S2). ASW is “artificial sea water”. c) Result of 16S rDNA amplicon sequencing of the monoxenic *M. vibrans* culture. All reads were assigned to *Thalassospira*, originating from *T. lucentensis* Mv1. (Data S3). The diagram was created in part using BioRender; Gerdt, J. P. https://BioRender.com/84jcu4o (2026).

As low-density lipoproteins did not induce *M. vibrans* aggregation, we next considered that *M. vibrans* may aggregate in response to the presence of specific bacteria, as observed for the choanoflagellate *S. rosetta*^3^. We first assessed which bacteria were present in the original *M. vibrans* xenic culture using 16S rDNA amplicon sequencing and found 45 unique bacteria, with *Thalassospira* being the most abundant (63%; Extended Data Fig. 2 and Supplementary Data 1). We isolated colonies of a single *Thalassospira* bacterium (*T. lucentensis* Mv1; Supplementary Data 2) from the *M. vibrans* culture and reintroduced it into flow-cytometry-purified *M. vibrans* to generate a monoxenic culture (Extended Data Fig. 2). The monoxenic culture was verified by 16S rDNA amplicon sequencing, which revealed no other bacteria present (Extended Data Fig. 2 and Supplementary Data 3). The monoxenic culture grew well—in fact better than the original xenic culture (Fig. 2a). Notably, in the monoxenic culture, *M. vibrans* formed large multicellular clusters (Fig. 2b,c) that resembled those formed by *C. owczarzaki*^5,13^. Thus, monoxenic cultivation with the alphaproteobacterium *T. lucentensis* triggers multicellular behaviour in *M. vibrans*.

## *M. vibrans* aggregates reproducibly

**Fig. 3:**
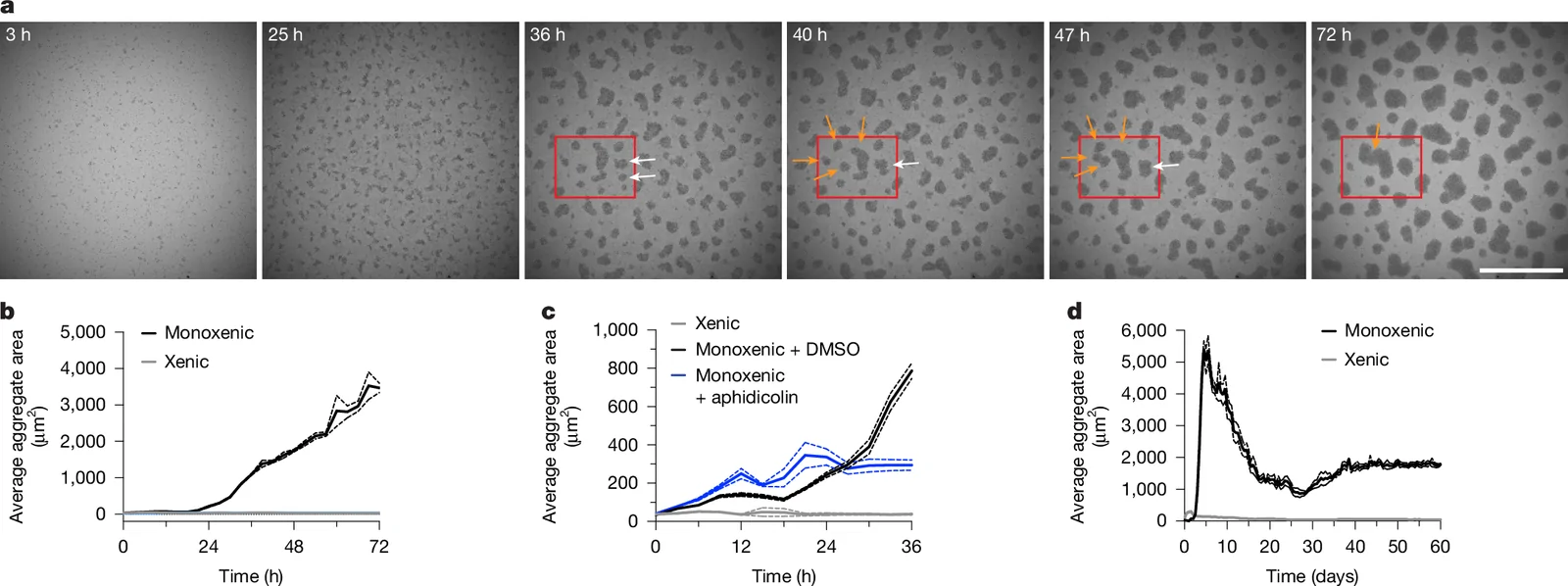
*M. vibrans* forms homogeneous multicellular clusters through aggregation. **a**, Representative images of the formation of multicellular aggregates by the monoxenic *M. vibrans* culture after dilution into fresh growth medium (Supplementary Video 1). The white and orange arrows in the red box highlight two cases of small aggregates merging into large aggregates. Images are representative of four biological replicate cultures (one image per culture per timepoint). Scale bar, 1,000 μm.** b**, The average aggregate area quantified for *M. vibrans* cultures (monoxenic versus xenic control) over time after dilution into fresh growth medium (Supplementary Video 1). Data are mean (solid lines) ± s.e.m. (dashed lines) of a biological triplicate.** c**, The average aggregate area quantified for the *M. vibrans* cultures incubated with aphidicolin (an inhibitor of replication, 20 µg ml^−1^; Extended Data Fig. 3 and Supplementary Video 2). Controls are also shown for non-treated cultures (monoxenic and xenic). Data are mean (solid lines) ± s.e.m. (dashed lines) of a biological quadruplicate.** d**, The average aggregate area quantified for the *M. vibrans* cultures (monoxenic versus xenic) over 60 days (1,440 h) after dilution into fresh growth medium (Supplementary Video 3). Data are mean (solid lines) ± s.e.m. (dashed lines) of a biological quadruplicate.

**Extended Data Fig. 3:**
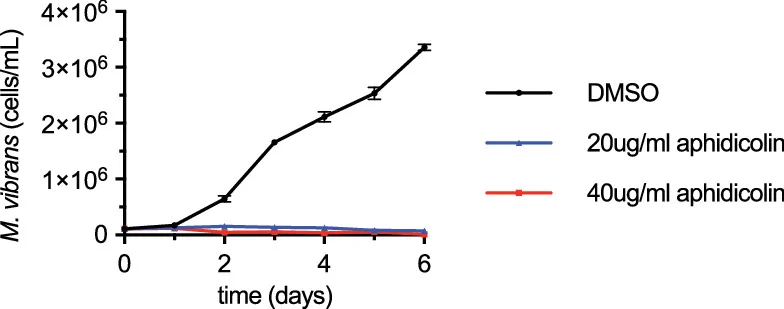
Validation of aphidicolin inhibition of *M. vibrans* replication. Growth curves of the monoxenic *M. vibrans* culture with aphidicolin (an inhibitor of replication) compared to a DMSO control. Scale bars represent standard error of the mean (SE) of a biological triplicate. The 20 µg/ml concentration was used in Fig. 3c to arrest cell division.

To assess the potential of *M. vibrans* for investigating the involvement of multicellular genes in unicellular holozoan cellular aggregation, we needed to confirm that its multicellular clusters were formed through aggregation. We first monitored the process of cluster formation over time, which revealed that small clusters of dozens of cells merged into larger multicellular clusters containing hundreds of cells (Fig. 3a–b and Supplementary Video 1). The observation matched previous observations in *C. owczarzaki*^5,13^, suggesting that the large cell clusters were formed by aggregation. We also noted that the timing of cluster formation appeared too fast to arise from a single cell undergoing multiple rounds of cell division. Clusters containing dozens of cells formed within 24 h, which is only enough time for one or two rounds of cell division for *M. vibrans* (Figs. 2a and 3b). Furthermore, we observed that aggregates could form even when replication was blocked by aphidicolin (Fig. 3c, Extended Data Fig. 3 and Supplementary Video 2), showing that daughter cell formation is not required for cell cluster formation. Together, these experiments demonstrate that *M. vibrans* forms large clusters of hundreds of cells through aggregation.

**Extended Data Fig. 4:**
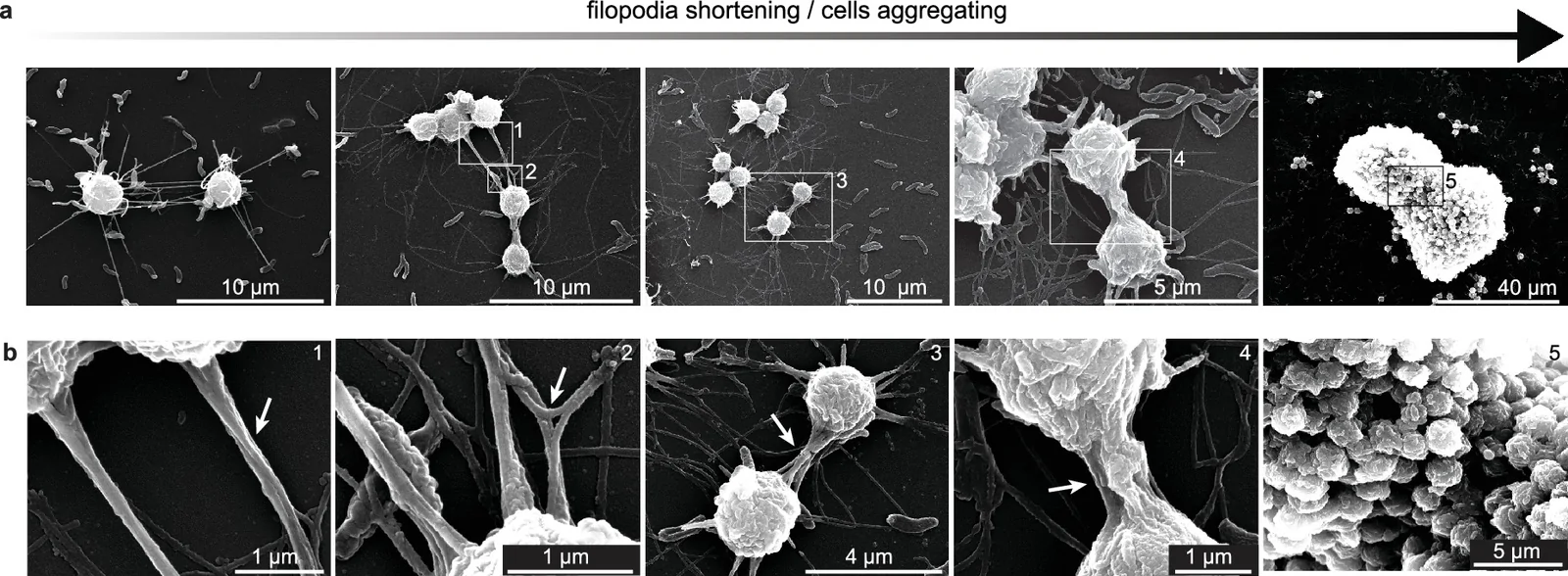
Model for the process of *M. vibrans* aggregation. a) Representative scanning electron micrographs showing the putative process of aggregation from left to right. Filopodia of adjacent cells contact, tangle, and retract to form tight aggregates. Images are representative of 23 images. b) Expansions of the numbered white boxes in panel (a). White arrows highlight entangled filopodia of adjacent cells.

In the related filasterean *C. owczarzaki*, aggregates appear to form through filopodial contacts that contract, pulling the individual cells into tight clusters^5^. Likewise, analysis of scanning electron microscopy (SEM) images of *M. vibrans–T. lucentensis* cultures showed filopodia of different lengths connecting adjacent cells within aggregates (Extended Data Fig. 4). The filopodia appeared to wrap around each other, generating extended surface areas of contact along each filopodium (Extended Data Fig. 4). In many cases, the entangled filopodia appeared to have retracted almost completely (Extended Data Fig. 4), forming very tight interactions between cells—as seen in *C. owczarzaki* aggregates^7^. Notably, *M. vibrans* aggregates in the *T. lucentensis* monoxenic culture persisted for at least 60 days (Fig. 3d and Supplementary Video 3), contrasting with *C. owczarzaki* aggregates, which disperse after 1–3 days—presumably after depletion of the aggregate-inducing lipids^5,13^. This raises the question of whether an unknown inducer of *M. vibrans* aggregation is continuously produced by *T. lucentensis*, resulting in persistent aggregation.

## Dynamics of *M. vibrans* aggregation

For several reasons, the *M. vibrans* aggregative phenotype is ideal for assessing whether cell aggregation could have had a role in animal origins. First, *M. vibrans* is a close unicellular relative of animals, distant from but related to *C. owczarzaki*, which shares many ‘multicellularity’ genes with animals^14^. Second, it has a free-living bacterivorous lifestyle that may match the lifestyle of the last unicellular animal ancestor^15^. Finally, the monoxenic culture homogeneously aggregates with reproducible kinetics and is stable over longer time periods, enabling us to sequence population-level transcriptomes at defined times to assess gene expression of *M. vibrans* as single cells, small initial aggregates and large mature aggregates.

**Fig. 4:**
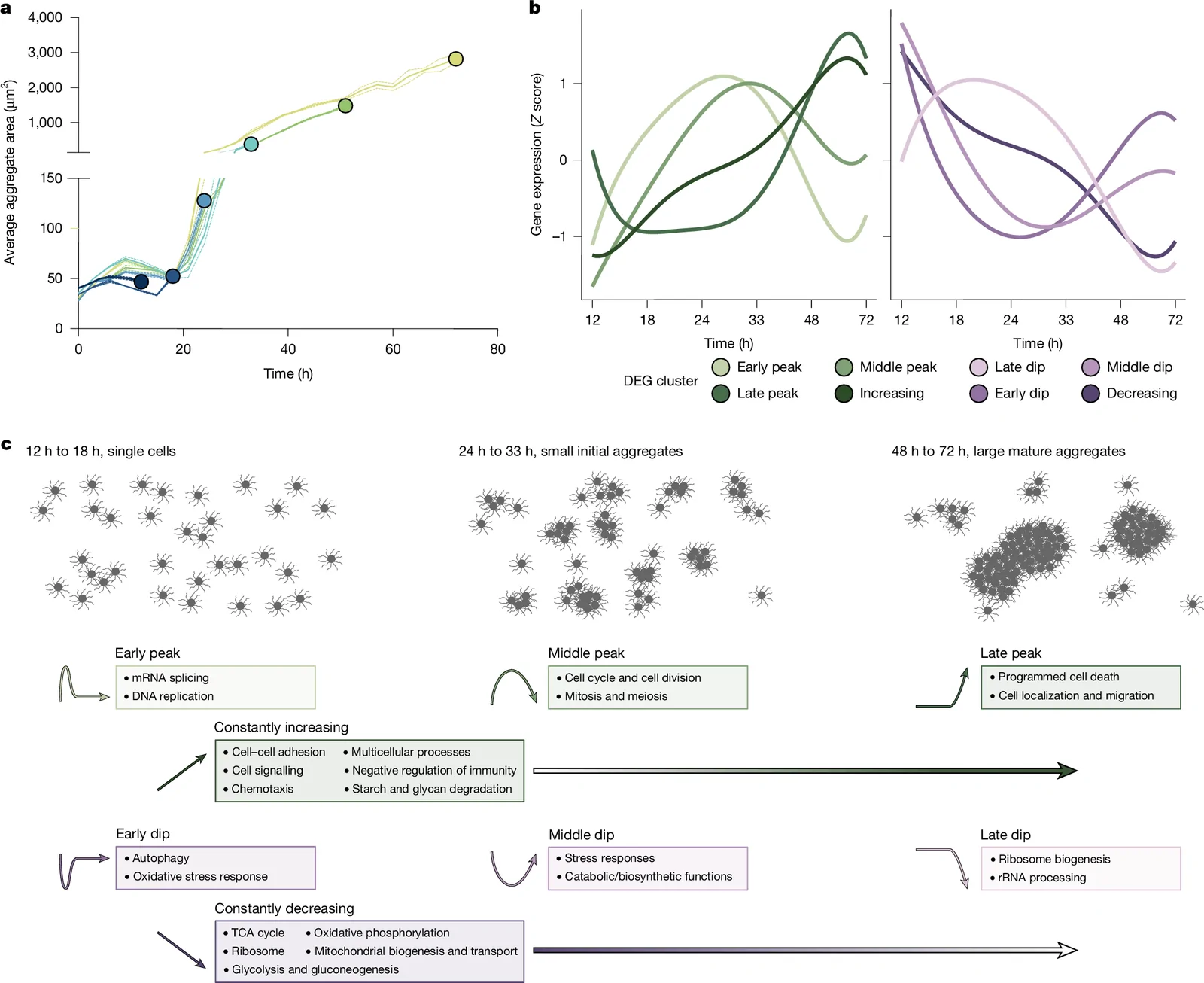
Dynamics of gene expression during *M. vibrans* aggregation time series. **a**, The average aggregate area in four biological replicate *M. vibrans* monoxenic cultures was used to assess gene expression, taken at 12, 18, 24, 33, 48 and 72 h after inoculation into fresh medium. The coloured circle represents the final timepoint at which RNA was extracted for that set of replicates.** b**, The mean gene expression trends for the eight DEG clusters that arose from expression profile clustering analysis. **c**, Schematic of the main groups of genes that increased and decreased in expression across the time series from each DEG cluster based on gene enrichment analyses. See also Extended Data Figs. 5–8, Supplementary Figs. 1 and 2 and Supplementary Data 7 and 8. The constantly increasing and constantly decreasing DEG clusters are also referred to without ‘constantly’. The diagram in **c** was created using BioRender; Gerdt, J. P. https://BioRender.com/bitcuve (2026).

**Extended Data Fig. 5:**
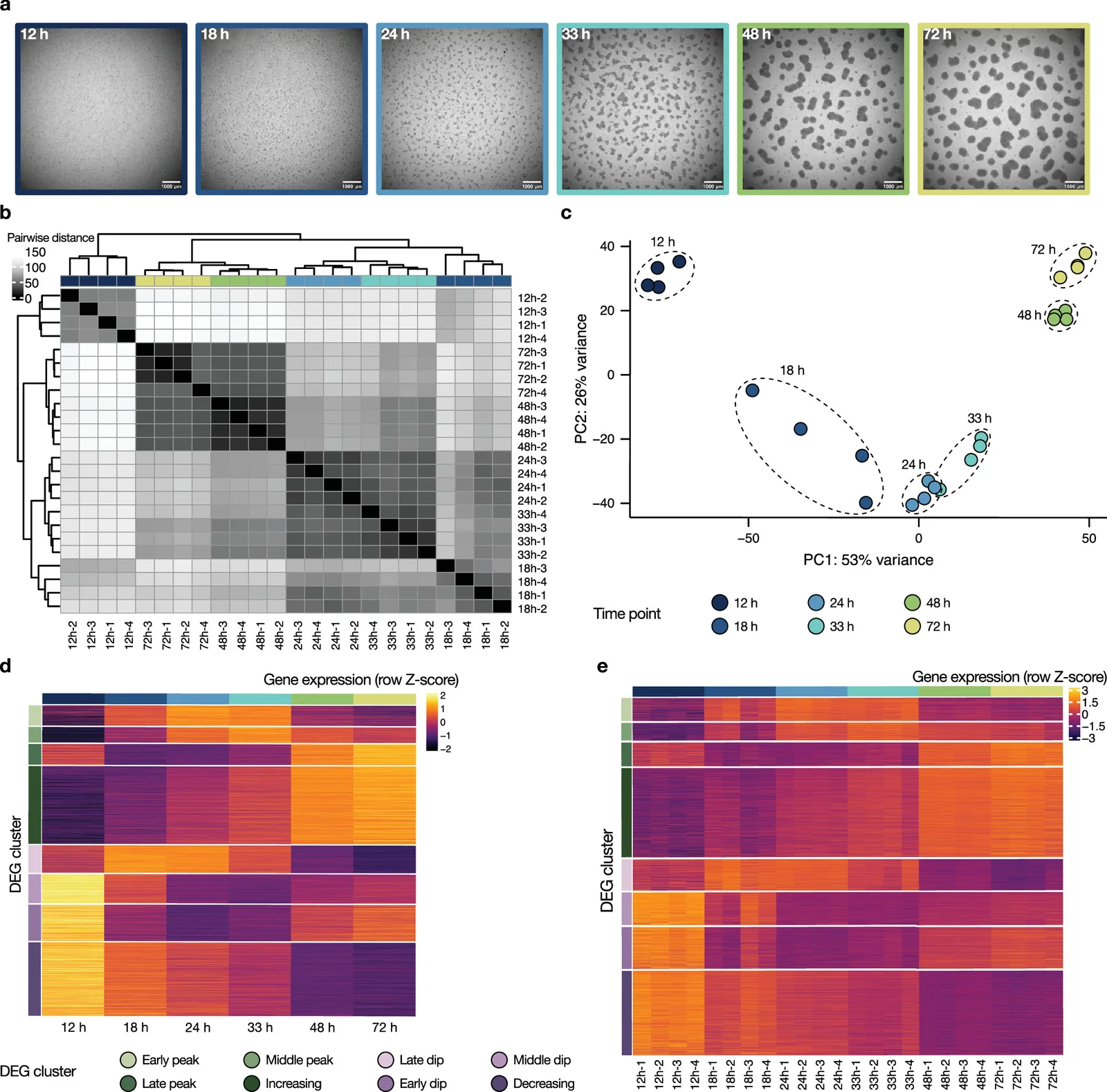
Gene expression during the *M. vibrans* aggregation time series. a) Representative brightfield microscopy images of aggregates across replicates at each time point where RNA was extracted for RNA-sequencing and downstream analysis of gene expression. Images are representative of a biological quadruplicate (one image per replicate well). b) Heatmap of sample-to-sample distances between timepoint replicates using the variance-stabilizing transformation of normalized gene counts. c) PCA plot of gene-expression profiles from replicate cultures taken at different points in the time series showing the variance between replicates and time points. d) Heatmap of gene expression across the time series for genes assigned to each DEG cluster. Mean gene expression values were calculated across replicates from each time point using normalized and variance-stabilizing transformed gene counts that were then centred and row-scaled. e) Heatmap of gene expression across the time series for genes assigned to each DEG cluster. Normalized and variance-stabilizing transformed gene counts were centred and row-scaled.

To identify genes that are involved in and dynamically expressed during the process of *M. vibrans* aggregation, we performed a time-series experiment with 24 monoxenic cultures of *M. vibrans* growing with *T. lucentensis*. RNA was extracted and sequenced from four replicate cultures each at different stages along the formation and maturation of aggregates: 12 h, 18 h, 24 h, 33 h, 48 h and 72 h after synchronous inoculation in fresh medium (Fig. 4a, Extended Data Fig. 5a and Supplementary Data 4–6). Aggregates began to form after approximately 18 h in all replicates and continued to grow in size throughout the remainder of the time series, with aggregates becoming more stable in size from approximately 48 h. Validating our experimental approach, analyses of sample relationships revealed that the gene expression profiles from replicate timepoint cultures were most similar to each other, with the greatest variation at 18 h, just before the initiation of aggregation (Extended Data Fig. 5b,c). Replicates taken closer together in time have more similar gene expression profiles, with the largest shifts in gene expression occurring between 12 h and 24 h during the initiation of the aggregation process, and between 33 h and 48 h during the establishment of large mature aggregates.

**Extended Data Fig. 6:**
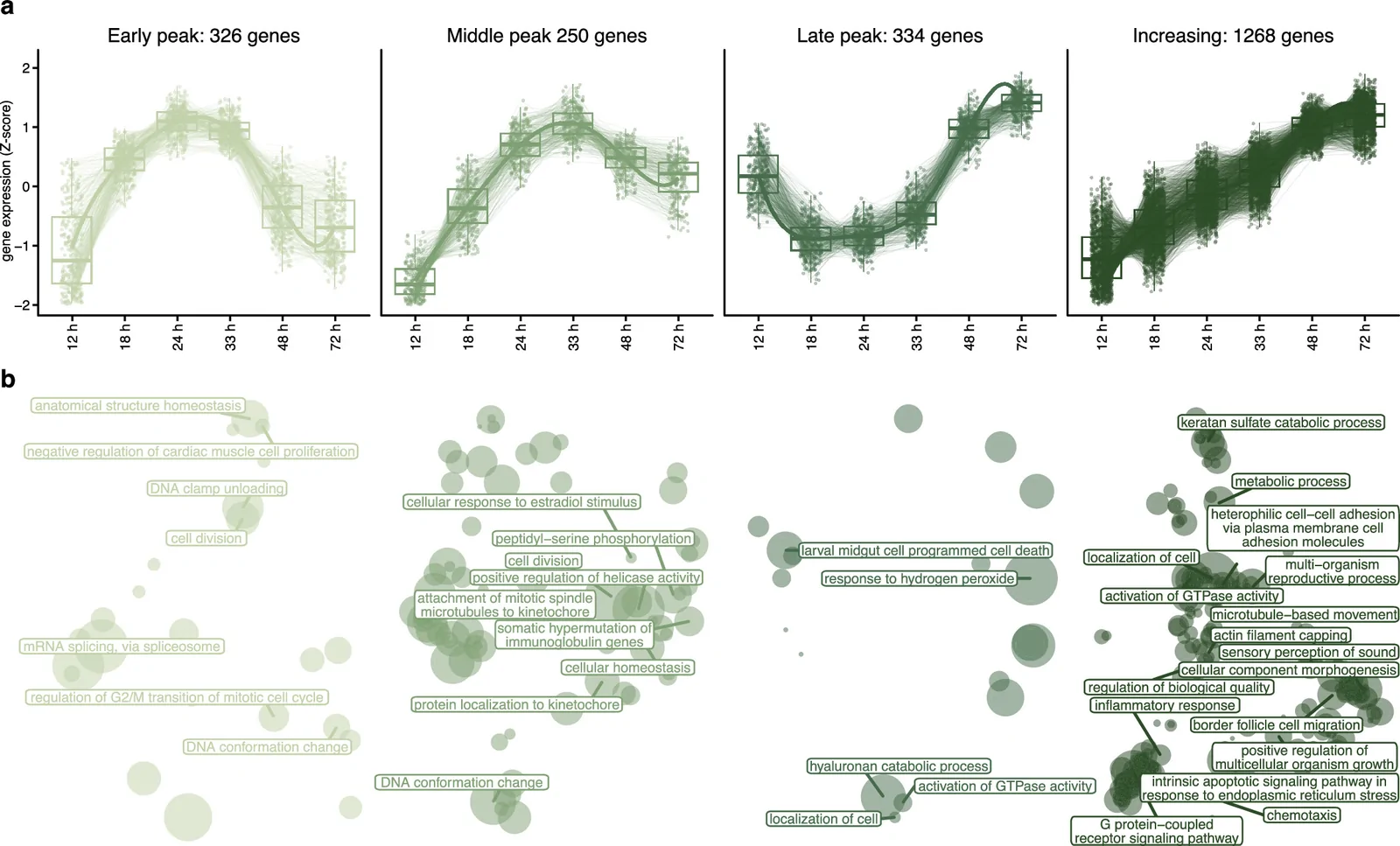
Upregulated groups of genes and enriched GO terms. a) Gene expression trends (Z-score scaled normalized gene counts) of differentially expressed genes (DEG) assigned to the four net upregulated DEG clusters. For the boxplots, the centre line represents the median, the box limits the interquartile range, and whiskers 1.5x the interquartile range. Points indicate individual DEGs, thin lines the expression trends for individual DEGs across the time series, and thick lines the mean expression trend for each DEG cluster. b) Scatterplots showing GO terms enriched in each DEG cluster. Distances between points in the plots indicate the similarity of terms, and the size of points represents their score. The names of the reduced set of GO terms based on semantic similarity and score are indicated.

**Extended Data Fig. 7:**
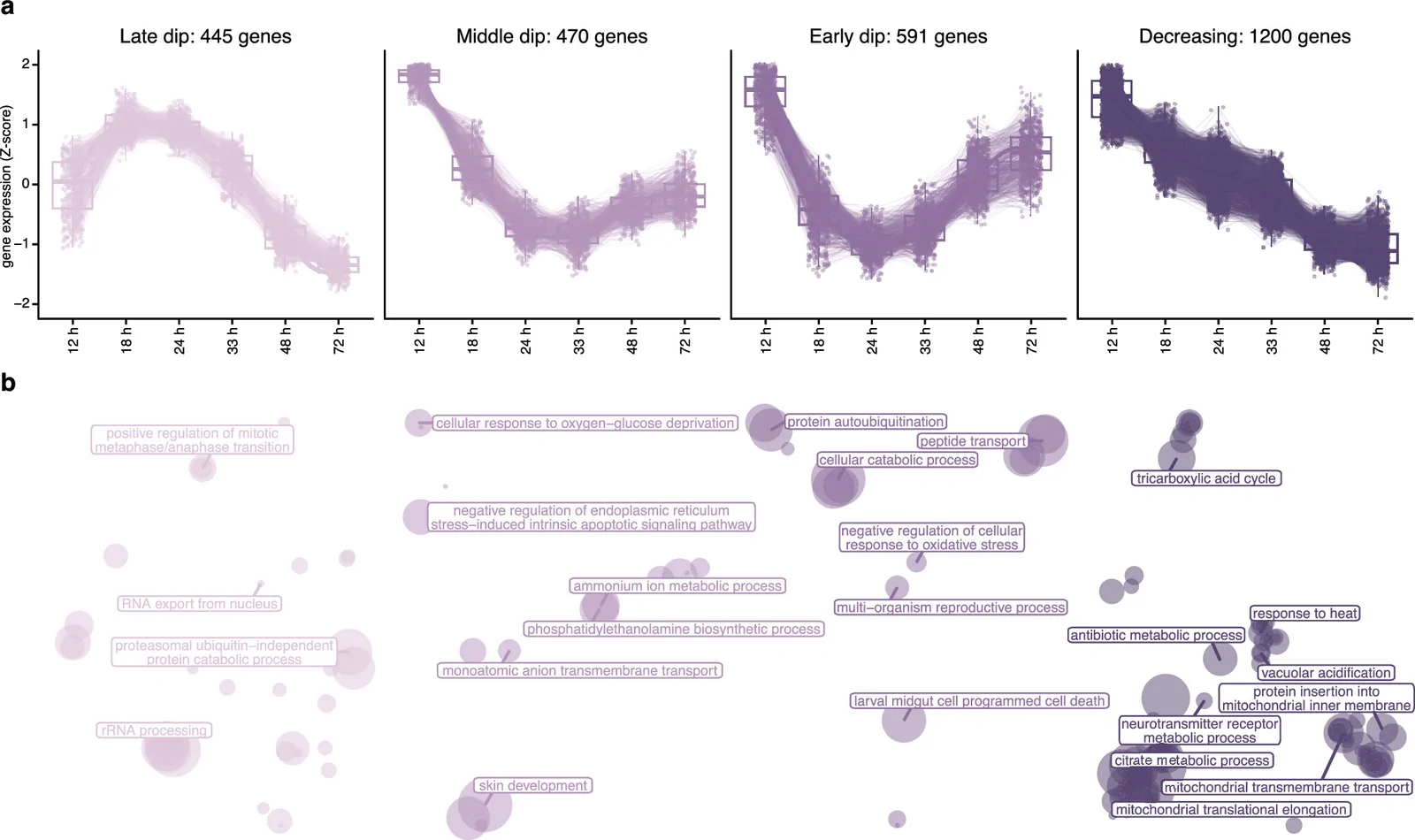
Downregulated groups of genes and enriched GO terms. a) Gene expression trends (Z-score scaled normalized gene counts) of differentially expressed genes (DEGs) assigned to the four net downregulated DEG clusters. For the boxplots, the centre line represents the median, the box limits the interquartile range, and whiskers 1.5x the interquartile range. Points indicate individual DEGs, thin lines the expression trends for individual DEGs across the time series, and thick lines the mean expression trend for each DEG cluster. b) Scatterplots showing GO terms enriched in each DEG cluster. Distances between points in the plots indicate the similarity of terms, and the size of points represents their score. The names of the reduced set of GO terms based on semantic similarity and score are indicated.

To determine which genes drive these shifts during *M. vibrans* aggregation, we identified significantly differentially expressed genes (DEGs) across the time series (Methods and Supplementary Data 7). Of these, 4,884 genes (40.27% of *M. vibrans* genes) formed 8 DEG clusters with overarching patterns in gene expression (Fig. 4b, Extended Data Figs. 5d,e, 6a and 7a and Supplementary Data 7). Four of these DEG clusters are composed of net upregulated genes: early peak (*n* = 326), middle peak (*n* = 250), late peak (*n* = 334) and constantly increasing (*n* = 1,268), whereas another four DEG clusters are composed of net downregulated genes: late dip (*n* = 445), middle dip (*n* = 470), early dip (*n *= 591) and constantly decreasing (*n* = 1,200).

**Extended Data Fig. 8:**
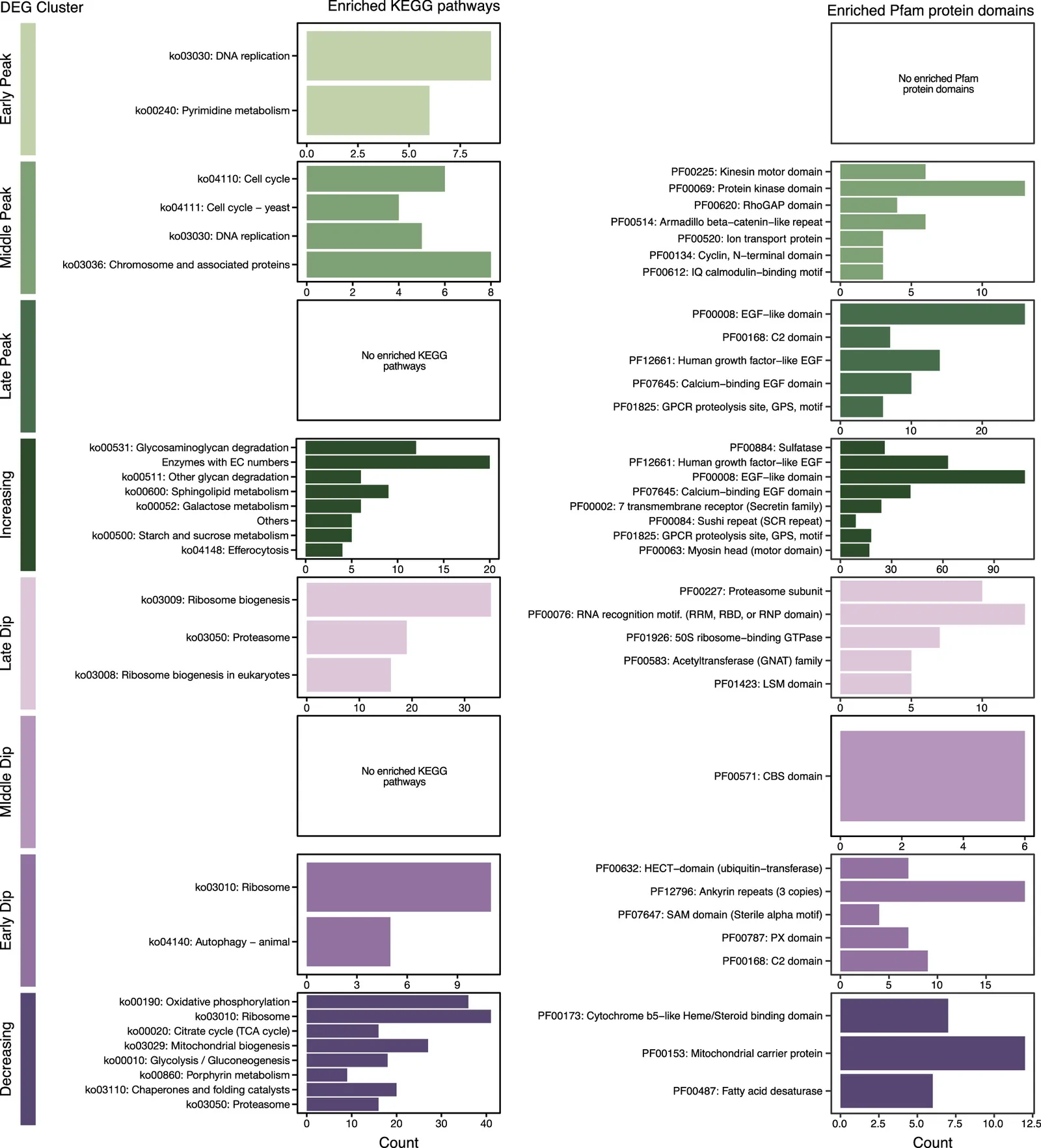
Pathways and protein domains expressed during *M. vibrans* aggregation. Overview of KEGG pathways and Pfam protein domains enriched in each differentially expressed gene (DEG) cluster, indicating which associated functions were expressed with varying patterns throughout the *M. vibrans* aggregation time series. For each plot, pathways and protein domains that meet the significance threshold (adjusted p-value ≤ 0.05) are ordered from lowest to highest adjusted p-value.

We next examined the gene functions enriched in each DEG cluster using Gene Ontology (GO) terms, Kyoto Encyclopedia of Genes and Genomes (KEGG) pathways and Pfam protein domain gene annotations (Fig. 4c, Extended Data Figs. 6b, 7b and 8, Supplementary Figs. 1 and 2, Supplementary Data 8 and Supplementary Discussion 1). Before aggregation, central metabolic, biosynthetic and stress-response functions became downregulated and continued to decrease in gene expression, probably after an initial period of energy gain and growth. Subsequently, the formation of small aggregates was directly preceded by an initial burst in transcription, translation, protein turnover, mRNA splicing and DNA replication. Mitosis, cell division and, unexpectedly, meiosis were upregulated directly after the formation of small aggregates, suggestive of a sexual life stage. Notably, multicellularity-related functions (such as cell adhesion and signalling) continually increased in gene expression as aggregates first formed and then matured, suggesting a key role in *M. vibrans* aggregation. Simultaneously, functions suggestive of the uptake of bacterial prey (related to phagocytosis and negative regulation of immunity) were upregulated. Finally, after the formation of large mature aggregates, cell localization and programmed-cell-death-related functions were upregulated, suggesting the potential for cell rearrangements within aggregates.

Together, these enrichment analyses suggest that key functions relevant to animal origins are involved in *M. vibrans* aggregation. For example, the early upregulation of mRNA splicing functions indicates alternative splicing, which is thought to have provided a source of functional innovation for evolving multicellularity in animals^17^. Moreover, the enrichment of meiosis-related functions during *M. vibrans* aggregation is indicative of a sexual life stage, the integration of which with multicellular life stages is thought to have been an important step in animal origins^18^. Furthermore, cell adhesion, signalling and transcriptional regulation are critical in animal multicellular development^19^. Thus, we next sought to investigate the repertoire of such genes found encoded in the *M. vibrans* genome and to further assess their individual expression profiles.

## Animal multicellularity genes expressed

**Fig. 5:**
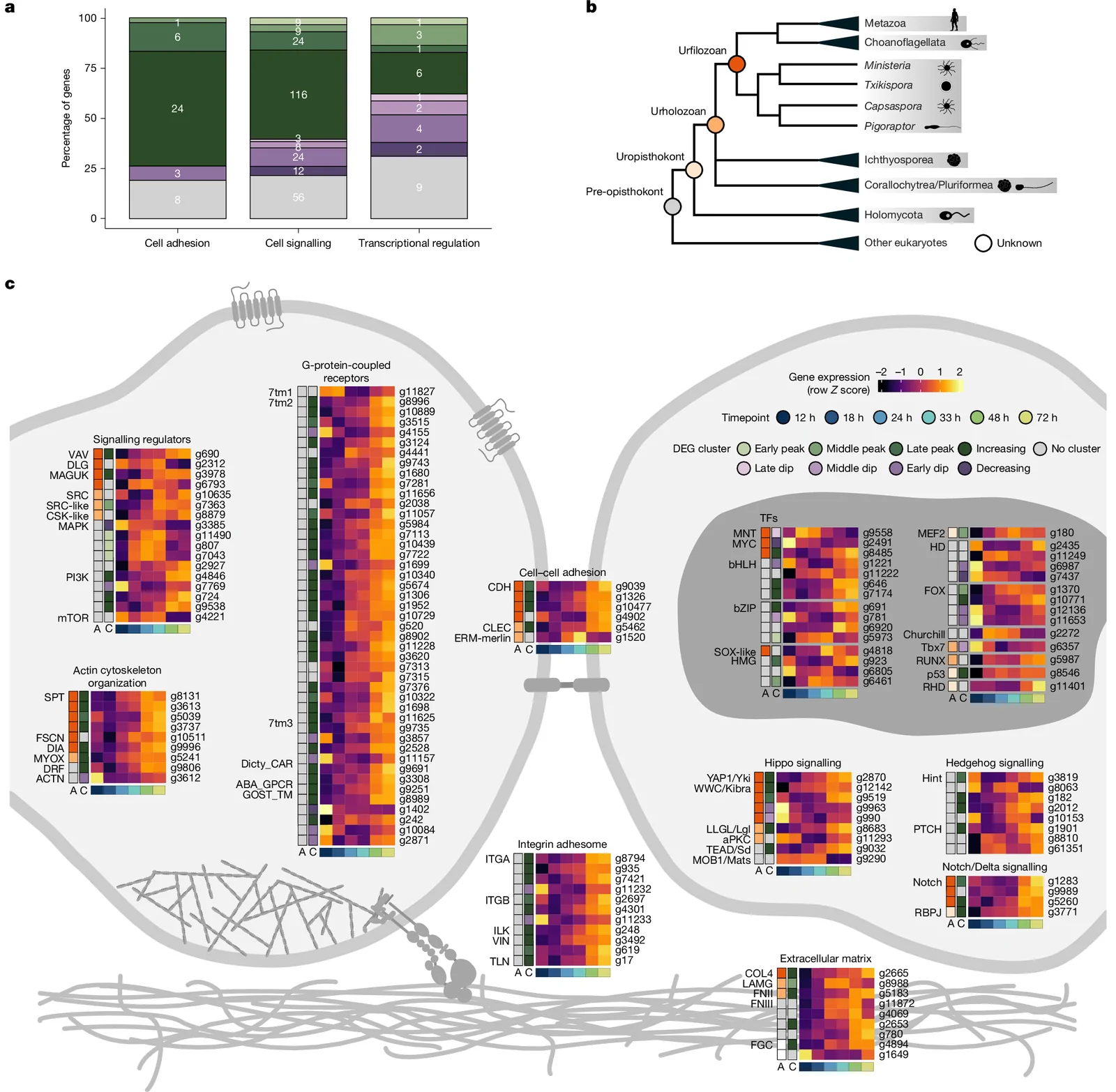
Key animal multicellularity genes are expressed during *M. vibrans* aggregation. **a**, The percentage of DEGs linked to cell adhesion, cell signalling and transcriptional regulation assigned to the DEG clusters outlined in Fig. 4b and coloured according to the legend in **c**. The white numbers indicate the absolute number of DEGs in each category. **b**, Overview of Opisthokonta species relationships and ancestors indicated by coloured circles.** c**, Schematic of two *M. vibrans* cells and heat maps showing the expression patterns of different categories of genes encoding homologues of multicellularity-related proteins that are significantly differentially expressed across the aggregation time series. The bottom row indicates the timepoint; column A denotes the predicted gene/domain age inferred from previous studies, coloured as in **b**, and column C outlines DEG cluster assignment. Mean gene expression values were calculated across timepoint replicates using normalized and variance-stabilizing transformed gene counts that were then centred and row-scaled. Abbreviations of genes, gene families and domain containing proteins: SPT, spectrin; FSCN, fascin; DRF, diaphanous-related formin; DIA, diaphanous DRF; MYOX, myosin X; ACTN, alpha actinin; COL4, alpha type IV collagen; LAMG, laminin G domain; FNII and FNIII, fibronectin type II and type III domains; FGC, fibrinogen C-terminal domain; ITGA, integrin alpha; ITGB, integrin beta; ILK, integrin-linked kinase; VIN, alpha-catenin/vinculin family; TLN, talin; CDH, cadherin domain; CLEC, C-type lectin domain; ERM-merlin, ezrin-radixin-moesin-merlin family; VAV, Vav guanine nucleotide exchange factor; MAGUK, membrane-associated guanylate kinase; DLG, discs large MAGUK; SRC and SRC-like, tyrosine-protein kinase c-Src and c-Src like; CSK-like, C-terminal Src kinase like; MAPK, mitogen-activated protein kinase; PI3K, phosphatidylinositol 3-kinase; mTOR, mechanistic target of rapamycin; 7tm1, rhodopsin/class A GPCR domain; 7tm2, adhesion/secretin/class B GPCR domain; 7tm3, glutamate/class C GPCR domain; Dicty_CAR, cAMP/classE GPCR domain; ABA_GPCR, abscisic acid receptor GPCR domain; GOST_TM, GOST seven transmembrane GPCR domain; YAP1/yki, yes-associated protein 1/yorkie; WWC/Kibra, WW and C2 domain containing/kidney and brain expressed protein; LLGL/Lgl, lethal 2 giant larvae; aPKC, atypical protein kinase C; TEAD/Sd, TEA domain TF/scalloped; MOB1/Mats, MOB kinase activator 1/mob as tumour suppressor; Hint, Hint module domain; PTCH, patched domain; Notch, notch domain; RBPJ, recombining binding protein suppressor of hairless TF; bHLH, basic helix loop helix TF; MNT, MAX network transcriptional repressor bHLH; MYC, MYC proto-oncogene bHLH; bZIP, basic leucine zipper TF; HMG, high-mobility group box TF; SOX-like, SRY-related HMG like; MEF2, myocyte-specific enhancer factor 2 MADS-box TF; HD, homeobox TF; FOX, forkhead TF; RUNX, runt-related TF; RHD, rel homology DNA-binding TF. See also Extended Data Fig. 9, Supplementary Fig. 3 and Supplementary Data 9. The diagram in **b** was created using BioRender; Gerdt, J. P. https://BioRender.com/bitcuve (2026).

Using reciprocal best hits against human homologues, top hits to *C. owczarzaki* homologues, the presence of protein domains and functional annotations, we identified 521 genes in the *M. vibrans* genome encoding putative homologues of proteins involved in multicellularity and development in animals, and more specifically linked to cell adhesion (*n* = 68), cell signalling (*n* = 400) and transcriptional regulation (*n* = 53) (Supplementary Data 9 and Supplementary Discussion 2). Of these, 332 changed significantly in expression across the *M. vibrans* aggregation time series. Of those assigned to DEG clusters, 92%, 77% and 55% of cell adhesion, cell signalling and transcriptional regulation related genes, respectively, were assigned to net upregulated DEG clusters (Fig. 5a). Many of these genes encode proteins that are critical for multicellularity in animals. Some were previously thought to be animal specific, but are now known to have deeper evolutionary roots, with estimated gene ages tracing back to pre-opisthokont, uropisthokont, urholozoan and urfilozoan ancestors^1^ (Fig. 5b,c and Supplementary Data 9).

**Extended Data Fig. 9:**
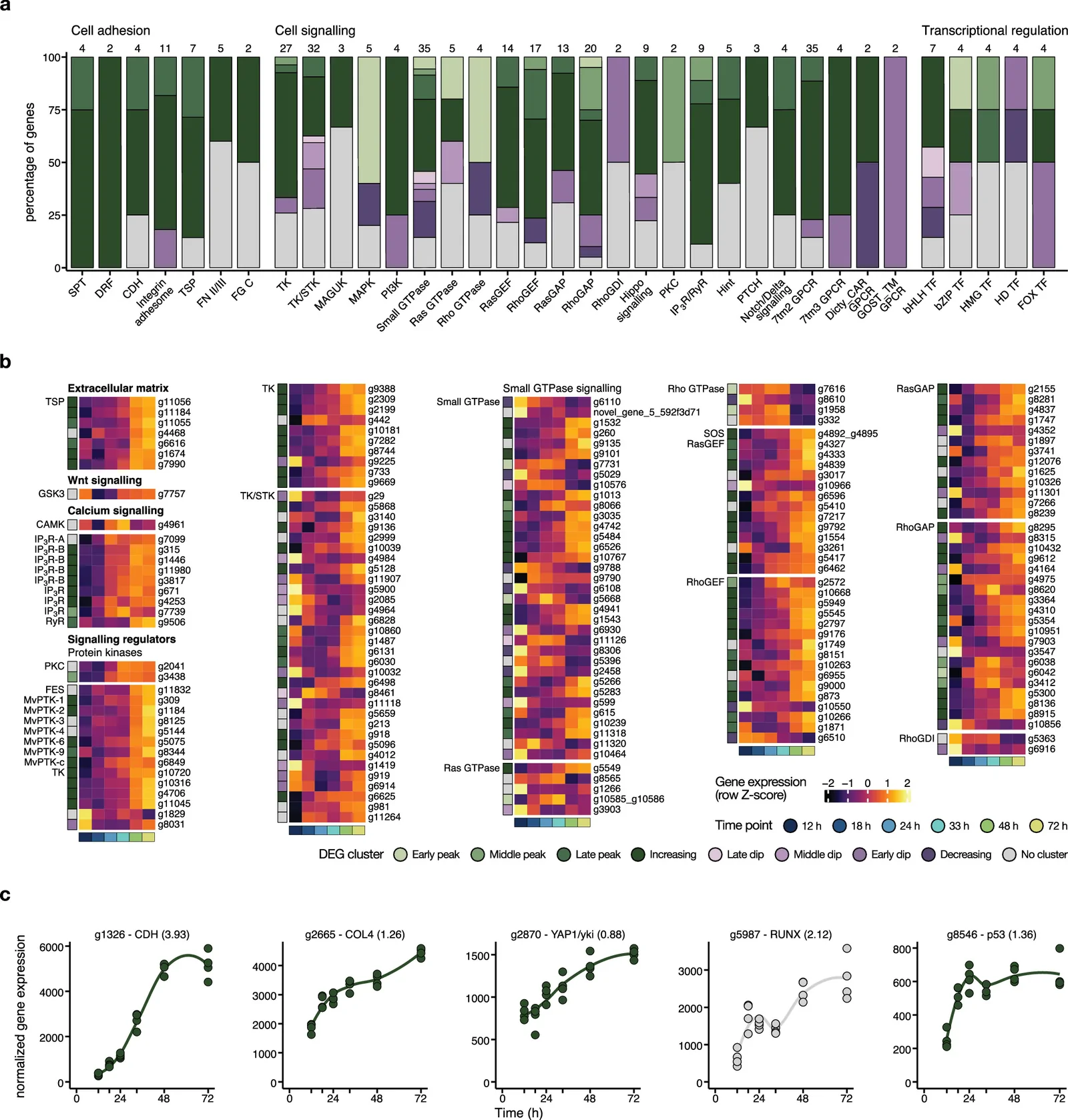
Diverse categories of *M. vibrans* multicellularity-related genes are differentially expressed during aggregation. a) Percentage of differentially expressed genes (DEGs) assigned to each DEG cluster from each group of genes encoding proteins related to cell adhesion, cell signalling, and transcriptional regulation. Numbers at the top indicate the number of DEGs in that category. Only groups with at least two DEGs are shown. b) Heatmaps showing the expression patterns of additional genes encoding proteins involved in animal multicellularity that are significantly differentially expressed across the *M. vibrans* aggregation time series. The bottom row indicates the time point according to the legend and the left column DEG cluster assignment. Mean gene expression values were calculated across time point replicates using normalized and variance-stabilizing transformed gene counts that were then centred and row-scaled. Abbreviations used for genes, gene families, and domain containing proteins: thrombospondin domain, TSP; glycogen synthase kinase 3, GSK3; Ca^2+^/calmodulin-dependent protein kinase, CAMK; inositol 1,4,5-trisphosphate receptor, IP_3_R; ryanodine receptor, RyR; protein kinase C, PKC; tyrosine-protein kinase Fes/Fps, FES; *M. vibrans* protein-tyrosine kinase, MvPTK; protein-tyrosine kinase, TK; protein-tyrosine and serine/threonine kinase domain, TK/STK; Ras and Rho guanine nucleotide exchange factor, RasGEF and RhoGEF; Son of Sevenless RasGEF, SOS; Ras and Rho GTPase-activating protein, RasGAP and RhoGAP; Rho guanine nucleotide dissociation inhibitor, RhoGDI. See Fig. 5 for additional definitions and Data S9 for gene annotation information, protein domains, and corresponding genes in *Capsaspora owczarzaki* where relevant. c) Plots of normalized gene expression across the aggregation time series for selected upregulated genes encoding proteins with diverse functions and filozoan or holozoan inferred origins. Log2 fold change between 12 and 72 h is indicated in brackets. See also Fig. S3 and Data S9.

First, we observed a significant upregulation of several adhesion-related genes, including homologues of proteins responsible for building cellular scaffolding (that is, actin cytoskeleton organization), the extracellular matrix (ECM), cell–ECM adhesion and cell–cell adhesion in animals (Fig. 5, Extended Data Fig. 9, Supplementary Fig. 3, Supplementary Data 9 and Supplementary Discussion 2). These genes include spectrin, fascin and diaphanous homologues, inferred to have originated by the urfilozoan ancestor, and myosin X tracing back to the urholozoan ancestor. In animals, these genes function in actin filament, epithelia and filopodia formation^20^. Diverse ECM-related genes were also upregulated, including alpha type IV collagen—to date the only case known outside Metazoa^17^. The integrin adhesome has pre-opisthokont origins and is central in animal cell–ECM adhesion^21^. Most components were upregulated together with homologues of kinases that function in integrin-mediated signalling. Upregulated genes involved in cell–cell adhesion in animals included a C-type-lectin-domain-containing protein (not previously found in Filasterea) and a homologue of the ezrin-radixin-moesin-merlin family, inferred to have originated by the urholozoan ancestor^22^. Moreover, we found that *M. vibrans* encodes 8 cadherin domain-containing proteins, several of which were upregulated and increased up to 15 times in gene expression. Holozoan-type cadherin domain-containing proteins are currently only found in Filozoa and only one is encoded in the *C. owczarzaki* genome^23^.

We next observed the activation of numerous cell signalling pathways critical in animals (Fig. 5, Extended Data Fig. 9, Supplementary Fig. 3, Supplementary Data 9 and Supplementary Discussion 2). These signalling mechanisms coordinate animal cell behaviour and ensure the formation of a cohesive unit, indicating a potential for coordination and communication between aggregating *M. vibrans* cells. Many tyrosine kinases, mitogen-activated protein kinases and phosphatidylinositol-3-kinases were differentially expressed, alongside putative membrane-associated guanylate kinases, which are inferred to have originated by the urfilozoan ancestor and regulate cell–cell adhesion in animals^24,25^. Small GTPases had variable expression patterns, while the majority of guanine nucleotide exchange factors (GEFs), including a putative Vav family GEF that was to date found only in Filozoa^26^, and GTPase-activating proteins were upregulated. By contrast, guanine nucleotide dissociation inhibitors were downregulated. Furthermore, the majority of differentially expressed G-protein-coupled receptors (GPCRs) were upregulated. In particular, these included adhesion and secretin class B GPCRs, the number of which encoded by *M. vibrans* is exceptional outside of Metazoa^27^. All genes encoding a subset of the typical protein domains found in Notch proteins, previously found across Filozoa^28^, were also upregulated, alongside a putative RBPJ transcription factor (TF). In animals, Notch and RBPJ are involved in Notch–Delta signalling. The Hippo signalling pathway regulates tissue size and most of the components identified in *M. vibrans* were differentially expressed, including Yorkie (Yes-associated protein 1), which is inferred to have originated by the urfilozoan ancestor^29^. The Hedgehog signalling pathway regulates cell differentiation, and most of its components are animal specific^30^. However, protein domains from some key pathway genes are found outside of Metazoa, such as the Hedgehog hint module and patched^31,32^. We found several genes encoding each of these two protein domains, most of which were differentially expressed.

Finally, we found that a large collection of TFs are differentially expressed in *M. vibrans*, including many involved in transcriptional regulation during animal development (Fig. 5, Extended Data Fig. 9, Supplementary Fig. 3, Supplementary Data 9 and Supplementary Discussion 2). In contrast to cell adhesion and signalling, a nearly equal number of differentially expressed TFs were net upregulated and downregulated across the *M. vibrans* aggregation time series. The individual expression profiles of these TFs were diverse, revealing a complex regulatory system with which to deploy multicellularity genes in *M. vibrans*. A number of differentially expressed *M. vibrans* TFs are relatively recent innovations. T-box, Rel homology DNA-binding and p53 TFs are inferred to have originated in the uropisthokont ancestor, runt-related in the urholozoan ancestor and Myc–Max network, and SRY-related HMG-box like (SOX-like) TFs in the urfilozoan ancestor^33,34^. The expression of these TFs during *M. vibrans* aggregation suggests that they were already regulating multicellularity in unicellular animal ancestors.

We caution that these major gene expression changes simply correlate with *M. vibrans* aggregation. To determine which upregulated genes are necessary to form and maintain aggregates, functional validation is required. Methods to genetically manipulate *M. vibrans* are being developed to enable these studies. A plausible outcome is that many upregulated genes are not necessary to form aggregates, but instead optimize the morphology of the aggregates and behaviours of cells in an aggregated state^35^.

Certainly some sets of animal multicellularity-related genes have previously been found upregulated in different cell types of diverse unicellular animal relatives^36–38^. Yet, *M. vibrans* is exceptional in expressing homologues of nearly the full complement of such genes during aggregation. Although we do not know the function of most of these genes in a non-animal context, several have been found to have analogous functions relevant for animal multicellularity^35,39^.

Together, we found that components of most cell adhesion and cell signalling pathways found in *M. vibrans* were differentially expressed and upregulated across the aggregation process. Homologues of the various TFs involved in animal development were also differentially expressed with diverse expression patterns indicating coordination of various phases of *M. vibrans* aggregation. Moreover, gene homologues with more recent evolutionary origins in Opisthokonta, Holozoa and Filozoa were differentially expressed and most often upregulated. Our results show that an extensive gene repertoire key in animals is also involved in the development of *M. vibrans* aggregates. This finding suggests conservation of mechanisms for cell adhesion, signalling and transcriptional regulation between *M. vibrans* aggregation, and animal multicellularity.

## Aggregation improves feeding and mating

**Fig. 6:**
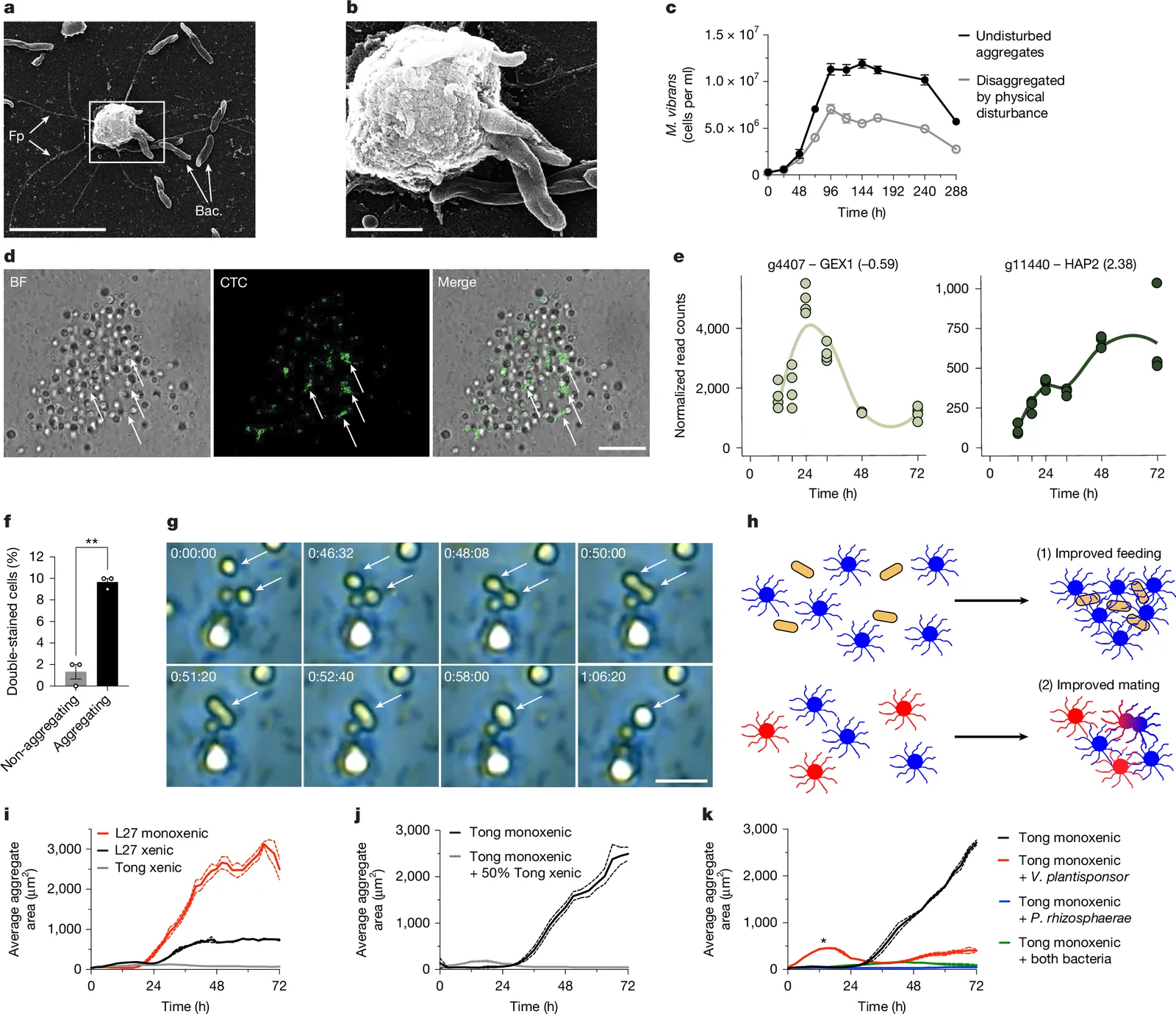
Evolutionary drivers of aggregation. **a**, SEM image revealing *M. vibrans* engulfing *T. lucentensis* bacteria. Fp, filopodia; Bac., bacteria. Representative of 32 images.** b**, Magnification of the white box in **a**.** c**, Cell counts of *M. vibrans* in cultures with/without physical disruption of aggregates. Data are mean ± s.e.m. of a biological quadruplicate.** d**, Bright-field (BF) and confocal fluorescence images of *M. vibrans* aggregates in monoxenic *T. lucentensis* culture. Bacteria were stained with the fluorescent dye CTC. Regions of CTC-stained bacterial biofilm between *M. vibrans* cells are highlighted by white arrows. The images are representative of approximately 1–3 aggregates viewed per well of three biological replicates.** e**, Normalized gene expression of GEX1 and HAP2 during the time course of aggregation. The log_2_-transformed fold change between 12 and 72 h is indicated in brackets.** f**, The percentage of double-stained cells after mixing two independently stained populations in aggregating and non-aggregating conditions. Data are mean ± s.e.m. of a biological triplicate. Individual replicates are displayed with circles. Statistical analysis was performed using an unpaired two-sided *t*-test with Welch’s correction; *P* = 0.0017.** g**, Snapshots of two cells fusing under aggregating conditions (from Supplementary Video 5). The white arrows indicate two single cells that merged with each other. Time format, h:min:s.** h**, Schematic of the potential benefits of aggregative multicellularity for *M. vibrans* (and its ancestors).** i**, The average aggregate area quantified for the *M. vibrans* L27 (DSM 114853) strain in xenic culture and in monoxenic culture with *T. lucentensis*, compared with the non-aggregating xenic Tong culture monitored for 72 h. See Supplementary Videos 6 and 7.** j**, The average aggregate area quantified for the *M. vibrans* Tong strain in monoxenic culture with *T. lucentensis* or 1:1 *T. lucentensis* monoxenic culture and xenic culture, monitored for 72 h.** k**, The average aggregate area quantified for the *M. vibrans* Tong strain in monoxenic culture with *T. lucentensis* or dixenic or trixenic culture with *T. lucentensis* and *V. plantisponsor* and/or *P. rhizosphaerae* at their approximate abundances in the xenic culture (63%, 24% and 5%, respectively), monitored for 72 h. The asterisk indicates clumped bacteria, not aggregated *M. vibrans*. For **i**–**k**, data are mean (solid line) ± s.e.m. (dashed line) of a biological quadruplicate. See also Extended Data Fig. 10 and Supplementary Fig. 5. Scale bars, 1 μm (**b**), 5 μm (**a**), 10 μm (**g**) and 20 μm (**d**). The diagram in **h** was created using BioRender; Gerdt, J. P. https://BioRender.com/bitcuve (2026).

**Extended Data Fig. 10:**
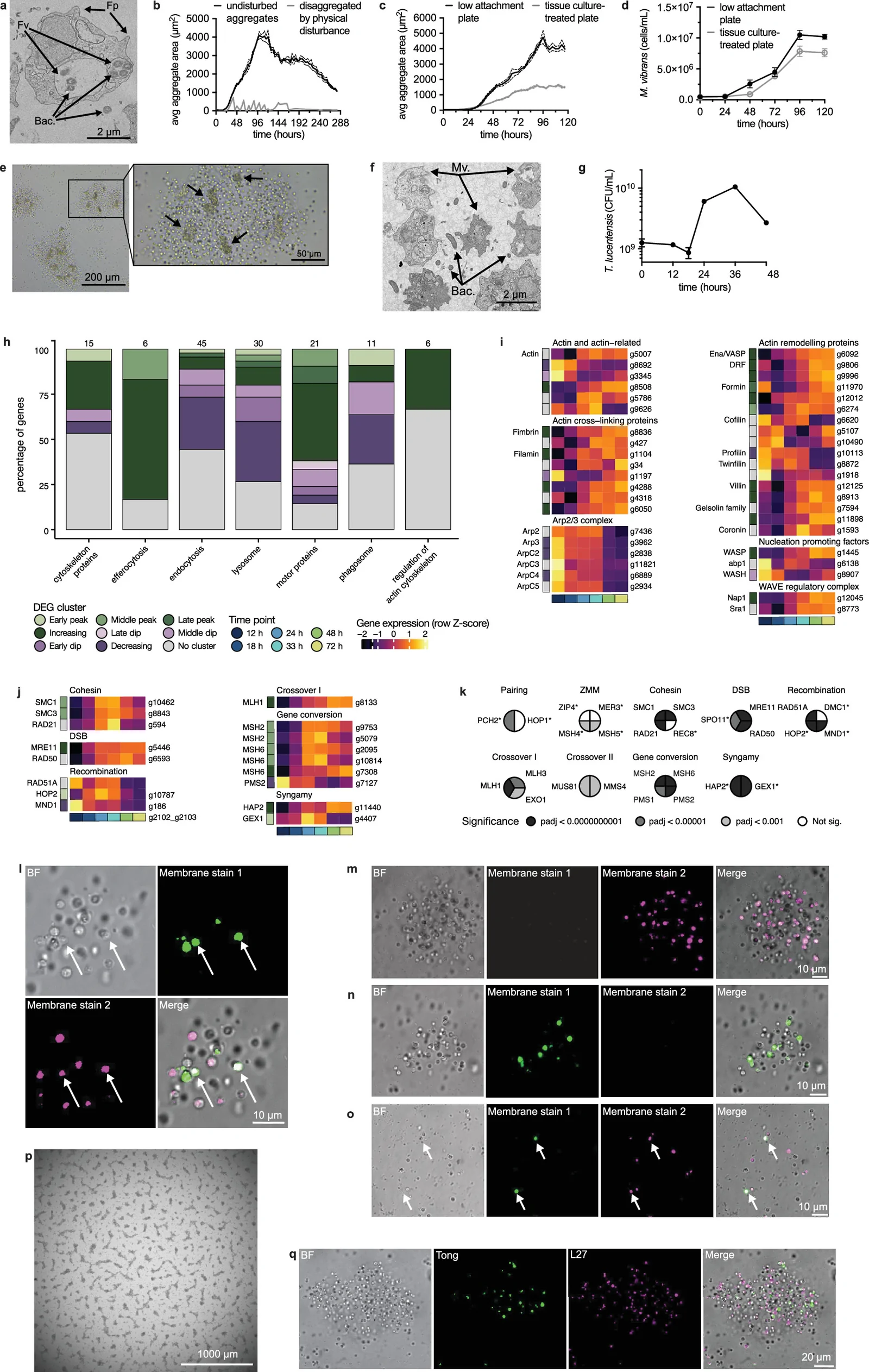
*M. vibrans* consumes bacteria and shows signatures of mating. a) Representative transmission electron micrograph revealing *T. lucentensis* bacteria within *M. vibrans* vacuoles (Fp = filopodia, Bac. = bacteria, Fv = food vacuole). Images are representative of 21 images of cells observed with bacteria in food vacuoles. b) Average aggregate area quantified over time for *M. vibrans* cultures with/without physical disruption of aggregates. Dashed lines represent standard error of the mean (SE) of a biological quadruplicate. c) Average aggregate area quantified over time for *M. vibrans* cultures on low attachment plates and tissue culture-treated (high attachment) plates. Dashed lines represent standard error of the mean (SE) of a biological quadruplicate. d) Cell counts of *M. vibrans* in cultures growing as large aggregates in low attachment plates or much smaller aggregates in high attachment (tissue culture-treated) plates. Error bars represent SE of a biological quadruplicate. e) Representative brightfield microscopy image of *M. vibrans* aggregates with putative bacterial biofilm material embedded within (highlighted with black arrows). Images are representative of 7-12 aggregates viewed per well of three biological replicates. f) Representative transmission electron micrograph revealing *T. lucentensis* bacteria in the intercellular space of an *M. vibrans* aggregate (Mv = *M. vibrans* cell, Bac = bacteria). Images are representative of 10 images of bacteria observed within the intercellular space of aggregates. g) Colony-forming units of *T. lucentensis* food bacteria over time in the aggregating *M. vibrans* culture. Error bars represent standard error of the mean (SE) of a biological triplicate. h) Percentage of differentially expressed genes (DEGs) from phagocytosis-related KEGG pathways assigned to each DEG cluster. i-j) Heatmaps showing the expression patterns of genes encoding proteins related to i) phagocytosis and j) meiosis and syngamy significantly differentially expressed across the aggregation time series. The bottom row indicates the time point according to the legend and the left column DEG cluster assignment. Mean gene expression values were calculated across time point replicates using normalized and variance-stabilizing transformed gene counts that were then centred and row-scaled. k) An overview of genes encoding key meiosis and syngamy related proteins searched for in the *M. vibrans* genome (all except REC8 detected). Those found to be differentially expressed during aggregation based on different adjusted p-value significance level cutoffs are coloured according to the legend. Statistical significance was assessed using the likelihood ratio test (LRT) as implemented in DESeq2^92^, with p-values adjusted for multiple testing using independent hypothesis weighting (IHW)^95^. See Data S10 for exact adjusted p-values. An asterisk indicates proteins specific to meiosis or syngamy. Definitions of abbreviations used in the figure: Diaphanous-related formin, DRF; homologue pairing, pairing; ZMM complex, ZMM; sister chromatid cohesion, cohesin; introduction of double-stranded breaks, DSB; homologous recombination, recombination; cross-over resolution pathway I and pathway II, crossover I and crossover II; gene conversion by mismatch repair, gene conversion. See also Fig. S5–6, Data S10, and Supplementary Discussion 3. l) Representative brightfield (BF) and confocal fluorescence images of a *M. vibrans* aggregate formed by mixing two independently stained monoxenic *T. lucentensis* cultures. White arrows indicate two cells that appear to contain both stains, suggesting that cells might fuse in the aggregation-inducing conditions. Images are representative of 5-12 aggregates viewed per well of three biological replicates. m–o) Representative brightfield (BF) and confocal fluorescence images of a *M. vibrans* aggregate formed by mixing two independently stained monoxenic *T. lucentensis* cultures. Panel (m) was generated by inducing aggregates with only the first stained population. Images are representative of 1-2 aggregates viewed in each of 5 images across three biological replicates. Panel (n) was generated by inducing aggregates with only the second stained population. Images are representative of 1-3 aggregates viewed in each of 5 images across three biological replicates. Panel (o) was generated by inducing aggregates with a mixture of both stained populations, as in panel (l), but first the aggregates were dispersed into individual single cells for imaging. White arrows indicate two cells that appear to contain both stains, indicating that cells fused in the aggregation-inducing conditions. Images are representative of 6 images per well in three biological replicates. p) Representative brightfield (BF) image of the xenic *M. vibrans* L27 culture presented in Fig. 6i. Image is 72 h after dilution into fresh growth media. Image is representative of biological quadruplicate (one image per replicate well). q) Representative brightfield (BF) and confocal fluorescence images of a mixed-strain *M. vibrans* aggregate formed by mixing the monoxenic cultures of the Tong and L27 strains of *M. vibrans*. Each strain was first independently stained with a different colour membrane dye (BioTracker 490 Green or 555 Orange) before mixing. Images are representative of 3-8 aggregates viewed per well of three biological replicates.

We next examined what advantage aggregation offers *M. vibrans*. For unicellular organisms, aggregation can have multiple benefits, including improved feeding or mating^40,41^. *M. vibrans* is a known bacteriovore^16^, and our monoxenic culture exhibited clear phagocytosis of the *T. lucentensis* food bacteria (Fig. 6a,b and Extended Data Fig. 10a). Furthermore, we observed that the aggregating monoxenic culture grew better than the non-aggregating xenic culture (Fig. 2a). The correlation between growth and aggregation led us to hypothesize that aggregation promotes *M. vibrans* replication. To test this hypothesis, we prevented aggregate maintenance through physical disruption (Extended Data Fig. 10b). We then examined whether disaggregated cells grew worse than those that remained in aggregates. Indeed, disaggregated cells replicated worse than those left undisturbed (Fig. 6c), suggesting a fitness advantage to aggregating. However, the physical disruption of aggregates may directly inhibit *M. vibrans* growth—possibly by displacing pre-phagocytosed bacteria from *M. vibrans* cell surfaces or inducing a different stress. Alternatively, we inhibited aggregation without physical disruption by growing the *M. vibrans* culture on tissue-culture-treated surfaces, to which *M. vibrans* attaches more tightly and aggregates less compared with the standard low-attachment surface used in other experiments (Extended Data Fig. 10c). Although the effect was more subtle, this condition with decreased aggregate size also exhibited diminished *M. vibrans* replication (Extended Data Fig. 10d). This experiment also carries a caveat—*M. vibrans* cells move slower on tissue-culture-treated surfaces, which may decrease their encounters with bacteria and at least partially explain the decreased rate of growth. Thus, our findings are consistent with aggregation promoting *M. vibrans* growth; however, we cannot rule out the involvement of confounding effects in these experiments.

A possible explanation for the improved replication is an increase in food uptake. Cells in the middle of an aggregate may not have access to bacteria, which would actually inhibit feeding. However, as aggregates form and morph, they may also entangle bacteria within their community, providing more efficient feeding. In support of this hypothesis, we have observed material embedded within aggregates that resembles bacterial biofilms (Extended Data Fig. 10e). This material stained well with 5-cyano-2,3-ditolyl tetrazolium chloride (CTC), which stains metabolically active bacteria (Fig. 6d). Furthermore, transmission electron microscopy (TEM) revealed bacterial cells within the intercellular space of *M. vibrans* aggregates (Extended Data Fig. 10f). We have also observed that the number of *T. lucentensis* food bacteria in the culture and number of mapped reads increased with the formation of small aggregates, before dropping shortly after *M. vibrans* formed large aggregates (Extended Data Fig. 10g and Supplementary Fig. 4). The formation of aggregates and drop in *T. lucentensis* counts also correlated with the expression of phagocytosis-associated genes in *M. vibrans*, with initially high expression of the Arp2/3 complex, followed by later upregulation of actin cross-linking and remodelling proteins (Extended Data Fig. 10h,i, Supplementary Fig. 5, Supplementary Data 10 and Supplementary Discussion 3). Further work is required to fully test this hypothesis, but aggregation may improve the efficiency of bacterial capture by *M. vibrans*.

Moreover, aggregates may promote opportunities for mating between cells, as shown in diverse organisms, including the choanoflagellate *S. rosetta*, in which mating is induced by starvation or *Vibrio fischeri* bacteria^3,42^. Mating can improve fitness by transmitting beneficial genetic traits, spawning beneficial combinations of genes^43^ and providing adaptive flexibility^44^. Indeed, we found that a variety of genes encoding proteins associated with both meiosis and mating (specifically syngamy)^45^ were differentially expressed during the aggregation process. They were most often upregulated in small aggregates (Fig. 6e, Extended Data Fig. 10j–k, Supplementary Figs. 5 and 6 and Supplementary Discussion 3), suggesting another plausible benefit for aggregation. Such meiosis-related proteins found differentially expressed at different significance cut-offs include those involved in synaptonemal complex assembly (PCH2, MSH4 and MSH5), sister chromatid cohesion (SMC1, SMC3 and RAD21), introducing double-stranded breaks (SPO11, MRE11 and RAD50), homologous recombination (RAD51, HOP2 and MND1), crossover resolution pathways I (MLH1, MLH3 and EXO1) and II (MYS81, and MMS4), and gene conversion by mismatch repair (MSH2, MSH6, PMS1 and PMS2). Several of these are considered to be meiosis specific (that is, PCH2, MSH4, MSH5, SPO11, HOP2 and MND1). We also found that the syngamy-specific genes encoding the proteins GEX1 and HAP2, which are involved in cell nuclear and membrane fusion, were differentially expressed during aggregation. To test for cell fusion in aggregation-induction conditions, we induced aggregation of a mixture of two populations of *M. vibrans* stained with different fluorescent membrane dyes. We observed that around 10% of the stained cells were stained by both dyes (Fig. 6f and Extended Data Fig. 10l–o), suggesting that cells can fuse in the aggregation-inducing conditions. By contrast, fewer than 2% of stained cells were stained by both dyes under non-aggregation conditions (Fig. 6f), suggesting that aggregation promotes cell fusion. We considered the possibility that the double-staining occurred due to cannibalism instead of mating. In that case, we might expect one of the dyes to be localized to a food vacuole. However, we constructed 3D image stacks of several double-stained cells and found that both dyes spread throughout each cell (Supplementary Video 4 is representative of 16 imaged cells). Although we cannot rule out the possibility that dyes distribute from the food vacuole over the course of the experiment, our observation of widespread staining supports the hypothesis that plasma membranes and cytosols are fusing. Finally, we took time-lapse videos focused on the edge of aggregates to observe cell fusion events. Indeed, we observed one clear fusion of two cells after reviewing a total of around 216 cells for about 2.7 h each (Fig. 6g and Supplementary Video 5). Fusion events may be more frequent within the cores of aggregates, but these areas are challenging to image. Although further investigation will definitively test whether meiosis and mating are occurring in aggregates, the combined evidence of meiotic gene upregulation and increased rates of cell fusions in aggregation conditions provide a compelling signature of mating.

Although not conclusive, our results suggest that improved feeding and increased meiosis and mating may occur within *M. vibrans* aggregates. These behaviours might be considered in conflict, as mating is often considered to be a stress response in unicellular organisms^42^. However, mating has been observed in the choanoflagellate *S. rosetta* under healthy growth conditions in the presence of *V. fischeri*^3^. Furthermore, our transcriptomic analysis pooled the entire population of cells. It may be that a subset of cells is highly expressing meiosis/mating genes and not actively dividing. In other words, aggregation may cause a bimodal effect whereby different cell types (mating and dividing) are formed. These hypotheses warrant extensive further investigation. In summary, improved feeding and cell division and/or increased mating may have been evolutionary drivers benefiting aggregative multicellularity in the unicellular ancestors of animals (Fig. 6h).

## A xenic *M. vibrans* culture aggregates

We next examined the natural relevance of this aggregation behaviour, because we had observed it only in a single monoxenic culture of one *M. vibrans* isolate. To further examine its frequency, we obtained a second environmental isolate of *M. vibrans* (strain L27, DSM 114853)^16^. This strain grew poorly in the recommended growth medium (F2 medium). It also contained two separate eukaryotic organisms (*M. vibrans* and *Paraphysomonas imperforata*; Supplementary Video 6 and Supplementary Data 11). After isolating *M. vibrans* (along with its xenic bacterial population), we observed good growth in 5% seawater complete (SWC) medium. Furthermore, under our improved growth conditions, we observed clear aggregation of the *M. vibrans* cells, although the average aggregate area was smaller than the monoxenic culture generated in this study (Figs. 3a and 6i, Extended Data Fig. 10p and Supplementary Video 7). We also generated a monoxenic culture of *M. vibrans* L27 with *T. lucentensis*, which formed larger aggregates than the xenic culture (Fig. 6i, Supplementary Video 8 and Supplementary Data 12). As the only two known isolates of *M. vibrans* can both grow as aggregates in culture, we conclude that this phenotype is widespread and relevant for the lifestyle of *M. vibrans* in natural environments. In fact, when we mixed the two monoxenic cultures together, the Tong and L27 strains aggregated together in mixed aggregates (Extended Data Fig. 10q). This observation suggests that the mechanism of aggregation is conserved across *M. vibrans*.

We reiterate that the L27 xenic culture (containing at least 14 different natively isolated bacteria; Supplementary Data 13) formed clear aggregates (Extended Data Fig. 10p). None of those bacteria was a member of the *Thalassospira* genus. The closest relative was a minor component (2.7%) annotated as a distantly related alphaproteobacterium (*Sulfitobacter*). Thus, monoxenic culture with a specific bacterial genus is not a requirement for aggregation. The fact that this more ‘natural’ xenic bacterial community allowed aggregation provides further evidence that aggregation may be a common state of *M. vibrans* in nature.

## Bacteria inhibit *M. vibrans* aggregation

Finally, we sought initial insights into why *M. vibrans* Tong failed to aggregate in xenic culture. One possibility is that *T. lucentensis* may share aggregation-inducing traits with bacteria that are present in the xenic *M. vibrans* L27 culture. Alternatively, aggregation-inhibiting bacteria may be uniquely present in the *M. vibrans* Tong xenic culture. These hypotheses are not mutually exclusive.

The second hypothesis (that inhibitory bacteria are present in the xenic culture) is supported by the observation that *T. lucentensis* already composed 63% of the bacteria in the Tong xenic culture. Indeed, re-addition of the xenic culture to the monoxenic culture at a 1:1 ratio eliminated aggregation (Fig. 6j). It seemed unlikely to us that the relatively modest change from 100% to 60% *T. lucentensis* would cause such a substantial change in *M. vibrans* aggregation. Instead, we suspected that the presence or complete absence of a moderately abundant aggregation-inhibiting bacterium was more likely responsible for the difference. To test this hypothesis, we isolated the next two most abundant bacteria from the xenic culture (*Vibrio plantisponsor* and *Pseudoalteromonas rhizosphaerae*; Supplementary Data 1) and added them to the monoxenic culture at their approximate levels in the xenic culture (24% and 5%, respectively). In the presence of *V. plantisponsor*, aggregates were smaller and took longer to develop (Fig. 6k). In the presence of even a low percentage of *P. rhizophaerae*, aggregation was eliminated (regardless of the presence of *V. plantisponsor*; Fig. 6k). Thus, we conclude that the presence of certain bacteria can inhibit *M. vibrans* aggregation. Further work is required to determine whether this has a direct effect on *M. vibrans* or whether it is due to influences of these bacteria on other aggregation-inducing bacteria. Furthermore, more work is needed to determine whether specific molecular factors from bacteria inhibit (or induce) *M. vibrans* aggregation.

## Discussion

Here we demonstrate aggregative multicellularity in the filasterean *M. vibrans*. Taken together with observed cellular aggregation in the distant filasterean *C. owczarzaki*^14^, two choanoflagellates^3,4^ and multiple extant animals^46,47^, we infer that multicellular aggregation was ancestral to Filozoa and present in the unicellular ancestors of animals and their close relatives. Alternatively, each lineage may have evolved this behaviour independently. However, we found that *M. vibrans* expresses many animal multicellularity genes while it forms large stable aggregates. Furthermore, a new analysis of chemically regulated aggregation in *C. owczarzaki* reveals a parallel correlation between aggregation and dynamic multicellular gene expression^48^. In both cases, Holozoa-specific genes that drive cell adhesion, signalling and transcriptional regulation in animal cellular aggregation behaviours are upregulated in aggregates. As such conserved genes are involved in holozoan cellular aggregation, our results suggest that the common ancestor of filastereans and animals had the capacity to use these genes for aggregative multicellularity.

Apart from the filastereans *M. vibrans* and *C. owczarzaki*, many other protists exhibit aggregative behaviours (such as *Dictyostelium*, choanoflagellates and Rhizaria)^3,4,49^. In fact, we suspect that this behaviour is even more common across protists, but the environmental triggers have yet to be reported (as was the case with *M. vibrans* and *C. owczarzaki* until we found the right inducing conditions). The widespread nature of aggregative behaviour suggests that it offers major fitness benefits and supports the hypothesis that the ancestor of animals was capable of aggregation. Several potential benefits have been reported for simple multicellularity^13,50^. Our initial observations with *M. vibrans* suggest that aggregation may improve mating and feeding behaviours; however, both hypotheses require further experimental investigation. We observed genes encoding proteins related to mating (specifically syngamy) and meiosis upregulated during aggregation. Moreover, cell fusion events appeared to occur more frequently under aggregation conditions than in non-aggregation conditions. Thus, like many organisms (including choanoflagellates), *M. vibrans* may leverage aggregation to improve mating efficiency^3^. However, future work is needed to deeply probe this hypothesis, as we have not yet been able to fully control or monitor the putative sexual life cycle of *M. vibrans*. Alternatively, aggregation improved *M. vibrans* replication, and bacteria were trapped within the aggregates. Entrapment of bacteria may increase *M. vibrans* fitness. Initially, the aggregation of cells through a web of interconnected filopodia could improve the capture of bacteria. Subsequently, storing bacteria within the intercellular aggregate space may provide sustained food as the environment changes and bacteria become less abundant. Bacteria might even replicate within the aggregates, providing a constant source of food—although we have no evidence of this currently. The long-term stability of the aggregates is consistent with this hypothesis. The behaviour of harbouring bacteria resembles the primitive ‘farming’ behaviour in *Dictyostelium*, which collects bacteria within its multicellular fruiting bodies^51^. Some choanoflagellates are also known to harbour bacteria within their multicellular clusters^52^. The green alga *Volvox carteri* and the ciliate *Stentor coeruleus* also form multicellular bodies to enhance feeding, therefore accelerating replication^53,54^. Of course, many extant animals have also evolved diverse mechanisms for maintaining microbiomes^55,56^, and microbial cues and interactions have important roles in animal development^57^.

Despite the prevalence of multicellular aggregation across numerous protists and diverse animal cell types, aggregation is rarely considered to be a key trait in the first multicellular animals. A primary concern is the potential for genetic conflict within aggregates of non-clonal cells^11^. Indeed, we found here that non-clonal isolates of *M. vibrans* can co-aggregate. However, recent work has refuted concerns over cheating: spatial structure combined with group selection facilitates the survival of cooperators against cheaters in an aggregate^12^. Moreover, our study demonstrates that *M. vibrans* aggregates are stable once formed, persisting for over 2 months. In other well-studied cases of aggregative multicellularity such as sorocarps, aggregation is a short-lived temporary life stage in response to environmental stress before fruiting-body formation and spore release^58^. Stable aggregation would have allowed more opportunities for complex multicellularity to evolve. We therefore posit that cellular aggregation may have been a key multicellular behaviour that was ancestral to animals and crucial for the evolution of the first animal.

Until now, there was little genetic support for this hypothesis. Namely, there was a lack of clear homology between aggregation-inducing genes in protists and in animals. The best-studied aggregating protists are only distantly related to animals^58,59^. Thus, their aggregation behaviours lack involvement of any Holozoa-specific genes with a role in animal multicellular behaviours. This work combined with a recent study on *C. owczarzaki*^48^ reveals conservation between genes involved in multicellularity of animals and cellular aggregation of their closest relatives. This finding suggests that the ancestor of animals plausibly used these genes for reversible multicellular aggregation. In fact, the most morphologically similar aggregative behaviour in an extant animal is exhibited by sponge cells, which also upregulate genes involved in cell adhesion, integrin and actin remodelling^60^. Furthermore, wound healing responses (which involve the reaggregation of cells and maturation into a tissue) in diverse animals (such as sponges, cnidarians and ctenophores) share transcriptional similarities with *M. vibrans* aggregation^46,47^. The model sponge *Sycon ciliatum* upregulated genes involved in actin filaments and signal transduction during wound healing^61^. The model cnidarian *Nematostella vectensis* also expressed similar adhesion and signalling genes related to cell-substrate adhesion, integrin, GPCRs and receptor tyrosine kinases^46^. Similarly, the ctenophore *Mnemiopsis leidyi* expressed cytoskeletal remodelling and signalling genes related to GPCRs and GTPases^47^. These similar expression profiles of adhesion, cytoskeleton and signalling genes in *M. vibrans* aggregation and animal wound healing support the hypothesis that simple cellular aggregation was a precursor to more complicated cellular reorganization processes in the first animal.

Alternatively, ancestral multicellular aggregation behaviours may have been key to the evolution of animals in more nuanced ways than the first animals directly co-opting the aggregative behaviour of their ancestors. For example, numerous crucial genes for animal multicellular behaviour may have evolved to support the aggregative lifestyle of unicellular animal ancestors. If the ancestors never aggregated, these genes would not have evolved and would not have been available for co-option by the first animals or their early descendants. Thus, either by a direct co-option of aggregation behaviour or by repurposing of aggregation genes, we propose that multicellular animals evolved as a result of ancestral cellular aggregative behaviour.

We cannot exclude the possibility that this large suite of conserved multicellularity-related genes evolved for different behaviour(s) in the ancestor of Filozoa. In that case, filastereans co-opted these genes for their own aggregation lifestyle and choanozoans co-opted them for other behaviours that eventually led to multicellular animals. In this scenario, aggregation may not have been important for the evolution of animals. However, if this were true, we would expect to see these genes correlating with other types of multicellular behaviours in other unicellular relatives of animals, and we do not (at least, not to this degree). For example, choanoflagellate rosettes and ichthyosporean coenocytes have not demonstrated the same massive upregulation of genes associated with animal multicellular behaviours^38,62^. We note that a careful time-series gene expression analysis like ours has not yet been published in these other systems, and such an analysis may reveal parallel expression of these genes correlating with alternative behaviours.

The hypothesis that multicellular aggregation was key to the development of animals does not necessarily exclude the involvement of the other two main types of multicellular behaviour shared between unicellular holozoans and animals (incomplete separation after clonal division and coenocytic development). All three behaviours may have been important in the life cycle of the ancestor of animals, as each behaviour presents a unique advantage to an organism with a complex lifestyle (clonal division avoids cheating, aggregation provides rapid changes and dynamic structures, and coenocytic division yields different cell types)^1^. Alternatively, as stated above, many genes may have initially evolved to support an ancestral aggregative lifestyle, being later co-opted for a different style of multicellular behaviour that was eventually inherited by animals.

In summary, here we identify how a close unicellular relative of animals robustly forms homogeneously large multicellular aggregates. It uses many genes that animals use for cell adhesion, cell signalling and transcriptional regulation throughout its process of aggregation. We therefore hypothesize that the filozoan ancestor of animals used newly acquired multicellularity genes for multicellular aggregation behaviour, perhaps gaining an edge in acquiring food or weathering environmental challenges. The origin of those multicellular genes together with the capacity to form stable aggregates using those genes may have pre-adapted those ancestors to eventually evolve into the first multicellular animals.

## Methods

### Culturing *M. vibrans*

*M. vibrans* Tong (ATCC, 50519; the primary strain in this study) and *M. vibrans* L27 (DSM 114853) were acquired from ATCC or DSM and validated by 18S rDNA amplicon sequencing. They were cultured with 5% seawater complete medium (SWC, 32.9 g l^−1^ tropic marine salt, 5 g l^−1^ peptone, 3 g l^−1^ yeast extract and 3 ml l^−1^ glycerol, pH 8.0) in vented T-25 flasks (Thermo Fisher Scientific, FB012935) at room temperature (23 °C). They were subcultured every 3 days into a fresh T-25 flask by splitting cultures 1:10 with fresh 5% SWC medium.

### 16S rDNA amplicon sequencing

DNA from the *M. vibrans* Tong (ATCC, 50519) and *M. vibrans* L27 (DSM 114853) xenic culture were extracted with the Invitrogen PureLink Microbiome DNA Purification Kit (Thermo Fisher Scientific, A29790). 16S rDNA amplicon sequencing and analysis was performed by Molecular Research LP using bacterial tag-encoded FLX amplicon pyrosequencing-based (bTEFAP) amplicon library preparation for Illumina MiSeq with the primer pair 515F/806R (5′-GTGCCAGCMGCCGCGGTAA-3′, 5′-GGACTACHVGGGTWTCTAAT-3′)^63^. Comprehensive taxonomic analysis, including zero-radius operational taxonomic unit percentages and count compilations at each taxonomic level, were performed by the company Molecular Research LP. Results are provided in Supplementary Data 1 and SRA accessions for all 16S rDNA amplicon sequence reads are provided in Supplementary Data 4.

### Isolation of and 16S rDNA Sanger sequencing of *T. lucentensis* Mv1

Putative *T. lucentensis* colonies were isolated from the *M. vibrans* Tong xenic culture using a SWC agar plate. This isolate was named as *T. lucentensis* Mv1. *T. lucentensis* Mv1 was cultured in 3 ml SWC medium at 30 °C with shaking at 220 rpm overnight. Total genomic DNA was extracted using the Wizard genomic DNA isolation kit (Promega, PAA1120) by using 1 ml of the *T. lucentensis* culture. The 16S rDNA was PCR-amplified using Taq polymerase (BioLabs, M0267L) with primers 16S_F (5′-AGAGTTTGATCCTGGCTCAG-3′) and 16S_R (5′-ACGGCTACCTTGTTACGACTT-3′)^64^. The PCR products were purified with the QIAquick PCR Purification Kit (Qiagen, 28104), and then Sanger sequenced with primer 16S_F by Eurofins Genomics. The resulting sequence is provided in Supplementary Data 2. The sequence was then blasted against NCBI’s nt database by using Nucleotide BLAST^65^.

### *T. lucentensis* whole-genome sequencing

*T. lucentensis* was cultured in SWC medium at 30 °C with shaking at 220 rpm overnight. Then, 1 ml of *T. lucentensis* culture was used for genomic DNA extraction using the Wizard genomic DNA isolation kit (Promega, PAA1120). The genomic DNA of *T. lucentensis* was sent to Eurofins Genomics for whole-genome sequencing. Rapid Barcoding Kit V14 was used for the library preparation. The library was then sequenced with Oxford Nanopore GridION using Kit 14 and R10.4.1 flow cells. The genome was annotated using Bakta (v.1.9.2)^66^. The SRA accession is provided in Supplementary Data 4.

### Monoxenic *M. vibrans* cultures

The *M. vibrans* Tong (ATCC, 50519) xenic culture was treated with an antibiotic cocktail (ofloxacin 10 µg ml^−1^, kanamycin 50 µg ml^−1^, streptomycin 50 µg ml^−1^, ampicillin 50 µg ml^−1^ and chloramphenicol 68 µg ml^−1^) in artificial seawater (ASW, 33 g l^−1^ Tropic Marin Pro Reef Sea Salt, Bulk Reef Supply, 232008, pH 8.0) to kill environmental bacteria. After 48 h, cells were pelleted and resuspended with fresh ASW containing antibiotics for another 48 h. Then, 100 *M. vibrans* cells were sorted by flow cytometry LSRII into wells of a 24-well plate containing 500 µl ASW with antibiotics to avoid bacterial contamination. Cells were maintained in the dark at room temperature overnight. After checking the wells with a microscope (Leica DMIL LED), the wells without visual bacteria were supplemented with 2 ml ASW and 10 µl of *T. lucentensis* Mv1 overnight culture in SWC. Recovered *M. vibrans* cells were transferred to T-25 vented flasks containing 5% SWC after 1 week. DNA from the putatively monoxenic *M. vibrans* culture was extracted using the Invitrogen PureLink Microbiome DNA Purification Kit (Thermo Fisher Scientific, A29790). 16S rDNA amplicon sequencing and analysis was performed by the Center for Genomics and Bioinformatics, Indiana University Bloomington (IUB-CGB). Libraries were prepared using the NEXTFLEX16S V4 Amplicon-Seq Kit 2.0 (with the primer pair 515F/806R described above), and DNA was purified using Agencourt AMPure XP Magnetic Beads. Paired-end sequencing was performed on the MiSeq Nano v2 system (500 cycles). The data were demultiplexed using bcl2fastq, v.2.20.0 (Illumina), and analysed by marker based population analysis (16S rDNA amplicon sequencing). The results are provided in Supplementary Data 3. The *M. vibrans* L27 monoxenic culture (derived from DSM 114853) was also generated with the same protocol as outlined above for the other strain. The culture was then confirmed as monoxenic by 16S rDNA sequencing performed by Molecular Research LP using the same procedure as the *M. vibrans* Tong (ATCC 50519) xenic culture above. The results are provided in Supplementary Data 12 and SRA accessions for all 16S rDNA amplicon sequence reads are provided in Supplementary Data 4.

### Generation and validation of single-eukaryote xenic culture of *M. vibrans* strain L27

The xenic *M. vibrans* L27 culture (DSM 114853) contained two morphologically distinct protists. The culture was serially diluted with 5% SWC medium in wells of a 96-well plate (Thermo Fisher Scientific, 07-200-656) to get mono-eukaryotic cultures. After dilution, cells were cultured at room temperature for 4 days and then checked with a Leica DMIL LED microscope using the ×20 objective. The putative mono-eukaryotic cultures were transferred into T-25 flasks to cultivate for 7 days. One putative mono-eukaryotic culture of each morphology was further examined. Genomic DNA was extracted from each with a Zymo RPI Quick-DNA Miniprep kit (Neta Scientific, RPI-ZD3024) according to the manufacturer’s protocol for cell monolayer samples. In brief, 2 ml of each culture was pelleted at 4,000*g* for 10 min after scraping the flask with a cell scraper (Thermo Fisher Scientific, 50-255-166). The cell pellets were lysed with 500 μl of genomic lysis buffer. Genomic DNA was collected in a Zymo-Spin IICR Column after washing with DNA pre-wash buffer and gDNA wash buffer once. Finally, the total genomic DNA was eluted into a centrifuge tube with 50 µl DNA elution buffer.

The 18S rDNA was PCR-amplified using Phusion Master Mix (New England Biolabs, M0531S) with primers PF1 (5′-GCGCTACCTGGTTGATCCTGCC-3′) and FAD4 (5′-TGATCCTTCTGCAGGTTCACCTAC-3′)^67^. The PCR products were purified using the QIAquick PCR Purification Kit (Qiagen, 28104), and then Sanger sequenced using primer PF1 by Eurofins Genomics. The results are provided in Supplementary Data 11.

Metagenomic 16S rDNA amplicon sequencing was performed on the new single-eukaryote xenic culture as described earlier for the xenic culture. The results are provided in Supplementary Data 13.

### Growth curve of each *M. vibrans* cell line

Each *M. vibrans* cell line was cultured in 5% SWC in a 24-well plate at room temperature. *M. vibrans* cells were counted with a haemocytometer every 24 h after pipetting to disperse possible cell clumps.

### Quantification of aggregation area for each *M. vibrans* cell line

To quantify the average aggregation area, 200 μl of each *M. vibrans* cell line (5.0 × 10^5^ cells per ml) was placed into a well of an ultra-low-attachment 96-well plate (VWR, 29443-034) in fresh 5% SWC for imaging on the Cytation C10 imager (Agilent BioTek) using a ×4 objective every 3 h.

### SEM analysis

For SEM, 500 µl *M. vibrans* cells was placed into poly-l-lysine-coated round coverslips (VWR, BD354085), and the cells left to settle for 1 h. Then, 200 µl of medium was removed, and the cells washed gently with 200 µl of ASW three times. Two fixation steps were applied to the cells. In the first step, the cells were fixed for 30 min using 200 µl of 2% glutaraldehyde in 0.1 M sodium cacodylate buffer, pH 7.4 (Electron Microscopy Sciences, 16536-15), after first washing twice in this solution. In the second step, cells were fixed for 30 min with 1% osmium tetroxide (Sigma-Aldrich, 75632-10 ML). Continuous dehydration was performed by washing the cells twice with an ethanol gradient (30%, 50%, 70%, 80%, 90%, 95% and 100%), soaking the cells in each solution for 10 min before moving to the next. The samples were critical-point-dried in liquid CO_2_ using a samdri-790 critical-point drying apparatus. They were subsequently put on an aluminium mount for Amray 1400 SEM (Electron Microscopy Sciences, 75174) with carbon adhesive tabs (Electron Microscopy Sciences, 77825-12), and sputter-coated with gold-palladium (80:20) with safematic CCU-010. The samples were imaged on the FEI Quanta 600 Scanning Electron Microscope at Indiana University Bloomington.

### TEM

*M. vibrans* cell aggregates in ultra-low-attachment 96-well plates were washed with ASW to remove extraneous material. Wells were filled with 2.5% glutaraldehyde and 2.0% osmium tetroxide in 0.1 M sodium cacodylate buffer, pH 7.2 (>200 µl) on ice after removing ASW. Cells were then washed with 0.1 M sodium cacodylate buffer (pH 7.2) four times and then embedded with 1% agarose in a 0.1 M sodium cacodylate buffer. Cells in agarose plugs were dehydrated with a graded ethanol series of 30%, 50% and 75% (each containing 2% uranyl acetate), followed by 90%, 95% and 100% (lacking uranyl acetate) for 10 min each. The ethanol solutions were kept on ice. The samples were placed into a 1:1 mixture of ethanol and acetonitrile for 1 h and then in 100% acetonitrile for another hour. The samples were placed in a mixture of acetonitrile and Embed 812 (Electron Microscopy Sciences, 14130) at the ratios of 3:1, 1:1 and 1:3 for 2 h at each dilution, and then in 100% Embed 812 three times, one time overnight and the other two times for a minimum of 2 h. The resin-embedded sample was then polymerized at 65 °C for 18 h. The resin blocks were sectioned at 70 nm using a Leica Ultracut UCT ultramicrotome. Ultrathin sections, stained with uranyl acetate and lead citrate, were imaged with a JEOL JEM 1010 transmission electron microscope at 80 kV using a Gatan 794 camera at Indiana University Bloomington.

### Blocking replication of *M. vibrans*

To block replication, 2 ml of *M. vibrans* cells (1.0 × 10^5^ cells per ml) in fresh 5% SWC were placed in a 24-well plate and treated with 20 or 40 μg ml^−1^ aphidicolin (Sigma-Aldrich, 178273-1MG) or DMSO. Cell density was counted every 24 h with a haemocytometer for 6 days to determine the concentration of aphidicolin that can block cell replication. Once the 20 µg ml^−1^ condition was established, 200 µl of 20 μg ml^−1^ aphidicolin- or DMSO-treated *M. vibrans* cells (5.0 × 10^5^ cells per ml) were placed into an ultra-low-attachment 96-well plate for imaging with a Cytation C10 imager every 2 h for 3 days.

### Staining *T. lucentensis* in the aggregates of *M. vibrans*

5-Cyano-2,3-di-(*p*-tolyl)tetrazolium chloride (CTC, Sigma-Aldrich, 94498-10MG-F) was used to stain the bacterium *T. lucentensis* within the aggregates of *M. vibrans*. CTC was dissolved in ASW to a 1.2 mM concentration. A 1-day old *M. vibrans*–*T. lucentensis* culture (cell density, 1.0 × 10^5^ cells per ml) was pelleted (4,000*g* for 10 min at room temperature) and resuspended in this CTC working solution and 200 μl added to wells in a 96-well plate (Thermo Fisher Scientific, 08-772-225). The cells were then cultured in the dark at room temperature for 24 h. The cells were washed with 100 μl fresh medium gently (10 μl of media ten times) before imaging on the Leica Stellaris 8 confocal microscope using a ×63 objective. Images were then processed with ImageJ^68^.

### Plasma membrane staining of *M. vibrans*

Eukaryotic cytoplasmic membrane staining dyes BioTracker 490 Green and 555 Orange (Sigma-Aldrich, SCT106 and SCT107, respectively) were used to stain the membrane of both monoxenic *M. vibrans* Tong and L27 cultures. The assay protocol was slightly modified from the manufacturer’s recommendation. In brief, 1-day-old *M. vibrans*–*T. lucentensis* monoxenic cultures (cell density, 5.0 × 10^5^ cells per ml) were pelleted at 4,000*g* for 10 min at room temperature. After removing the supernatants, cells were suspended in membrane staining working solutions (4 μl of the stain stock per 1 ml in ASW). The cells were incubated for 1 h in the dark at room temperature. Cells were then washed four times with ASW by pelleting at 4,000*g* for 10 min at room temperature. Then, 100 μl of each of the two stained cultures was mixed together in wells of a 96-well plate (Thermo Fisher Scientific, 08-772-225), with 200 μl of each individually stained culture used as controls. Cells were cultured in the dark at room temperature for 24 h before imaging on the Leica Stellaris 8 confocal microscope with a ×63 objective for quantification. For the 3D video of double-stained cells, 60 μl of cells was transferred onto a glass coverslip (VWR, 48366277), and the cells were left to settle for 1 h in the dark. The medium was removed by gentle pipetting. Cells were then mounted with ProLong gold antifade reagent (Thermo Fisher Scientific, P36934) on a plain slide (VWR, 48300026) and left in the dark for 1 h. Images were taken on the OMX super-resolutiosn microscope with a ×60 objective and then were processed with ImageJ^68^.

### Visualization of *M. vibrans* cell fusion

To record cell fusion of *M. vibrans*, cells were subcultured with fresh medium (1:5, v/v) in a new vented T-25 culture flask at room temperature for 2 days. A Leica LMi1 microscope with a ×40 objective was used to record cell fusion events by focusing on the cells around the edges of *M. vibrans* aggregates.

### RNA-seq analysis

Total RNA was extracted using a protocol modified from a previous study^14^. In brief, 1.0 × 10^7^ cells were collected into a 15 ml Falcon centrifuge tube after incubating in ultra-low-attachment plates in a Cytation C10 imager at 26 °C for 12, 18, 24, 33, 48 and 72 h (4 replicates each). The supernatants were removed by pelleting at 4,000*g* for 5 min. Cells were lysed with 1 ml Trizol for 5 min and 200 µl 1-bromo-3-chloropropane for 15 min at room temperature. Total RNA and DNA were then precipitated with isopropanol and rinsed with freshly-prepared 75% ethanol. The pellet was redissolved in 50 µl of 1× DNase I buffer, and DNA digested using DNase I (Thermo Fisher Scientific, EN0521) while inhibiting RNases using RNAseOUT (Thermo Fisher Scientific, 10777019). RNA was purified by precipitation with 5 µl LiCl (5 M) in 2× total volume (110 µl) 100% ethanol (previously cooled to −20 °C). RNA was washed with 70% ethanol (previously cooled to −20 °C) and dissolved in 20 µl of nuclease-free water. The samples were sent to Novogene for mRNA library preparation (poly(A) enrichment) using poly(T) oligo-attached magnetic beads. Quantified libraries were sequenced on an Illumina NovaSeq X Plus system using 150 bp paired-end (PE150) chemistry. SRA accessions for RNA-sequence reads are provided in Supplementary Data 4.

### *M. vibrans* functional annotation

All analyses were performed using the recently published *M. vibrans* Tong (ATCC 50519) genome^14^. Genomic data files available from the Figshare^69^ repository associated with the publication were used for all analyses, specifically the files Mvib.PASAupdated_BRAKERproteins.070617.fa, Mvib.PASAupdated_BRAKERproteins.070617.gff for protein sequences and gene annotations and the file Mvib_cleangenome.withMito18S.plusaddedfromdraft.300417.fa for nucleotide sequences. Genes were functionally annotated using several different tools and databases. Emapper (v.2.1.9)^70^ using eggNOG (v.5.0.0)^71^ with the settings ‘-m hmmer --tax_scope auto --tax_scope_mode inner_narrowest --go_evidence all --pfam_realign denovo’ at both the Eukaryota and Metazoa levels. InterProScan (v.5.62-94.0)^72^ was used with the settings ‘-appl Pfam, TIGRFAM, PANTHER, -iprlookup, -pa’. Top hits were identified using BLAST+ (v.2.14.1+)^73^ BLASTp with the settings ‘-max_target_seqs 5, -max_hsps 1’ against the *C. owczarzaki* genome (GCF_000151315.2), *Homo sapiens* GRCh38 genome (GCF_000001405.40) and EukProt V3 TCS dataset^74^. DIAMOND (v.2.1.9)^75^ with the options ‘--max-target-seqs 5, --more-sensitive’ against Swiss-Prot^76^, and NCBI’s RefSeq and NR databases^77^. Hits with *e* values greater than 1 × 10^–5^ were removed. BlastKOALA (v.3.0)^78^ was applied with the KEGG GENES database ‘Eukaryotes’. Functional annotations were combined into an overview across genes using a custom script^79^ (make_Mvib_annotations_table.py; Supplementary Data 5).

### Processing of RNA-seq reads

RNA-sequencing (RNA-seq) reads were processed and mapped against the *M. vibrans* genome using the nf-core/rnaseq pipeline (v.3.14.0)^80–82^ with the options --igenomes_ignore --genome null --skip_bbsplit false --remove_ribo_rna --aligner star_rsem --stringtie_ignore_gtf --bam_csi_index -profile singularity. Specifically, adaptors were removed and reads trimmed using Trim Galore!^83^ with read quality control checked using FastQC^84^. Reads that mapped against the *H. sapiens* GRCh38 genome and *T. lucentensis* Mv1 genome were removed using BBSplit from BBMap^85^ and ribosomal RNA removed using SortMeRNA^86^. Reads were then aligned to the *M. vibrans* genome using STAR^87^ and quantified using RSEM^88^. The resulting gene quantification files were used for downstream analyses, and read counts are available in Supplementary Data 6.

### Sample relationships analyses

Gene quantification files were imported into RStudio (v.2023.12.1+402)^89^ with R (v.4.3.3)^90^ using tximport (v.1.30.0)^91^ and loaded into a DESeq dataset using DESeq2 (v.1.42.1)^92^. Genes with low counts, fewer than ten counts in less than four samples, were removed and counts from the remaining genes (10,852 of 12,127 genes) normalized using estimateSizeFactors to allow for between-sample comparisons (Supplementary Data 6). The resulting normalized counts were then subjected to a variance-stabilizing (vsd) transformation to remove dependence of the variance on the mean using vsn^93^ (v.3.70.0; Supplementary Data 6). A sample distance matrix of the vsd transformed data were then calculated using ‘dist’ and sample relationships visualized through a principal component analysis plot using plotPCA and a heat map of sample to sample distances using pheatmap (v.1.0.12)^94^.

### Identification and clustering of DEGs

DEGs that differed significantly in gene expression across the time series were then identified using the likelihood ratio test (LRT) with the options ‘alpha = 0.0000000001 and filterFun=ihw’ using IHW (v.1.30.0)^95^ and DESeq2 (v.1.42.1)^92^. Genes with adjusted *P* < 1 × 10^−10^ were retained for downstream analyses (6,472 genes; Supplementary Data 7). DEG clusters with a minimum size of 250 genes were then identified using degPatterns from DEGreport (v.1.38.5)^96^. This resulted in eight overarching DEG clusters that captured the most common gene expression profiles across the time series (Supplementary Data 7), and which were visualized with pheatmap (v.1.0.12)^94^.

### Functional enrichment analysis

Significantly enriched GO terms and KEGG pathways in each DEG cluster were then identified using enricher from clusterProfiler (v.4.10.1)^97^ with the default settings (*P* ≤ 0.05) (Extended Data Fig. 8). An organism database including GO terms annotated from across various tools was compiled using AnnotationForge (v.1.44.0)^98^. For GO term enrichment analysis, a topGO data object for Biological Processes GO terms was then compiled and significance tested using runTest with the options ‘algorithm = ‘weight01’, statistic = ‘fisher’’ from TopGO (v.2.54.0)^99^. Significantly enriched GO terms (weighted Fisher ≤ 0.001; Supplementary Data 8) were retained and calculateSimMatrix from GOSemSim (v.2.28.1)^100^ was used to calculate the semantic score similarity between terms with the settings ‘ont = ‘BP’, method = ’Re’ and keytype = ‘GID’’. Terms were then reduced using reduceSimMatrix with a threshold of 0.9. Resulting GO terms and similarity data were then plotted using Rrvgo (v.1.14.2)^101^. R analyses of RNA-sequencing data are available in an R markdown file^79^.

### Annotation and analyses of genes of interest

Genes encoding homologues of proteins involved in animal multicellularity^102–104^ and previously investigated in unicellular holozoans were identified in *M. vibrans* using compiled functional annotation data. Specifically, the presence of Interpro^105^ and Pfam^106^ protein domains and, in some cases, domain structure and the presence of specific motifs, PANTHER^107^ and KEGG KO^108^ assignments, reciprocal best hits against human homologues using BLAST+ (v.2.14.1+)^73^, top hits to *C. owczarzaki* homologues^109^ and genes previously reported in *M. vibrans*^17,20,33,110–116^ (Supplementary Data 9). Previously investigated genes encoding phagocytosis-related proteins^20,117^ and meiosis- and syngamy-related proteins^45,118,119^ were identified on the basis of the presence of conserved protein domains and functional annotations (Supplementary Data 10). The differential expression of genes encoding meiosis and syngamy related proteins was also investigated at higher significance cut-offs. Individual gene expression profiles were visualized in heat maps using pheatmap (v.1.0.12)^94^.

### Gene age inference

Gene ages for key multicellularity proteins were inferred using several different strategies. First, gene family age based on originations from a recent ancestral state reconstruction of Opisthokonta genome evolution were collected^14^. Second, gene age was inferred using GeneBridge (v.0.99.2)^120^. Here, the species topology and gene families from a previous study^14^ were used and the tree was uploaded using ape (v.5.8.1)^121^. GeneBridge was run with a threshold of 0.3 and 100,000 permutations. Third, results from previous studies investigating the evolutionary history and distribution of genes encoding these proteins and domains were compiled^17,20,22,24,26,30,32,34,109,111–113,116,122–126^. A consensus gene age was then selected for visualizations, with inferences from previous studies taking precedence as they were often the most conservative (Supplementary Data 9). Where relevant, focus was placed on the inferred gene age of the larger protein family/domain rather than specific paralogues.

### Data visualization

For various R analyses, data were formatted using tidyverse (v.2.0.0)^127^, dplyr (v.1.1.4)^128^ and plyr (v.1.8.9)^129^, with additional plots visualized using ggplot2 (v.3.5.2)^130^ and cowplot (v1.1.3)^131^, together with colour palettes from RcolorBrewer^132^ and viridis (v.0.6.5)^133^.

### Reporting summary

Further information on research design is available in the Nature Portfolio Reporting Summary linked to this article.

## Online content

Any methods, additional references, Nature Portfolio reporting summaries, source data, extended data, supplementary information, acknowledgements, peer review information; details of author contributions and competing interests; and statements of data and code availability are available at 10.1038/s41586-026-10748-5.

## Supplementary information

Supplementary Information Supplementary Discussion 1–3, Supplementary Figs. 1–6, descriptions of Supplementary Data 1–13 and Supplementary References.

Reporting Summary

Supplementary Data Supplementary Data 1–13.

Peer Review File

Supplementary Video 1 Aggregation of monoxenic *M. vibrans* Tong (ATCC 50519) with *T. lucentensis* Mv1 over 72 h.

Supplementary Video 2 Aggregation of aphidicolin-treated monoxenic *M. vibrans* Tong (ATCC 50519) with *T. lucentensis* Mv1 over 36 h.

Supplementary Video 3 Aggregation of monoxenic *M. vibrans* Tong (ATCC 50519) with *T. lucentensis* over 1,140 h (60 days).

Supplementary Video 4 3D image stacks of a double-stained *M. vibrans* cell.

Supplementary Video 5 Time-lapse video of *M. vibrans* cell fusion event by focusing on the surrounding of aggregates.

Supplementary Video 6 Initial xenic *M. vibrans* L27 culture (DSM 114853) grown in 5% SWC. Two different eukaryotic cells are present. Larger cells that are generally solitary and occasionally swimming were identified by 18S amplicon sequencing to be *Paraphysomonas imperforate*. Smaller cells that are generally in multicellular clusters were identified by 18S amplicon sequencing to be *M. vibrans*.

Supplementary Video 7 Aggregation of xenic *M. vibrans* L27 (DSM 114853) over 72 h.

Supplementary Video 8 Aggregation of monoxenic *M. vibrans* L27 (DSM 114853) with bacterium *T. lucentensis* Mv1 over 72 h.

## Data Availability

Sequencing reads have been deposited at the NCBI SRA database under BioProject PRJNA1229532 from RNA-seqg (SRR32514592 to SRR32514615), 16S rDNA amplicon sequencing (SRR33469057 to SRR33469059 and SRR34719544) and the *T. lucentensi*s strain Mv1 (SRR33469287). Information about which SRA accession corresponds to which sample alongside BioSample accessions can be found in Supplementary Data 4. The assembled genome for the *T. lucentensis* strain Mv1 was also deposited at GenBank (GCA_050516545.1) under the same BioProject accession. Output from annotation tools, results files from the nf-core/rnaseq pipeline and an R markdown script and html file for re-running R analyses is provided at the Figshare data repository^79^ (10.6084/m9.figshare.29027924). The following additional datasets and databases were also used (with associated accession numbers and links provided): *H. sapiens* GRCh38 genome (GCF_000001405.40), *C. owczarzaki* genome v2 (GCF_000151315.2), *M. vibrans* v1 genome (GCA_943704425.1), EukProt V3 TCS dataset (https://evocellbio.com/eukprot), NCBI’s RefSeq and NR databases (https://www.ncbi.nlm.nih.gov/refseq/), KEGG GENES database (https://www.genome.jp/kegg/genes.html), eggNOG (http://eggnog5.embl.de), InterPro (https://www.ebi.ac.uk/interpro/), UniProt/SwissProt (https://www.uniprot.org/), EukProt (https://evocellbio.com/eukprot/) and NR (https://www.ncbi.nlm.nih.gov/protein/).

## Code availability

The custom script make_Mvib_annotations_table.py for processing the functional annotation output into the overview presented in Supplementary Data 5 is provided at Figshare^79^ (10.6084/m9.figshare.29027924).
