# Supplementary material for: A unicellular relative links aggregative multicellularity to animal origins
Source: Nature. 2026 Jun 9;656(8129):913–23. doi: 10.1038/s41586-026-10748-5 (PMC13366479; doi:10.1038/s41586-026-10748-5)
Supplement: Supplementary file 1 [file 41586_2026_10748_MOESM1_ESM.pdf]

nature portfolio

https://doi.org/10.1038/s41586-026-10748-5

Supplementary information

A unicellular relative links aggregative multicellularity to animal origins

In the format provided by the authors and unedited

SUPPLEMENTARY INFORMATION

A unicellular relative links aggregative multicellularity to animal origins

Ruibao Li (黎瑞宝) [1,5], Jennah E. Dharamshi[2,5], Kyle Kwok[1], Iñaki Ruiz-Trillo[3,4]*, Joseph P. Gerdt[1]*

[1] Department of Chemistry, Indiana University Bloomington, Indiana, USA

[2] Department of Organismal Biology, Systematic Biology, Uppsala University, Uppsala, Sweden

[3] Institut de Biologia Evolutiva (CSIC-Universitat Pompeu Fabra), Barcelona, Spain

[4] ICREA, Pg. Lluís Companys 23, 08010 Barcelona, Spain.

[5] These authors contributed equally to this work

*Corresponding authors, email: inaki.ruiz@ibe.upf-csic.es, jpgerdt@iu.edu

SUPPLEMENTARY DISCUSSION

Supplementary Discussion 1 – Enrichment analyses of differentially expressed gene clusters

To identify functions of importance for *M. vibrans* aggregation, we examined enriched Gene Ontology (GO) terms, Kyoto Encyclopedia of Genes and Genomes (KEGG) pathways, and Pfam protein domains across the eight differentially expressed gene (DEG) clusters. When considering the four DEG clusters composed of net upregulated genes (Fig. 4, Extended Data Figs. 6 and 8, Fig. S1, and Data S7–8), we found that functions related to DNA replication (e.g., ko03030), nucleotide metabolism (e.g., ko00240), mRNA splicing (e.g., GO:0000398), and the initiation of cell division (e.g., GO:0010389), were enriched among genes with peak expression early at 24 h in the *M. vibrans* aggregation process (early peak DEG cluster), and which subsequently drop in expression close to initial levels. Soon afterwards, functions related to the cell cycle (e.g., ko04110 and ko04111), kinetochore (eight GO terms), and mitosis (20 GO terms) reached peak gene expression at 33 h (middle peak DEG cluster) and remained elevated in comparison to initial expression levels. Unexpectedly, we found that eight GO terms related to meiosis were also enriched, suggesting that a sexual life stage may be occurring in *M. vibrans* aggregates. GO terms related to programmed cell death (GO:0035096) and cell localization and migration (five GO terms) were only later enriched among genes with peak expression in large mature aggregates at 72 h (late peak DEG cluster). The largest group of upregulated genes (constantly increasing DEG cluster) continued to increase in gene expression throughout the *M. vibrans* aggregation process. Strikingly, many of the enriched GO terms were associated with multicellularity and development, including cell adhesion (five GO terms) and cell signalling (35 GO terms), such as heterophilic cell–cell adhesion (GO:0007157), the G protein-coupled receptor (GPCR) signalling pathway (GO:0007186), regulation of Rho protein signal transduction (GO:0035023), MAPK cascade (GO:0000165), and positive regulation of hippo signalling (GO:0035332). Mirroring this, Pfam protein domains associated with both cell adhesion and signalling were also enriched and include epidermal growth factor-like domains (PF00008, PF07645, and PF12661), sushi repeats (PF00084), secretin 7 transmembrane receptors (PF00002),

and G protein-coupled receptor proteolysis sites (PF01825). GO terms related to meiosis, signaling involved in phagocytosis, negative regulation of immunity, and chemotaxis and cell localization were also enriched, in addition to KEGG pathways involving starch and glycan degradation (e.g., ko00500, ko00531, and ko00511).

Among the DEG clusters composed of net downregulated genes (Fig. 4, Extended Data Figs. 7–8, Fig. S2, and Data S7–8), we found that one group (late dip DEG cluster) initially underwent a small increase in gene expression with a peak at 18 h before dropping in expression after 24 h. The functions enriched among these genes suggest an initial increase in transcription (i.e., RNA polymerase II preinitiation complex assembly, RNA export from nucleus, and mRNA processing), ribosome biogenesis (e.g., ko03008 and ko03009), translation (four GO terms), rRNA processing (ten GO terms), and the proteasome (i.e., ko03050), suggesting protein turnover. Another group of genes (early dip DEG cluster) underwent an initial decrease in gene expression at 24 h before increasing again, although not to initial expression levels. Here, enriched functions include autophagy (i.e., ko04140 and GO:0016236), programmed cell death (GO:0035096), and negative regulation of cellular response to oxidative stress (GO:1900408). Genes in the middle dip DEG cluster decreased in gene expression, reaching a dip at 33 h and remaining downregulated. Functions enriched in this group include stress responses such as oxygen-glucose deprivation (GO:0090650) and hypoxia (GO:1990910), and various catabolic and biosynthetic processes including tryptophan (GO:0006569), and amine (GO:0042402) catabolism, and tyrosine (GO:0006571), carnosine (GO:0035499), and phosphatidylethanolamine (GO:0006646) biosynthesis. The largest group of downregulated genes (constantly decreasing DEG cluster) continued to decrease in gene expression throughout the *M. vibrans* aggregation process and was enriched in many functions associated with central metabolism, including glycolysis/gluconeogenesis (ko00010), the TCA cycle (ko00020), and oxidative phosphorylation (ko00190). Mitochondrial biogenesis (ko03029) and mitochondrial import and electron transport (17 GO terms) were also particularly prevalent among enriched functions.

Supplementary Discussion 2 – Animal multicellularity-related genes are expressed during *M. vibrans* aggregation

Stable and organized adhesion between neighboring cells is a critical requirement for the evolution of complex multicellularity. Extant animals achieve this through the deployment of genes encoding proteins for structural remodeling and cellular scaffolding, such as those involved in actin cytoskeleton organization, producing extracellular matrix (ECM) to form a basement membrane for cell and tissue support, and complexes that mediate cell–ECM and cell–cell adhesion. An extensive repertoire of such cell adhesion genes is also found encoded in the genomes of unicellular holozoans, although in many cases we do not know their function in this context.[1-16] We found that the large majority of *M. vibrans* cell adhesion DEGs were upregulated (Fig. 5, Extended Data Fig. 9, Fig. S3, and Data S9). These included genes encoding the actin filament bundling proteins spectrin (SPT), fascin (FSCN), and diaphanous (DIA) inferred to have originated by the urfilozoan ancestor, and myosin X (MYOX) inferred to have originated by the urholozoan ancestor.[4,5,10,16] SPT is a cytoskeletal protein necessary for resisting mechanical stress and forming epithelia in animals, it has previously been described in choanoflagellates and its protein domains are also found in *C. owczarzaki*.[1,8,10,17] FSCN is involved in animal filopodia and microvilli actin cytoskeleton organization, with the *S. rosetta* homolog localizing to analogous structures and *C. owczarzaki* homologs expressed in adherent cells.[4] DIA functions in actin filament nucleation, and MYOX is essential for filopodia formation in animals.[4,5] Diverse gene homologs of ECM proteins were also upregulated during *M. vibrans* aggregation, including a previously identified putative alpha type IV collagen (COL4, PF01413), which is the only case found thus far outside Metazoa (Fig. 5b–d).[12] In addition, genes encoding laminin G (LAM G, PF02210), fibronectin type II and III (FN I, PF00040 and FN III, PF00041), and the fibrinogen C-terminal (FG C, PF00147) protein domains were upregulated, having a peak in expression either during aggregate formation or in large mature aggregates (Fig. 5c). We also found that a number of genes encoding thrombospondin (TSP) type 1 protein domains (PF00090) were upregulated (Extended Data Fig. 9). However, none encoded the thrombospondin C-terminal protein domain (PF05735) found in some choanoflagellates.[13] Genes encoding several of these ECM protein domains have previously been predicted to be secreted by the

choanoflagellate *Monosiga brevicollis* and filasterean *C. owczarzaki*.[9] In terms of cell–ECM adhesion, we identified several dystroglycan complex gene homologs in *M. vibrans*, including genes encoding putative syntrophin, dysbindin, dystrophin, and sarcoglycan proteins (Data S9). However, none were differentially expressed across the time series. On the other hand, gene homologs of most integrin adhesome proteins were upregulated. The integrin adhesome plays a central role in cell–ECM adhesion in animals and has ancient origins in the last common ancestor of Amorphea.[3,18,19] Homologs of integrin alpha (ITGA), integrin beta (ITGB), talin (TLN), and integrin-linked kinase (ILK) were all upregulated, although PINCH and parvin (PARV) were not significant DEGs (Fig. 5c and Data S9). In addition, we found that homologs of tyrosine-protein kinase c-Src (SRC), SRC-like, and C-terminal Src kinase like (CSK-like) were all upregulated. These function in or are closely related to genes that function in integrin-mediated signalling and are thus far only found in Holozoa.[11] The alpha-catenin/vinculin family (VIN, PF01044) includes both vinculin and alpha-catenin, which function in the integrin adhesome and cell–cell adhesion, respectively and cannot be distinguished in non-metazoans.[7] Mirroring *C. owczarzaki*, we found that *M. vibrans* also encodes three VIN homologs, two of which are upregulated during aggregation. *M. vibrans* encodes several additional genes involved in cell–cell adhesion in animals, including genes encoding cadherin (CDH) domains, C-type lectin (CLEC) domains, and ezrin-radixin-moesin-merlin (ERM-merlin) family proteins. Holozoan-type CDH domains have a urfilozoan origin and *C. owczarzaki* encodes only one CDH, while *M. vibrans* encodes eight (PF00028). This number is on the same order as ctenophores, placozoans, and some sponges, but it is fewer than the 23 and 29 found in *M. brevicollis* and *S. rosetta*, respectively.[2,6] Four *M. vibrans* CDHs are DEGs, and all are upregulated, including two genes (g1326 and g10477) that increased over 15 times in gene expression over the time series (Fig. 5b–d and Data S9). Canonical CLEC domains (PF00059) have a patchy distribution in Holozoa and were previously reported to be absent in Filasterea.[15] However, we found that *M. vibrans* encodes two genes with the domain and a third with a different CLEC domain (PF19439). One of the former (g5462) is a DEG and upregulated during aggregation. The ERM-merlin gene family is thus far only found in holozoans and includes both ERM cytoskeletal proteins involved in forming cell–cell contacts and merlin, a gene in the Hippo signalling pathway, which cannot be

clearly distinguished between in unicellular Holozoa.[14] Of the four *M. vibrans* ERM-merlin homologs, one was a DEG with a peak in gene expression at 33 h (Fig. 5c and Data S9).

Cell signalling mechanisms involved in intracellular signal transduction, immunity, environmental signal/response pathways, and cell–cell communication are also critical for evolving complex multicellularity. Animal multicellular development is mediated by a small set of specific cell signalling pathways and receptors (*e.g.*, Hippo, Hedgehog, Wnt, TGF-beta, Notch/Delta, toll-like receptors (TLRs), teneurins, membrane-associated guanylate kinases (MAGUKs), nuclear hormone receptors (NR), Ephrin-Eph, and JAK-STAT) alongside eukaryote-wide cell signalling mechanisms that have undergone gene expansions and/or subfunctionalization (e.g., protein-tyrosine kinases (TKs), receptor-tyrosine kinases (RTKs), mitogen-activated protein kinases (MAPKs), phosphatidylinositol 3-kinases (PI3Ks), small GTPases and regulators, calcium signalling, and G-protein coupled receptors (GPCRs)).[20-29] Gene homologs and protein domains from several specific cell signalling pathways and receptors have now been identified outside of Metazoa, primarily in unicellular Holozoa, including Hippo, Hedgehog, Notch/Delta, MAGUKs, TLRs, and teneurins.[1,6,8,23,30] We identified many gene homologs of cell signalling proteins in the *M. vibrans* genome, with the majority of DEGs upregulated across the aggregation time series (Fig. 5, Extended Data Fig. 9, Fig. S3, and Data S9).

In terms of signalling regulators, we found 101 predicted TK and serine/threonine kinases (STKs) (PF07714). Of these, 48 have the TK catalytic motif, correspond to previously described *M. vibrans* TKs, or are top hits to them;[21] nearly all of which were upregulated (Extended Data Fig. 9 and Data S9). Further analyses are necessary to determine whether the remaining genes represent TKs or STKs. In addition, *M. vibrans* encodes seven putative MAPKs, three of which had an early peak in gene expression (Fig. 5c and Data S9). We also identified five PI3Ks, with three upregulated, while the downstream mTOR decreased in expression in large mature aggregates. In animals, MAGUKs regulate cell–cell adhesion, they were once thought to be Metazoa-specific but have also been found in choanoflagellates and *C. owczarzaki*.[1,8,27] We identified four partial MAGUK homologs, of which one was upregulated, a second peaked in expression during

aggregation, and a third (a putative disc large MAGUK (DLG)) dropped in expression in large mature aggregates. Small GTPases and their regulators are key signalling components in central cellular processes in eukaryotes where they act as molecular switches in response to various signals including from receptors.[31] We annotated 81 Small GTPases (PF00071), of which eight are putative Ras GTPases (IPR020849) and ten are putative Rho GTPases (IPR003578). We also annotated 47 guanine nucleotide exchange factors (RhoGEF, PF00621; RasGEF, PF00617) that activate GTPases, 44 GTPase-activating proteins (RhoGAP, PF00620; RhoGAP, PF00620) that negatively regulate GTPases, and three guanine nucleotide dissociation inhibitors (RhoGDI, PF02115) that sequester GTPases (Data S9). Expression patterns among the small GTPases were more variable, but the majority of RhoGEFs and RhoGAPs were upregulated, while RhoGDIs were downregulated (Extended Data Fig. 9). In particular, we found that the RhoGEF VAV, which has only been described in filozoans,[32] was upregulated (Fig. 5b–c).

Regarding specific signalling pathways, we found homologs of both Ca2+/calmodulin-dependent protein kinase (CAMK) and inositol 1,4,5-trisphosphate receptor/ryanodine receptors (IP_3R/RyRs), which are involved in calcium signalling with the later previously investigated in *M. vibrans*.[29] CAMK had a middle peak in gene expression, and all IP_3Rs that were differentially expressed and the one RyR homolog were upregulated (Extended Data Fig. 9). In animals, the Hippo pathway regulates cell proliferation, apoptosis, and tissue size, with many components having pre-metazoan origins.[33] In the *M. vibrans* genome we identified homologs of Yes-associated protein 1/Yorkie (YAP1/yki), WW and C2 domain containing/kidney and brain expressed protein (WWC/Kibra), Lethal (2) giant larvae (LLGL/Lgl), atypical protein kinase C (aPKC), Large tumor suppressor kinase/Warts (LATS/Wts), TEA domain transcription factor/scalloped (TEAD/Sd), Macrophage stimulating 1/Hippo (MST1/Hpo), and MOB kinase activator 1/mob as tumor suppressor (MOB1/Mats) (Data S9). Of these, homologs of YAP1/yki, WWC/KIBRA, LLGL/Lgl, and aPKC, thus far only described in holozoans and filozoans,[33] were differentially expressed) alongside TEAD/Sd and MOB1/Mats (Figure 5b–d). In *C. owczarzaki*, YAP1/yki regulates the shape of multicellular aggregates,[34] and its

upregulation during *M. vibrans* aggregation could suggest a similar role. Most components of the Hedgehog pathway are animal-specific where the pathway is central in regulating cell differentiation.[23] However, choanoflagellates encode genes with either hedge (*i.e.*, hedgling) or hint protein domains that together are found in hedgehog (HH/hh), and homologs of the hedgehog receptor patched also found in other eukaryotes.[1,6,30,35-38] Unexpectedly, we identified six *M. vibrans* genes encoding proteins with a hint protein domain (PF01079), alongside four genes encoding proteins with a patched protein domain (PF02460) (Data S9). Nearly all were differentially expressed, with two hint domain-containing genes increasing in expression and two patched domain-containing genes sharply increasing in expression at 18 h (Fig. 5c). With the exception of Glycogen synthase kinase 3 (GSK-3) from the Wnt pathway, and several TGF-β related protein domains found in choanoflagellates, the Wnt, TGF-β, NR, and JAK pathways and receptors remain animal-specific,[23,39] with no homologs found in *M. vibrans*.

In terms of signalling receptors, we found that *M. vibrans* encodes an extensive repertoire of GPCRs: one Rhodopsin/Class A (7tm1, PF00001) GPCR, 51 Adhesion/Secretin/Class B (7tm2, PF00002) GPCRs, six Glutamate/Class C (7tm3, PF00003) GPCRs, two cAMP/ClassE (Dicty_CAR, PF05462) GPCRs, one Abscicic acid receptor (ABA_GPCR, PF12430) GPCR, and three GOST seven transmembrane (GOST_TM, PF06814) GPCRs (Data S9). *M. vibrans* encodes an exceptional number of 7tm2 GPCRs, on the same order as metazoans[22] and more than choanoflagellates.[40] The vast majority of those that were differentially expressed were found to be upregulated during aggregation (Fig. 5c). Most 7tm3 GPCRs were also upregulated, alongside the ABA_GPCR GPCR and one Dicty_CAR GPCR, while 7tm1 and GOST_TM GPCRs were downregulated. Notch proteins from the Notch/Delta signaling pathway have previously been described in Filozoa.[8,38,39] We identified three Notch domain-containing proteins (PF00066) in the *M. vibrans* genome, two of which encode the Notch domain followed by Ankyrin repeats, and thus have moderate evidence as Notch proteins. All three were upregulated alongside a putative recombining binding protein suppressor of hairless (RBPJ) gene, which is a TF in the pathway.

Transcriptional regulation through the activity of TFs is crucial for coordinating gene deployment in animal development. The TF repertoires of complex multicellular lineages are likewise more complex, although they were assembled in a stepwise manner and many key animal TF families have pre-metazoan origins. [41-45] In the *M. vibrans* genome we identified a large repertoire of TFs important in Metazoa: thirteen basic helix loop helix (bHLH, PF00010) TFs including Myc-Max network homologs (MAX, MNT/Mxd, MYC), four basic leucine zipper (bZIP, PF00170) TFs, seven high mobility group box (HMG, PF00505) TFs including a putative SRY-related like (SOX-like) gene homolog, two MADS-box (PF00319) TFs including a putative Myocyte-specific enhancer factor 2 (MEF2) gene homolog, six homeodomain (HD, PF00046) TFs, two TALE-related HD (PF05920) TFs, eight forkhead (FOX, PF00250) TFs , one CP2 (PF04516) TF that is grainyhead-like (GRH), one Churchill (PF06573) TF, two T-box (PF00907) TFs, specifically Brachyury and Tbx7, three Runt-related (RUNX, PF00853) TFs, one p53 (PF00870) TF, one Rel homology DNA-binding (RHD, PF16179) TF, and two regulatory factor X (RFX, PF02257) TFs (Data S9). In contrast to cell adhesion and signaling, a nearly equal number of transcriptional regulation genes that were differentially expressed were net upregulated and downregulated across the *M. vibrans* aggregation time series (Fig. 5a). The individual expression profiles of these TFs were diverse, including patterns of peaking in expression prior to the initiation of aggregate formation, sharply increasing in expression at the onset of aggregation, peaking in expression in small or large aggregates, or constantly increasing in expression (Fig. 5, Extended Data Fig. 9, Fig. S3, and Data S9). These patterns likely reflect the functions of different TFs in regulating the expression of specific gene sets both prior to and throughout the *M. vibrans* aggregation process. A number of these TFs are thus far only found in Opisthokonta, Holozoa, or Filozoa and show strong expression patterns (Fig. 5b–c). For example, the differentially expressed T-box TF, specifically Tbx7, which has only been found in Holozoa,[44] had a sharp peak in expression at 12 h, prior to the initiation of aggregation. Tbox TFs regulate metazoan development.[44] On the other hand, the putative SOX-like TF had a sharp peak in expression at 33 h, prior to the formation of large mature aggregates. SOX and SOX-like TFs have so far only been found in Filozoa. Choanoflagellate SOX can induce the formation of mouse stem cells from somatic cells, mirroring the function of SOX in animals.[42] Both of the

differentially expressed RUNX and p53 TFs, which originated in the urholozoan and uropisthokont ancestors,[45] sharply increased in expression at the onset of aggregation (Fig. 5b–d). The RHD TF on the other hand, which emerged in Opisthokonta and includes TFs regulating immunity, development, and differentiation in animals,[46] reached peak expression in large mature aggregates.

Supplementary Discussion 3 – Phagocytosis, meiosis, and mating related genes are expressed during *M. vibrans* aggregation

There are few highly conserved phagocytosis components found across eukaryotes that do not have prokaryotic homologs, and these genes can have diverse functions. However, phagocytosis depends on actin cytoskeleton remodelling and branched filament formation through the action of the Arp2/3 protein complex.[47] Unicellular holozoans have also previously been shown to encode diverse proteins associated with actin-based cellular protrusions necessary for phagocytosis.[4] We thus searched for homologs of such phagocytosis genes in the *M. vibrans* genome and investigated their individual expression profiles, in addition to investigating the patterns of DEGs in related KEGG pathways (Extended Data Fig. 5, Fig. S5, and Data S10). We found that different actin homologs were expressed both early and later during aggregation. In terms of the Arp2/3 complex, all components were highly upregulated until just prior to the formation of small aggregates before dropping in expression. Some genes encoding actin remodelling proteins were upregulated initially (i.e., cofilin, profilin, and twinfilin) before a switch where the majority became upregulated as large mature aggregates formed (i.e., Ena/VASO, diaphanous-related formins, formins, villin, gelsolin family, and coronin), alongside the upregulation of several genes involved in the WAVE regulatory complex. These results suggest an initial burst in actin polymerization prior to aggregate formation later followed by actin remodelling in large mature aggregates. The KEGG pathway DEG patterns were consistent with this as endocytosis, phagosome, and lysosome pathways had a higher proportion of DEGs that were net downregulated, and thus related genes were highly expressed prior to aggregate formation. While cytoskeleton proteins, efferocytosis, motor proteins, and regulation of actin cytoskeleton pathways had a higher proportion of DEGs that were net upregulated, suggesting an increase in expression after aggregate formation. Together, these results are consistent with our experimental findings that bacterial feeding through phagocytosis is occurring in *M. vibrans* aggregates. Consistent with our results showing increased growth in *M vibrans* aggregates (Fig. 6c), *S. rosetta* colonial multicellular rosettes have previously been shown to increase feeding efficiency through higher rates of food vacuole formation compared to unicellular cells.[48]

Mating has previously been demonstrated in the choanoflagellate *S. rosetta* in response to stress from nutrient limitation [49] or bacterial chondroitinases, such as produced by *Vibrio fischeri*. [50] Genes involved in meiosis and in syngamy, which is indicative of mating, are found across diverse eukaryotes including all sequenced lineages of unicellular holozoans. [51] HAP2 and GEX1 in particular are associated with syngamy as they are essential for cell membrane (plasmogamy) and nuclear (karyogamy) fusion, respectively, and have been used as marker genes for these processes, including in unicellular holozoans.[52-54] In particular, an extensive complement of genes from the meiosis gene toolkit[55] has previously been detected in both *M. brevicollis* [56] and *C. owczarzaki.* [8] Given the enrichment of GO terms related to meiosis during *M. vibrans* aggregation, we searched for gene homologs of meiosis- and syngamy-related proteins and investigated their individual gene expression profiles (Fig. 6e, Extended Data Fig. 10, Fig. S5–6, and Data S10). We focused on proteins previously investigated in a study on the distribution of meiosis and syngamy across Amoebozoa.[57] We found that *M. vibrans* has nearly a full complement of genes encoding meiosis- and syngamy-related proteins, with the exception of REC8 (Data S10), which is also missing in *M. brevicollis*. Using the strict cutoff for identifying differentially expressed genes (adjusted p-value < 0.0000000001), we found that proteins involved in sister chromatid cohesion (SMC1, SMC3, and RAD21), homologous recombination (RAD51A, HOP2, and MND1), gene conversion by mismatch repair (MSH2, MSH6, and PMS2), and karyogamy (GEX1) had the highest gene expression just prior to aggregate formation or in small aggregates (Extended Data Fig. S10, and Fig. S5–6). On the other hand, proteins involved in introducing double-strand breaks (MRE11 and RAD50), crossover resolution pathway I (MLH1), and plasmogamy (HAP2) increased in gene expression throughout aggregation (Extended Data Fig. S10, and Fig. S5–6). It may be that only a subpopulation of cells in aggregates is undergoing meiosis and syngamy, which would result in lower levels of overall gene expression. To address this, we checked if additional genes encoding proteins involved in meiosis were found to be differentially expressed when using higher significance cutoffs (adjusted p-values < 0.00001 and < 0.001; Extended Data Fig. S10 and Fig. S6). With the higher cutoffs, we also found that genes encoding proteins involved in synaptonemal complex assembly including homolog pairing (PCH2) and the ZMM complex (MSH4 and MSH5),

and crossover resolution pathway II (MUS81 and MMS4) were differentially expressed during the *M. vibrans* aggregation process. Additional genes encoding proteins involved in introducing double-stranded breaks (SPO11), crossover resolution pathway I (MLH3 and EXO1), and gene conversion by mismatch repair (PMS1) were also captured as differentially expressed with the higher significance cutoffs.

Overall, our results are suggestive of meiosis and mating occurring in *M. vibrans* aggregates. However, future efforts to experimentally verify this finding are needed. As an aside, we also found that *M. vibrans* encodes a putative chondroitin lyase (g557) as it has the same predicted glycosaminoglycan (GAG) lyase domains (PF08124, PF02278, and PF02884) as EroS, which is the *V. fischeri* mating inducer.[50] The gene increases over four times in expression over *M. vibrans* aggregation and is a top hit to the *S. rosetta* sequence XP_004988474.1, which is also a putative chondroitin lyase.

SUPPLEMENTARY FIGURES

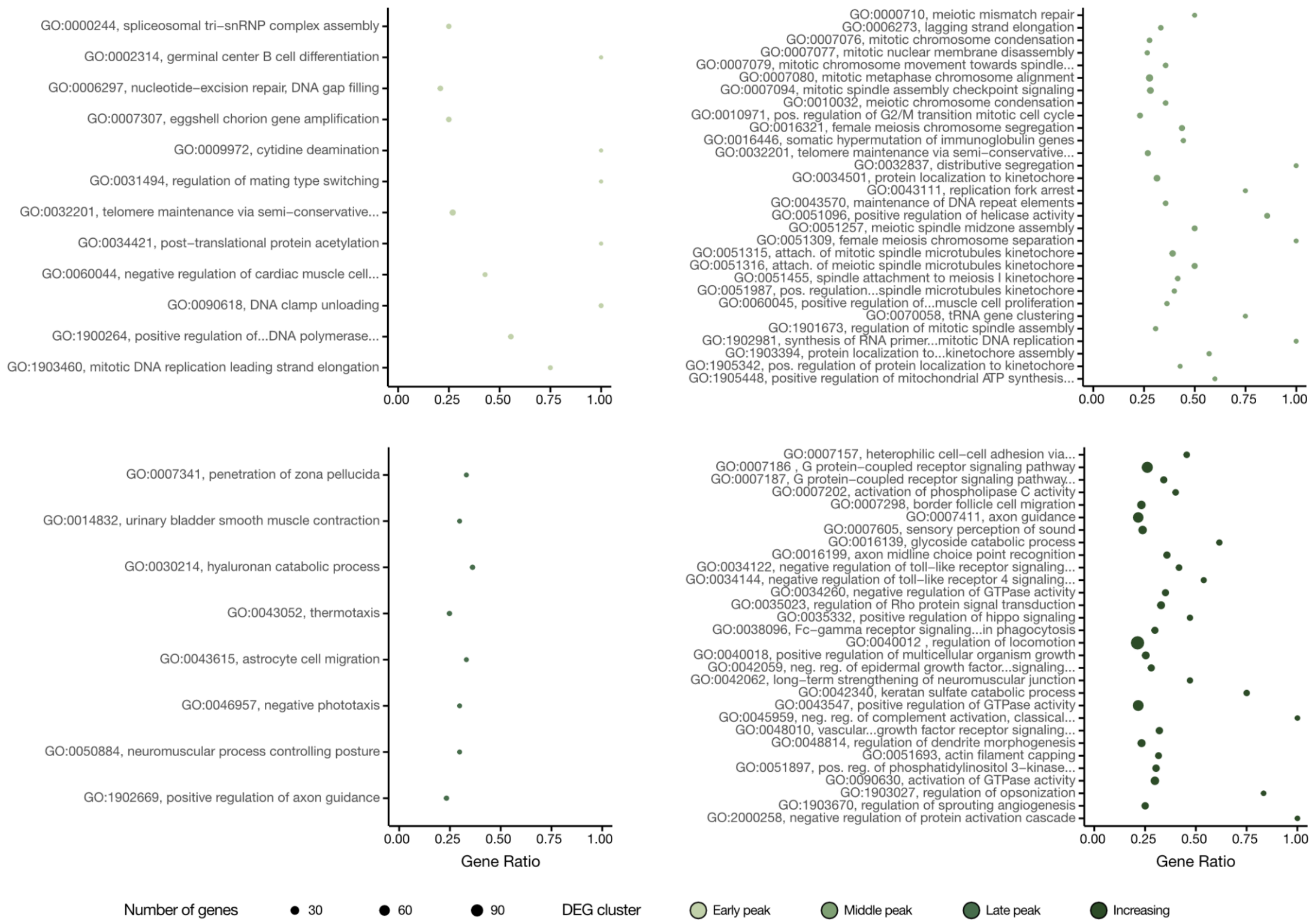

Figure S1. Dotplot overview of the top 30 enriched GO terms with the lowest weighted Fisher p-values ($p \leqslant 0.001$) across each of the four net upregulated differentially expressed gene (DEG) clusters. Gene ratio indicates the proportion of genes assigned to each GO term that are cluster DEGs, terms with less than 0.2 were excluded. Dot size indicates the number of cluster DEGs assigned to the corresponding GO term. Colours indicate the DEG cluster. See Data S8 for all enriched GO terms and complete names. Analyses were performed from RNAseq data of n=4 independent cultures for each time point.

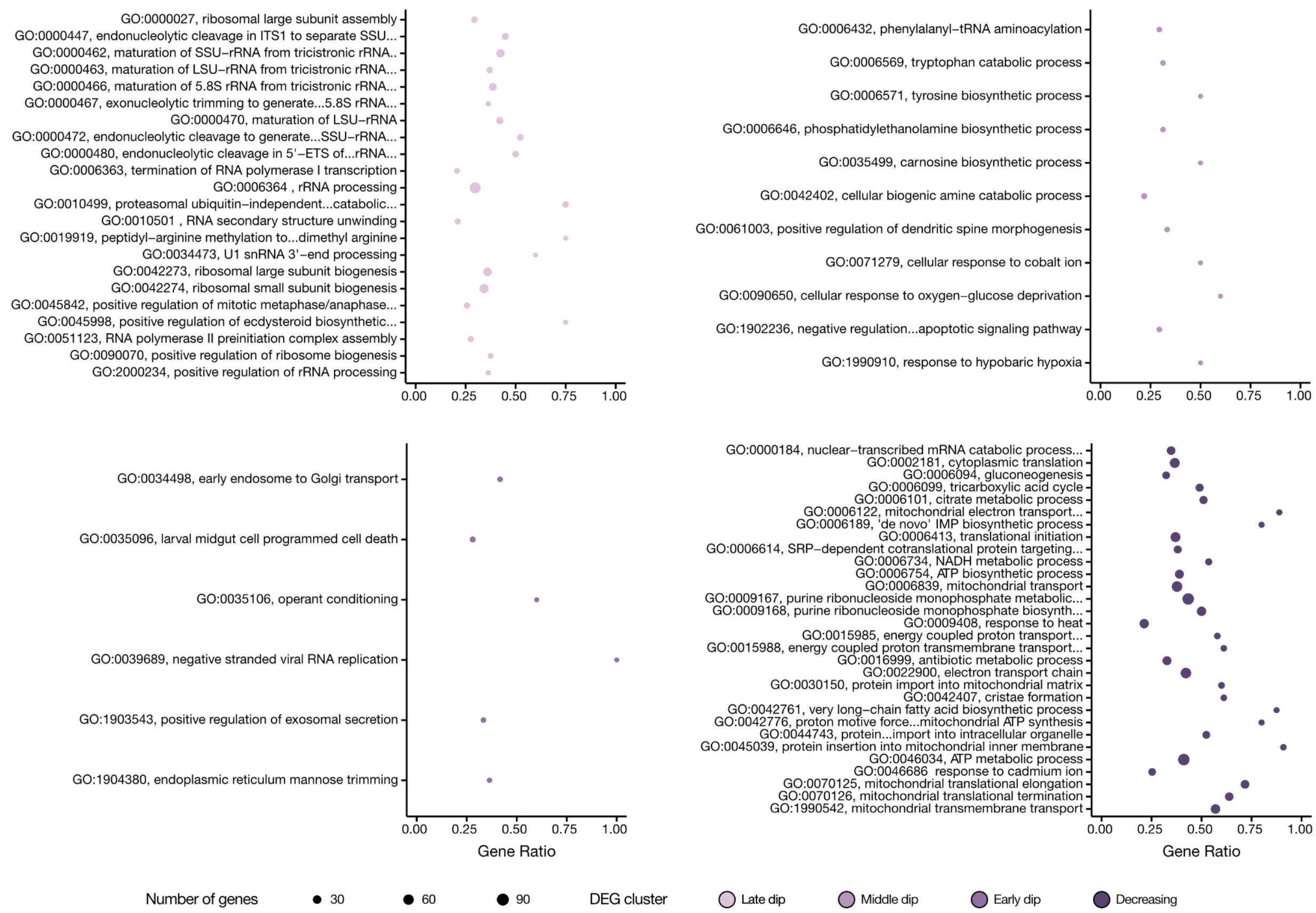

Figure S2. Dotplot overview of the top 30 enriched GO terms with the lowest weighted Fisher p-values ($p \leqslant 0.001$) across each of the four net downregulated differentially expressed gene (DEG) clusters. Gene ratio indicates the proportion of genes assigned to each GO term that are cluster DEGs, terms with less than 0.2 were excluded. Dot size indicates the number of cluster DEGs assigned to the corresponding GO term. Colours indicate the DEG cluster. See Data S8 for all enriched GO terms and complete names. Analyses were performed from RNAseq data of n=4 independent cultures for each time point.

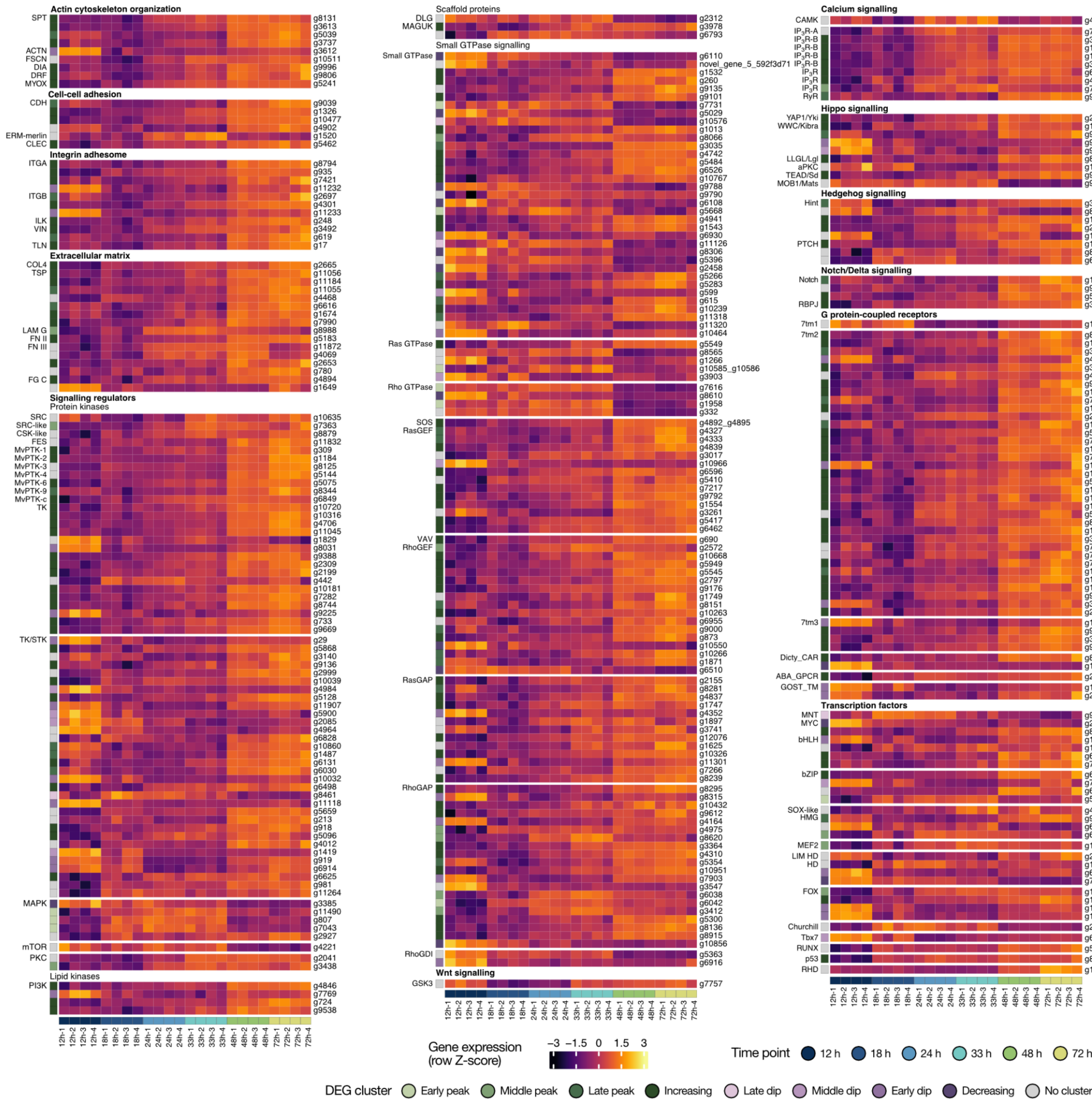

Figure S3. Heatmaps showing the gene expression patterns of proteins involved in animal multicellularity that were significantly differentially expressed across the *M. vibrans* aggregation time series. The bottom row indicates the time point according to the legend and the left column DEG cluster assignment. Gene expression values were centered and row-scaled using the variance-stabilizing transformed and normalized gene counts. Analyses were performed from RNAseq data of n=4 independent cultures for each time point. See Fig. 5 and Extended Data Fig. 9 for definitions of shortened names and acronyms.

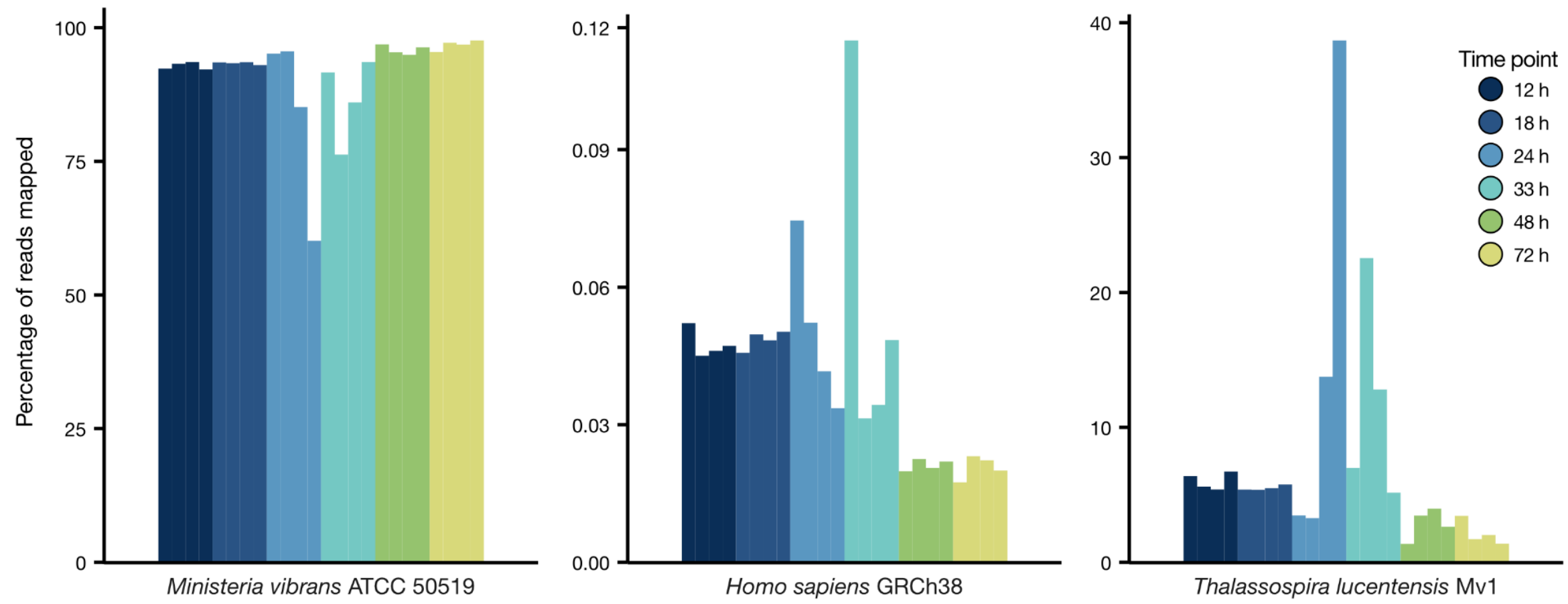

Figure S4. Percentage of RNA-sequencing reads from each of the four replicates at each time point in the aggregation time series mapped against the *M. vibrans* ATCC 50519, *Homo sapiens* GRCh38, and *Thalassospira lucentensis* Mv1 genomes. Analyses were performed from RNAseq data of n=4 independent cultures for each time point. Each culture is represented as its own bar.

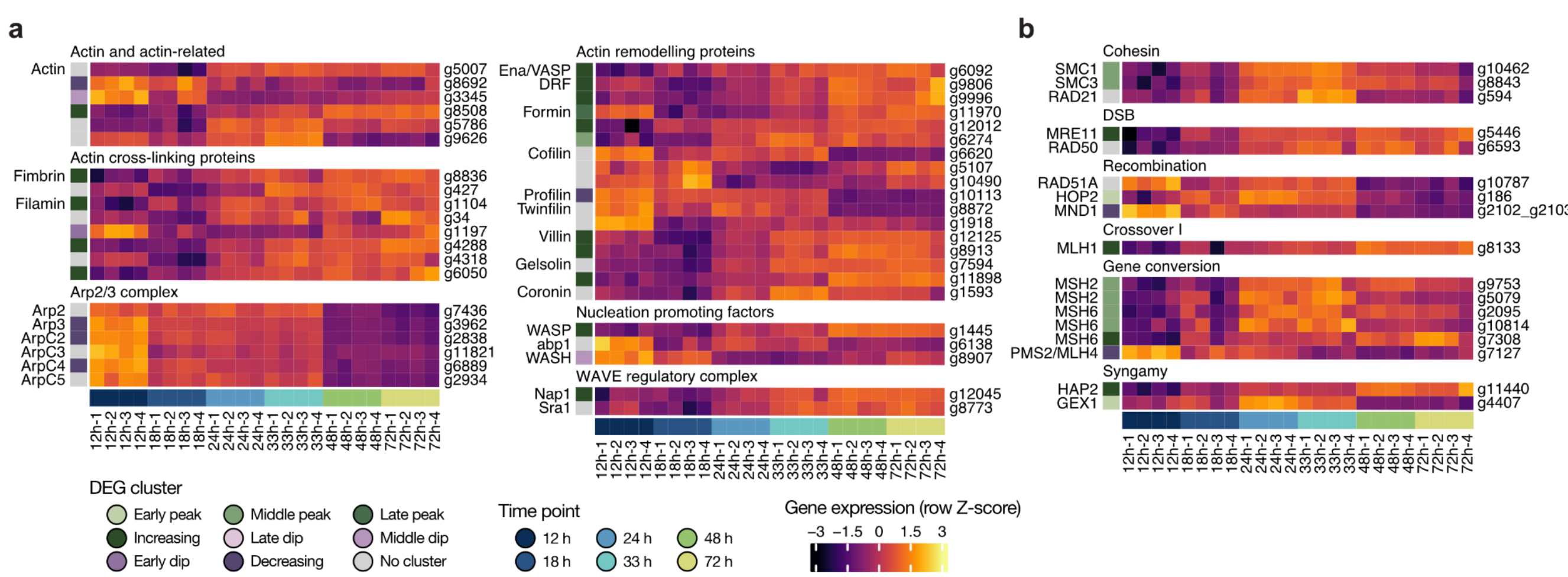

Figure S5. Heatmaps showing the gene expression patterns of proteins involved in a) phagocytosis and b) meiosis and syngamy that were significantly differentially expressed across the *M. vibrans* aggregation time series. The bottom row indicates the time point according to the legend and the left column DEG cluster assignment. Gene expression values for replicates at each time point were centered and scaled using the variance-stabilizing transformed and normalized gene counts. Analyses were performed from RNAseq data of n=4 independent cultures for each time point. See Extended Data Fig. 10 for definitions of shortened names and acronyms.

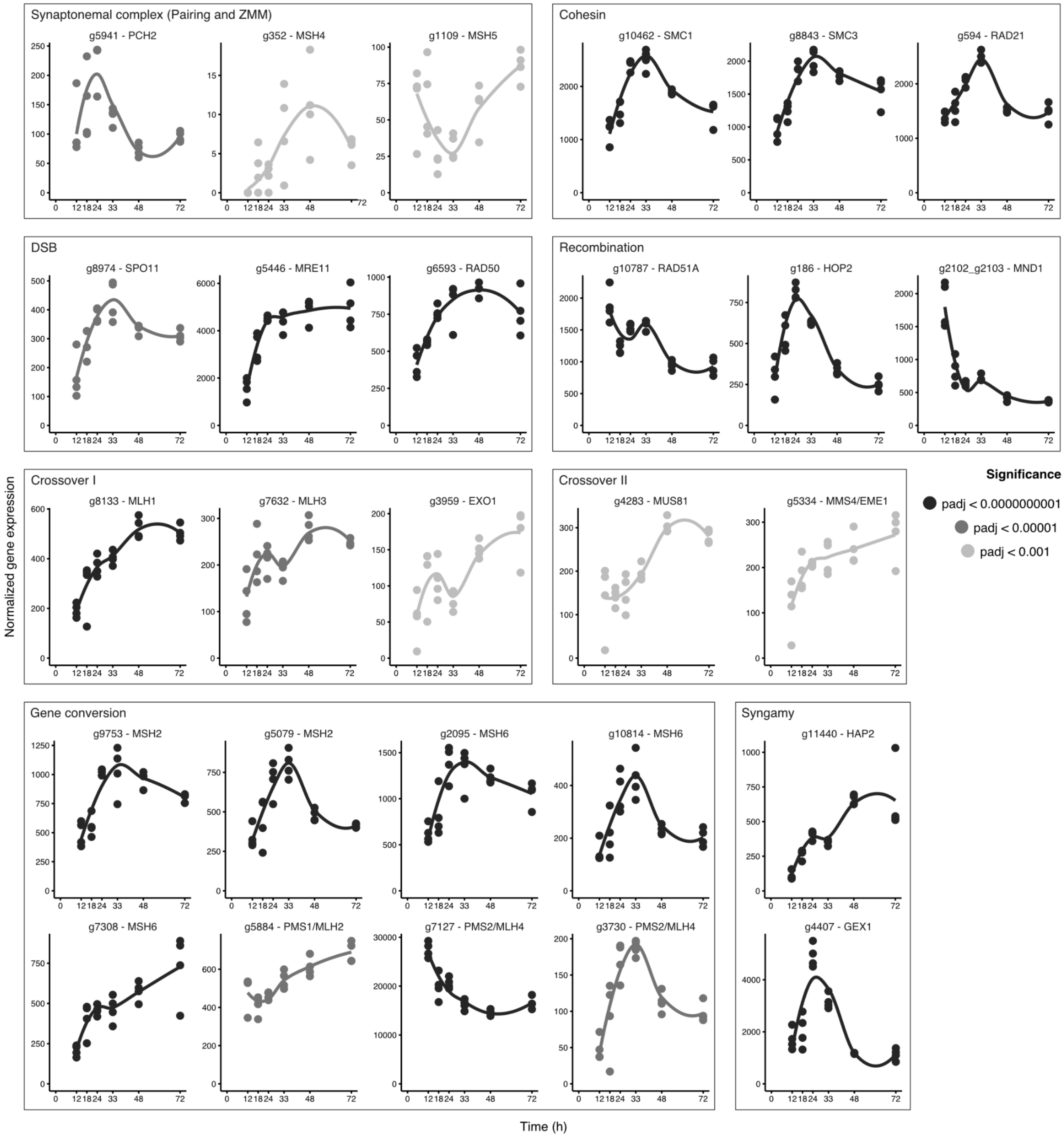

Figure S6. Normalized gene expression counts of proteins associated with meiosis and syngamy that are differentially expressed over the time course of aggregation. Points and trends are coloured according to the legend based on different adjusted p-value significance level cutoffs. Statistical significance was assessed using the likelihood ratio test (LRT) as implemented in DESeq2 [58] with p-values adjusted for multiple testing using independent hypothesis weighting (IHW). [59] See Data S10 for exact adjusted p-values.

Meiosis or syngamy specific proteins include PCH2, MSH4, MSH5, SPO11, HOP2, MND1, HAP2, and GEX1. See Extended Data Fig. 10 for definitions of shortened names and acronyms.

SUPPLEMENTARY DATA DESCRIPTIONS

Data S1. 16S rDNA amplicon sequencing of xenic *M. vibrans* ATCC 50519.

Data S2. 16S rDNA sequence from the isolate *T. lucentensis* Mv1.

Data S3. 16S rDNA amplicon sequencing of monoxenic *M. vibrans* with *T .lucentensis* Mv1 generated from the ATCC 50519 strain.

Data S4. Summary of all sequence data submitted to NCBI under BioProject PRJNA1229532 with sample information, BioSample, and SRA accessions.

Data S5. Functional annotations of *M. vibrans* ATCC 50519 genes.

Data S6. RNA-sequencing gene expression counts including raw, normalized, and normalized and variance-stabilizing transformed data.

Data S7. Differentially expressed genes (DEGs) during *M. vibrans* ATCC 50519 aggregation, including adjusted p-values, log2 fold change between 12 and 72 h, DEG cluster assignment, and functional annotations.

Data S8. Gene Ontology (GO) terms enriched in each DEG cluster.

Data S9. Genes encoding multicellularity-related proteins searched for and encoded in the *M. vibrans* ATCC 50519 genome, alongside functional annotations, predicted gene age information, and DEG cluster assignments.

Data S10. Genes encoding phagocytosis, meiosis, and mating related proteins searched for and encoded in the *M. vibrans* ATCC 50519 genome, alongside functional annotations and differential expression information.

Data S11. 18S rDNA sanger sequencing of *M. vibrans* and *Paraphysomonas imperforata* isolated from DSM 114853.

Data S12. 16S rDNA amplicon sequencing of monoxenic *M. vibrans* with *T. lucentensis* Mv1 generated from the DSM 114853 strain.

Data S13. 16S rDNA amplicon sequencing of xenic *M. vibrans* DSM 114853 (after removing *P. imperforata*).

SUPPLEMENTARY VIDEO DESCRIPTIONS

Video S1. Aggregation of monoxenic *M. vibrans* Tong (ATCC 50519) with *T. lucentensis* Mv1 over 72h.

Video S2. Aggregation of aphidicolin-treated monoxenic *M. vibrans* Tong (ATCC 50519) with *T. lucentensis* Mv1 over 36h.

Video S3. Aggregation of monoxenic *M.vibrans* Tong (ATCC 50519) with *T. lucentensis* over 1140h (60 days).

Video S4. 3D image stacks of a double-stained *M. vibrans* cell.

Video S5. Time-lapse video of *M. vibrans* cell fusion event by focusing on the surrounding of aggregates

Video S6. Initial xenic *M.vibrans* L27 culture (DSM 114853) grown in 5% SWC. Two different eukaryotic cells are present. Larger cells that are generally solitary and occasionally swimming were identified by 18S amplicon sequencing to be *Paraphysomonas imperforate*. Smaller cells that are generally in multicellular clusters were identified by 18S amplicon sequencing to be *M. vibrans*.

Video S7. Aggregation of xenic *M.vibrans* L27 (DSM 114853) over 72h.

Video S8. Aggregation of monoxenic *M.vibrans* L27 (DSM 114853) with bacterium *T. lucentensis* Mv1 over 72h.

REFERENCES

1 King, N. *et al.* The genome of the choanoflagellate Monosiga brevicollis and the origin of metazoans. *Nature* **451**, 783-788 (2008). https://doi.org/10.1038/nature06617

2 Abedin, M. & King, N. The premetazoan ancestry of cadherins. *Science* **319**, 946-948 (2008). https://doi.org/10.1126/science.1151084

3 Sebé-Pedrós, A., Roger, A. J., Lang, F. B., King, N. & Ruiz-Trillo, I. Ancient origin of the integrin-mediated adhesion and signaling machinery. *Proceedings of the National Academy of Sciences* **107**, 10142-10147 (2010). https://doi.org/10.1073/pnas.1002257107

4 Sebé-Pedrós, A. *et al.* Insights into the origin of metazoan filopodia and microvilli. *Mol Biol Evol* **30**, 2013-2023 (2013). https://doi.org/10.1093/molbev/mst110

5 Sebe-Pedros, A., Grau-Bove, X., Richards, T. A. & Ruiz-Trillo, I. Evolution and classification of myosins, a paneukaryotic whole-genome approach. *Genome biology and evolution* **6**, 290-305 (2014). https://doi.org/ 10.1093/gbe/evu013

6 Nichols, S. A., Roberts, B. W., Richter, D. J., Fairclough, S. R. & King, N. Origin of metazoan cadherin diversity and the antiquity of the classical cadherin/β-catenin complex. *Proceedings of the National Academy of Sciences* **109**, 13046-13051 (2012). https://doi.org/10.1073/pnas.1120685109

7 Miller, P. W., Clarke, D. N., Weis, W. I., Lowe, C. J. & Nelson, W. J. The evolutionary origin of epithelial cell–cell adhesion mechanisms. *Current topics in membranes* Vol. 72 267-311 (Elsevier, 2013).

8 Suga, H. *et al.* The Capsaspora genome reveals a complex unicellular prehistory of animals. (2013). https://doi.org/ 10.1038/ncomms3325

9 Williams, F., Tew, H. A., Paul, C. E. & Adams, J. C. The predicted secretomes of Monosiga brevicollis and Capsaspora owczarzaki, close unicellular relatives of metazoans, reveal new insights into the evolution of the metazoan extracellular matrix. *Matrix Biology* **37**, 60-68 (2014). https://doi.org/10.1016/j.matbio.2014.02.002

10 Cortese, M. *et al.* Spectrin βV adaptive mutations and changes in subcellular location correlate with emergence of hair cell electromotility in mammalians. *Proc Natl Acad Sci U S A* **114**, 2054-2059 (2017). https://doi.org/10.1073/pnas.1618778114

11 Hehenberger, E. *et al.* Novel predators reshape holozoan phylogeny and reveal the presence of a two-component signaling system in the ancestor of animals. *Curr Biol* **27**, 2043-2050. e2046 (2017). https://doi.org/10.1016/j.cub.2017.06.006

12 Grau-Bove, X. *et al.* Dynamics of genomic innovation in the unicellular ancestry of animals. *elife* **6**, e26036 (2017). https://doi.org/10.7554/eLife.26036

13 López-Escardó, D. *et al.* Reconstruction of protein domain evolution using single-cell amplified genomes of uncultured choanoflagellates sheds light on the origin of animals. *Philos. Trans. R. Soc. B, Biol. Sci.* **374** (2019). https://doi.org/10.1098/rstb.2019.0088

14 Shabardina, V., Kashima, Y., Suzuki, Y. & Makalowski, W. Emergence and evolution of ERM proteins and merlin in metazoans. *Genome Biol Evol* **12**, 3710-3724 (2020). https://doi.org/10.1093/gbe/evz265

15 Tikhonenkov, D. V. *et al.* New lineage of microbial predators adds complexity to reconstructing the evolutionary origin of animals. *Curr Biol* **30**, 4500-4509.e4505 (2020). https://doi.org/10.1016/j.cub.2020.08.061
16 Ansel, M., Ramachandran, K., Dey, G. & Brunet, T. Origin and evolution of microvilli. *Biol Cell* **116**, e2400054 (2024). https://doi.org/10.1111/boc.202400054
17 Baines, A. J. Evolution of spectrin function in cytoskeletal and membrane networks. *Biochem Soc Trans* **37**, 796-803 (2009). https://doi.org/10.1042/bst0370796
18 Kanchanawong, P. & Calderwood, D. A. Organization, dynamics and mechanoregulation of integrin-mediated cell-ECM adhesions. *Nat Rev Mol Cell Biol* **24**, 142-161 (2023). https://doi.org/10.1038/s41580-022-00531-5
19 Kang, S. *et al.* The integrin-mediated adhesive complex in the ancestor of animals, fungi, and amoebae. *Curr Biol* **31**, 3073-3085.e3073 (2021). https://doi.org/10.1016/j.cub.2021.04.076
20 Boureux, A., Vignal, E., Faure, S. & Fort, P. Evolution of the Rho family of ras-like GTPases in eukaryotes. *Mol Biol Evol* **24**, 203-216 (2007). https://doi.org/10.1093/molbev/msl145
21 Suga, H. *et al.* Genomic survey of premetazoans shows deep conservation of cytoplasmic tyrosine kinases and multiple radiations of receptor tyrosine kinases. *Sci Signal* **5**, ra35 (2012). https://doi.org/10.1126/scisignal.2002733
22 de Mendoza, A., Sebé-Pedrós, A. & Ruiz-Trillo, I. The evolution of the GPCR signaling system in eukaryotes: modularity, conservation, and the transition to metazoan multicellularity. *Genome Biol Evol* **6**, 606-619 (2014). https://doi.org/10.1093/gbe/evu038
23 Babonis, L. S. & Martindale, M. Q. Phylogenetic evidence for the modular evolution of metazoan signalling pathways. *Philos Trans R Soc Lond B Biol Sci* **372** (2017). https://doi.org/10.1098/rstb.2015.0477
24 Philippon, H., Brochier-Armanet, C. & Perrière, G. Evolutionary history of phosphatidylinositol- 3-kinases: ancestral origin in eukaryotes and complex duplication patterns. *BMC Evol Biol* **15**, 226 (2015). https://doi.org/10.1186/s12862-015-0498-7
25 Cai, X., Wang, X., Patel, S. & Clapham, D. E. Insights into the early evolution of animal calcium signaling machinery: a unicellular point of view. *Cell Calcium* **57**, 166-173 (2015). https://doi.org/10.1016/j.ceca.2014.11.007
26 Huang, E. *et al.* Reconstructing the deep phylogeny of the MAPK signaling network: functional specialization via multi-tier coevolutionary expansion. *bioRxiv*, 2024.2010. 2001.616093 (2024). https://doi.org/10.1101/2024.10.01.616093
27 de Mendoza, A., Suga, H. & Ruiz-Trillo, I. Evolution of the MAGUK protein gene family in premetazoan lineages. *BMC Evol Biol* **10**, 93 (2010). https://doi.org/10.1186/1471-2148-10-93
28 Nilkant, R. *et al.* Evolution of the Tri-PDZ Domain in PSD95 (DLG-4 Gene). *Mol Biol Evol* **42** (2025). https://doi.org/10.1093/molbev/msaf309
29 Alzayady, K. J. *et al.* Tracing the evolutionary history of inositol, 1, 4, 5-trisphosphate receptor: insights from analyses of Capsaspora owczarzaki Ca2+ release channel orthologs. *Mol Biol Evol* **32**, 2236-2253 (2015). https://doi.org/10.1093/molbev/msv098

30 Adamska, M. *et al.* The evolutionary origin of hedgehog proteins. *Curr Biol* **17**, R836-837 (2007). https://doi.org/10.1016/j.cub.2007.08.010
31 Mosaddeghzadeh, N. & Ahmadian, M. R. The RHO Family GTPases: mechanisms of regulation and signaling. *Cells* **10** (2021). https://doi.org/10.3390/cells10071831
32 Rodríguez-Fdez, S. & Bustelo, X. R. The Vav GEF family: an evolutionary and functional perspective. *Cells* **8** (2019). https://doi.org/10.3390/cells8050465
33 Sebé-Pedrós, A., Zheng, Y., Ruiz-Trillo, I. & Pan, D. Premetazoan origin of the hippo signaling pathway. *Cell Rep* **1**, 13-20 (2012). https://doi.org/10.1016/j.celrep.2011.11.004
34 Phillips, J. E., Santos, M., Konchwala, M., Xing, C. & Pan, D. Genome editing in the unicellular holozoan Capsaspora owczarzaki suggests a premetazoan role for the Hippo pathway in multicellular morphogenesis. *Elife* **11** (2022). https://doi.org/10.7554/eLife.77598
35 Snell, E. A. *et al.* An unusual choanoflagellate protein released by Hedgehog autocatalytic processing. *Proc Biol Sci* **273**, 401-407 (2006). https://doi.org/10.1098/rspb.2005.3263
36 Hausmann, G., von Mering, C. & Basler, K. The hedgehog signaling pathway: where did it come from? *PLoS Biol* **7**, e1000146 (2009). https://doi.org/10.1371/journal.pbio.1000146
37 Bürglin, T. R. Evolution of hedgehog and hedgehog-related genes, their origin from Hog proteins in ancestral eukaryotes and discovery of a novel Hint motif. *BMC Genomics* **9**, 127 (2008). https://doi.org/10.1186/1471-2164-9-127
38 Shalchian-Tabrizi, K. *et al.* Multigene phylogeny of choanozoa and the origin of animals. *PLoS One* **3**, e2098 (2008). https://doi.org/10.1371/journal.pone.0002098
39 Richter, D. J., Fozouni, P., Eisen, M. B. & King, N. Gene family innovation, conservation and loss on the animal stem lineage. *Elife* **7** (2018). https://doi.org/10.7554/eLife.34226
40 Garcia De Las Bayonas, A. & King, N. G-protein-coupled receptor diversity and evolution in the closest living relatives of metazoa. *Elife* **14** (2025). https://doi.org/10.7554/eLife.107467
41 Chu, J. S., Baillie, D. L. & Chen, N. Convergent evolution of RFX transcription factors and ciliary genes predated the origin of metazoans. *BMC Evol Biol* **10**, 130 (2010). https://doi.org/10.1186/1471-2148-10-130
42 Gao, Y. *et al.* The emergence of Sox and POU transcription factors predates the origins of animal stem cells. *Nat Commun* **15**, 9868 (2024). https://doi.org/10.1038/s41467-024-54152-x
43 Sebé-Pedrós, A., de Mendoza, A., Lang, B. F., Degnan, B. M. & Ruiz-Trillo, I. Unexpected repertoire of metazoan transcription factors in the unicellular holozoan Capsaspora owczarzaki. *Mol Biol Evol* **28**, 1241-1254 (2011). https://doi.org/10.1093/molbev/msq309
44 Sebé-Pedrós, A. *et al.* Early evolution of the T-box transcription factor family. *Proc Natl Acad Sci U S A* **110**, 16050-16055 (2013). https://doi.org/10.1073/pnas.1309748110
45 de Mendoza, A. & Sebé-Pedrós, A. Origin and evolution of eukaryotic transcription factors. *Curr Opin Genet Dev* **58-59**, 25-32 (2019). https://doi.org/10.1016/j.gde.2019.07.010

46 Leger, M. M., Ros-Rocher, N., Najle, S. R. & Ruiz-Trillo, I. Rel/NF-κB transcription factors emerged at the onset of Opisthokonts. *Genome Biol Evol* **14** (2022). https://doi.org/10.1093/gbe/evab289
47 Yutin, N., Wolf, M. Y., Wolf, Y. I. & Koonin, E. V. The origins of phagocytosis and eukaryogenesis. *Biol Direct* **4**, 9 (2009). https://doi.org/10.1186/1745-6150-4-9
48 L'Etoile, N. J. & King-Smith, C. Rosette colonies of Choanoflagellates (Salpingoeca rosetta) show increased food vacuole formation compared with single swimming cells. *J Eukaryot Microbiol* **67**, 263-267 (2020). https://doi.org/10.1111/jeu.12780
49 Levin, T. C. & King, N. Evidence for sex and recombination in the choanoflagellate Salpingoeca rosetta. *Curr Biol* **23**, 2176-2180 (2013). https://doi.org/10.1016/j.cub.2013.08.061
50 Woznica, A., Gerdt, J. P., Hulett, R. E., Clardy, J. & King, N. Mating in the closest living relatives of animals is induced by a vacterial Chondroitinase. *Cell* **170**, 1175-1183.e1111 (2017). https://doi.org/10.1016/j.cell.2017.08.005
51 Hofstatter, P. G. & Lahr, D. J. G. All eukaryotes are sexual, unless proven otherwise: many so-called asexuals present meiotic machinery and might be able to have sex. *Bioessays* **41**, e1800246 (2019). https://doi.org/10.1002/bies.201800246
52 Wong, J. L. & Johnson, M. A. Is HAP2-GCS1 an ancestral gamete fusogen? *Trends Cell Biol* **20**, 134-141 (2010). https://doi.org/10.1016/j.tcb.2009.12.007
53 Ning, J. *et al.* Comparative genomics in Chlamydomonas and Plasmodium identifies an ancient nuclear envelope protein family essential for sexual reproduction in protists, fungi, plants, and vertebrates. *Genes Dev* **27**, 1198-1215 (2013). https://doi.org/10.1101/gad.212746.112
54 Speijer, D., Lukeš, J. & Eliáš, M. Sex is a ubiquitous, ancient, and inherent attribute of eukaryotic life. *Proc Natl Acad Sci U S A* **112**, 8827-8834 (2015). https://doi.org/10.1073/pnas.1501725112
55 Hofstatter, P. G., Ribeiro, G. M., Porfírio-Sousa, A. L. & Lahr, D. J. G. The sexual ancestor of all eukaryotes: a defense of the "meiosis toolkit": a rigorous survey supports the obligate link between meiosis machinery and sexual recombination. *Bioessays* **42**, e2000037 (2020). https://doi.org/10.1002/bies.202000037
56 Carr, M., Leadbeater, B. S. & Baldauf, S. L. Conserved meiotic genes point to sex in the choanoflagellates. *J Eukaryot Microbiol* **57**, 56-62 (2010). https://doi.org/10.1111/j.1550-7408.2009.00450.x

58 Hofstatter, P. G., Brown, M. W. & Lahr, D. J. G. Comparative genomics supports sex and meiosis in diverse amoebozoa. *Genome Biol Evol* **10**, 3118-3128 (2018). https://doi.org/10.1093/gbe/evy241
59 Love, M. I., Huber, W. & Anders, S. Moderated estimation of fold change and dispersion for RNA-seq data with DESeq2. *Genome Biol* **15**, 550 (2014). https://doi.org/10.1186/s13059-014-0550-8
60 Ignatiadis, N., Klaus, B., Zaugg, J. B. & Huber, W. Data-driven hypothesis weighting increases detection power in genome-scale multiple testing. *Nat Methods* **13**, 577-580 (2016). https://doi.org/10.1038/nmeth.3885
