# Supplementary material for: A unicellular relative links aggregative multicellularity to animal origins
Source: Nature. 2026 Jun 9;656(8129):913–23. doi: 10.1038/s41586-026-10748-5 (PMC13366479; doi:10.1038/s41586-026-10748-5)
Supplement: Supplementary file 2 [file 41586_2026_10748_MOESM2_ESM.pdf]

nature portfolio

Corresponding author(s): Joseph P. Gerdt, Iñaki Ruiz-Trillo

Last updated by author(s): May 18, 2026

Reporting Summary

Nature Portfolio wishes to improve the reproducibility of the work that we publish. This form provides structure for consistency and transparency in reporting. For further information on Nature Portfolio policies, see our Editorial Policies and the Editorial Policy Checklist.

Statistics

For all statistical analyses, confirm that the following items are present in the figure legend, table legend, main text, or Methods section.

n/a	Confirmed	
☐	☒	The exact sample size (n) for each experimental group/condition, given as a discrete number and unit of measurement
☐	☒	A statement on whether measurements were taken from distinct samples or whether the same sample was measured repeatedly
☐	☒	The statistical test(s) used AND whether they are one- or two-sided<br>*Only common tests should be described solely by name; describe more complex techniques in the Methods section.*
☒	☐	A description of all covariates tested
☐	☒	A description of any assumptions or corrections, such as tests of normality and adjustment for multiple comparisons
☐	☒	A full description of the statistical parameters including central tendency (e.g. means) or other basic estimates (e.g. regression coefficient) AND variation (e.g. standard deviation) or associated estimates of uncertainty (e.g. confidence intervals)
☐	☒	For null hypothesis testing, the test statistic (e.g. F, t, r) with confidence intervals, effect sizes, degrees of freedom and P value noted<br>*Give P values as exact values whenever suitable.*
☒	☐	For Bayesian analysis, information on the choice of priors and Markov chain Monte Carlo settings
☒	☐	For hierarchical and complex designs, identification of the appropriate level for tests and full reporting of outcomes
☒	☐	Estimates of effect sizes (e.g. Cohen's d, Pearson's r), indicating how they were calculated

Our web collection on statistics for biologists contains articles on many of the points above.

Software and code

Policy information about availability of computer code

Data collection	BioTek Gen 5; FastQC v0.12.1; Trim Galore v0.6.10; BBSplit v39.01; SortMeRNA v 4.3.6; STAR v2.7.11b; RSEM v 1.3.3; nf-core/rnaseq v3.14.0; bcl2fastq v2.20.0
Data analysis	ImageJ v2.0.16.0; GraphPad Prism 10; BLAST+ v2.14.1; DIAMOND v2.1.9; eggNOG v5.0.0; Emapper v2.1.9; InterProScan v5.62-94.0; BlastKOALA v3.0, R v4.3.3; RStudio v2023.12.1+402; DESeq2 v1.42.1; vsn v3.70.0; IHW v1.30.0; DEGreport v1.38.5; clusterProfiler v4.10.1; TopGO v2.54.0; GOSemSim v2.28.1; Rrvgo v1.14.2; AnnotationForge v1.44.0; GeneBridge v0.99.2; ape v5.8.1; tidyverse v2.0.0; dplyr v1.1.4; plyr v1.8.9; ggplot2 v3.5.2; cowplot v1.1.3; viridis v0.6.5; pheatmap v1.0.12

For manuscripts utilizing custom algorithms or software that are central to the research but not yet described in published literature, software must be made available to editors and reviewers. We strongly encourage code deposition in a community repository (e.g. GitHub). See the Nature Portfolio guidelines for submitting code & software for further information.

Data

Policy information about availability of data

All manuscripts must include a data availability statement. This statement should provide the following information, where applicable:

- Accession codes, unique identifiers, or web links for publicly available datasets
- A description of any restrictions on data availability
- For clinical datasets or third party data, please ensure that the statement adheres to our policy

Sequencing reads have been deposited at the NCBI SRA database under BioProject PRJNA1229532 from RNA-sequencing (SRR32514592 to SRR32514615), 16S rDNA amplicon sequencing (SRR33469057 to SRR33469059, and SRS25957198), and the Thalassospira lucentensis strain Mv1 (SRR33469287). Information about which SRA accession corresponds to which sample alongside BioSample accessions can be found in Data S4. The assembled genome for the T. lucentensis strain Mv1 was also deposited at GenBank (GCA_050516545.1) under the same BioProject. Output from annotation tools, results files from the nf-core/rnaseq pipeline, and an R markdown script and html file for re-running R-analyses is provided at the figshare data repository at the DOI: https://doi.org/10.6084/m9.figshare.29027924. The following additional datasets and databases were also used (with associated accession numbers and links provided): Homo sapiens GRCh38 genome (GCF_000001405.40), Capsaspora owczarzaki genome v2 (GCF_000151315.2), Ministeria vibrans v1 genome (GCA_943704425.1), EukProt V3 TCS dataset (https://evocellbio.com/eukprot/?utm_source=chatgpt.com), NCBI's RefSeq and NR databases (https://www.ncbi.nlm.nih.gov/refseq/), KEGG GENES database (https://www.genome.jp/kegg/genes.html), eggNOG (http://eggnog5.embl.de), InterPro (https://www.ebi.ac.uk/interpro/), uniprot/swissprot (https://www.uniprot.org/), EukProt (https://evocellbio.com/eukprot/), and NR (https://www.ncbi.nlm.nih.gov/protein/).

Research involving human participants, their data, or biological material

Policy information about studies with human participants or human data. See also policy information about sex, gender (identity/presentation), and sexual orientation and race, ethnicity and racism.

Reporting on sex and gender	n/a
Reporting on race, ethnicity, or other socially relevant groupings	n/a
Population characteristics	n/a
Recruitment	n/a
Ethics oversight	n/a

Note that full information on the approval of the study protocol must also be provided in the manuscript.

Field-specific reporting

Please select the one below that is the best fit for your research. If you are not sure, read the appropriate sections before making your selection.

☒ Life sciences ☐ Behavioural & social sciences ☐ Ecological, evolutionary & environmental sciences

For a reference copy of the document with all sections, see nature.com/documents/nr-reporting-summary-flat.pdf

Life sciences study design

All studies must disclose on these points even when the disclosure is negative.

Sample size	Sample sizes were chosen based on experimental feasibility. Generally, a minimum of triplicates were used, except for one minor negative result in the extended data, where only duplicates were used and clearly presented.
Data exclusions	No data were excluded.
Replication	Generally, experiments were repeated at least once with similar results. The RNAseq experiment was only performed once with 4 replicates for each time point. The number of replicate images for every representative micrograph is stated in the figure captions
Randomization	Samples were not randomized, but all data for samples to be directly compared were collected in parallel on the same day to eliminate any day-to-day variation in the condition of the cells. Aggregation areas depend not only on conditions but also the number of cells. This potential covariate was accounted for by always testing the same cell density.
Blinding	Experiments were not blinded. Generally, the measurements were quantitative and lacked subjective interpretation.

Reporting for specific materials, systems and methods

We require information from authors about some types of materials, experimental systems and methods used in many studies. Here, indicate whether each material, system or method listed is relevant to your study. If you are not sure if a list item applies to your research, read the appropriate section before selecting a response.

Materials & experimental systems

n/a	Involved in the study
☒	☐ Antibodies
☐	☒ Eukaryotic cell lines
☒	☐ Palaeontology and archaeology
☒	☐ Animals and other organisms
☒	☐ Clinical data
☒	☐ Dual use research of concern
☒	☐ Plants

Methods

n/a	Involved in the study
☒	☐ ChIP-seq
☒	☐ Flow cytometry
☒	☐ MRI-based neuroimaging

Eukaryotic cell lines

Policy information about cell lines and Sex and Gender in Research

Cell line source(s)	American Type Culture Collection (ATCC) and Leibniz Institute German Collection of Microorganisms and Cell Cultures (DSMZ)
Authentication	Strains were validated by 18S amplicon sequencing
Mycoplasma contamination	Not tested for Mycoplasma contamination
Commonly misidentified lines (See ICLAC register)	n/a

Plants

Seed stocks	n/a
Novel plant genotypes	n/a
Authentication	n/a
