# Supplementary material for: A unicellular relative links aggregative multicellularity to animal origins
Source: Nature. 2026 Jun 9;656(8129):913–23. doi: 10.1038/s41586-026-10748-5 (PMC13366479; doi:10.1038/s41586-026-10748-5)
Supplement: Supplementary file 3 [file 41586_2026_10748_MOESM4_ESM.pdf]

nature portfolio

Peer Review File

A unicellular relative links aggregative multicellularity to animal origins

Corresponding Author: Dr Joseph Gerdt

This file contains all reviewer reports in order by version, followed by all author rebuttals in order by version.

Version 0:

Reviewer comments:

Referee #1

(Remarks to the Author)
The authors have studied the aggregation behaviour of *Ministeria vibrans*, a protist closely related to animals. *Ministeria* has long been recognized as a sister species of *Capsaspora*, composing the clade Filasterea together with the newly found species *Txikispora* and *Pigoraptor*. However, it has been underappreciated as a model organism to study the origin of animal multicellularity, as it does not form large colonies under an ordinary laboratory culture condition. It has already been known that the genome of *Ministeria* contains many "multicellularity" genes, similar to that of *Capsaspora*. *Ministeria*, which is free-living unlike *Capsaspora*, would thus become a unique model to understand the evolution of animal multicellularity once the missing condition for colony formation is found.
The authors have serendipitously found that the monoxenic culture with the single bacterial species *Thalassospira lucentensis* triggers the colony formation very efficiently. Moreover, they have found the colony very stable, successfully showing a consistent kinetics of aggregation behaviour in repeated experiments. This unique feature has allowed them to investigate the RNA expression of this unicellular holozoan in an unprecedented resolution. Their RNAseq data have demonstrated that many (actually nearly all) multicellularity genes involved in animal cell adhesion, signaling, and transcriptional regulation are deployed in the aggregation process, suggesting that the aggregation capacity of *Ministeria* reflects the primitive multicellularity acquired by the metazoan ancestor. They have also proposed a hypothesis that the ability of aggregation of the premetazoan ancestor would have provided selective advantages both for an efficient feeding by "bacteria farming" and for producing next generations through sexual reproduction.
The manuscript contains an important piece of work towards understanding how the first animal acquired the multicellularity. It would be of great interest for the general readers of Nature. However, there are a couple of concerns to be eliminated before the publication.

(1) No rationale provided why monoxenic culture with the specific bacterial species *T. lucentensis* can induce the aggregation in *Ministeria* culture.
Being free-living, *Ministeria* would hardly be in a monoxenic condition in nature. Such ability should immediately be removed during the course of evolution. Given that, the authors' claim that it was co-opted for constructing the primitive metazoan multicellular system does not make so much sense. Authors should thus conduct a couple of more experiments to understand why monoxenic culture of *T. lucentensis* can induce the aggregation and check if it is the only condition to do so. For example, they should check if other monoxenic culture with different bacterial species can form colonies, and how *Ministeria* would behave in dixenic (or even trixenic) culture with/without *T. lucentensis*. The authors should also eliminate the possibility that the original (multixenic) culture may contain a specific bacterial species inhibiting the colony formation. The authors should also provide a reasonable explanation why the colony formation occurs only in a monoxenic culture, in other words, why *Ministeria* does not form colonies in a multixenic condition. The authors have depicted the efficient feeding and mating as selective advantages of colony formation, but it would be the same in a multixenic condition.

(2) The evidence of sexual reproduction within the aggregation is too weak
The authors describe the up-regulation of some meiosis-related genes as good evidence of sexual reproduction occurring in the aggregation. However, the two genes picked up as examples (GEX1 and HAP2) are best known to be involved in membrane fusion of plant gametes after pollination, not in meiosis per se. The authors should check the expression of other key meiosis-related genes such as those involved in double-stranded DNA break and synaptonemal complex assembly.
If meiosis is vigorously occurring within the aggregation, it should contain many haploid cells, which the authors can confirm by simple DNA staining. The extremely stable *Ministeria* colony would allow them to do so.

Lastly, the authors should explain why Ministeria do both grow and reproduce sexually at the same time. When a life can grow well in a certain condition, it would rather avoid undergoing sexual reproduction, which is costly. What is the ecological relevance of doing growth and sexual reproduction at the same time?

The followings are some minor problems that the author may want to fix.
Line 274: In the legend of figure 5a, the colour code must be clearly described (being the same as in figure 4c).
Line 283-: In the legend of figure 5c, the commas and semicolons are in a reversed order. For instance, it has to be like "Spectrin, SPT; fascin, FSCN; …".
Line 336: With the reference 57, which lacks the comprehensive phylogenetic analysis including other tyrosine kinases, the receptor tyrosine kinase gene that the authors found upregulated would not confidently be identified as an eph receptor homologue. Please look more in detail and rephrase.
Line 470-472: A reference should be included.

Referee #2

(Remarks to the Author)
The evolution of complex multicellularity in animals remains a central and actively researched question in evolutionary biology. This study from Ruibao Li and colleagues contributes to the field by describing aggregative multicellularity and investigating its regulation in the filasterean Ministeria vibrans. The findings reveal that genes previously implicated in animal cell adhesion, signaling, and transcriptional regulation are active during this process. The authors conclude that filastereans possessed the capacity to aggregate using key genes associated with animal multicellularity, and suggest that enhanced feeding efficiency and sexual reproduction may have been evolutionary drivers of this aggregation.
The results are interesting and relevant. However, similar phenomena have been observed in other unicellular relatives of animals (e.g., Sebe-Pedros et al, 2014, https://pmc.ncbi.nlm.nih.gov/articles/PMC3870316/), which limits the novelty of the general concept. Nevertheless, the inclusion of M. vibrans, a filasterean that is phylogenetically distinct from both choanoflagellates and Capsaspora, adds a comparative value to the study.
This is a well-written manuscript. The authors clearly articulate their hypotheses and methodology, and they effectively situate their work within the context of existing literature. The experimental design is logical, and the data presented generally support the main conclusions (though see my criticisms below). Overall, the work is solid; however, it is somewhat disappointing that the results are primarily descriptive, offering limited novel and mechanistic insights.
Main comments:
1. The authors investigate whether disaggregated cells grow less effectively than those remaining in aggregates, and they indeed show that aggregated cells exhibit better growth. However, could the authors comment on the possibility that the disaggregation protocol itself may have interfered with cell division?
2. The authors show that Hap2 and Gex, genes known to be involved in mating, are upregulated during aggregation. There seems to be a misunderstanding in the conclusions because these two proteins function in cell and nuclear fusion, respectively and not directly in meiosis. Given that Hap2 is a well-established fusogen, it would have been important to provide evidence of cell fusion in the aggregates, which would support the claim that sexual reproduction plays a role in driving aggregative behavior.
3. It would strengthen the manuscript if the authors highlighted more clearly the divergence times from the last common ancestor of Filozoa, Capsaspora, and choanoflagellates.
4. It would be useful to clarify the genetic diversity of the original M. vibrans strain used in the study. Are the aggregating cells genetically distinct individuals, or is the culture largely clonal? This distinction affects the interpretation of the aggregation as a true social/multicellular behavior where competition and cheating could happen. Testing aggregation of mixed-strain cultures would help clarify this point (eg. M. vibrans ATCC and M. vibrans DSM 114853 both used in this study).
5. The ecological relevance of the observed aggregative phenotype could be discussed in more detail. Given that aggregation is only induced in monoxenic conditions, the relationship between this phenotype and the natural ecological niche of M. vibrans (which is likely diverse, given the also diverse bacterial community present in the original culture, lines 110-113) deserves further exploration.
6. The authors elegantly show that initial aggregate formation is independent of division (aphidicolin experiment, Fig.3). However, the reduced aggregate area in the aphidicolin condition compared to the control suggests that subsequent clonal division, coenocyte formation, or even mating may contribute to aggregate growth and maintenance. This possibility, also acknowledged by the authors, could be further explored using time-lapse microscopy and single-cell tracking to clarify the cellular dynamics and composition of long-term aggregates.
7. The role of Thalassospira bacteria in the aggregate formation remains somewhat ambiguous. Is aggregate formation triggered by direct physical contact, feeding or secreted factors? While the authors briefly acknowledge this uncertainty, the manuscript would benefit from experiments testing whether bacterial conditioned ASW alone can induce aggregation, or if live bacterial presence is required.
8. The authors mention the formation of a "putative bacterial biofilm" within the aggregate (Fig. 6). This should be better characterized using microscopy (e.g., staining for bacterial cells, extracellular matrix, TEM) and bacterial cell tracking to determine whether the bacteria actively proliferate within the aggregates. Can the authors be sure that the cells accidentally are not being 'stuck together' because of the bacterial biofim?
9. Overall, the figures are clear and well-presented. However, a few minor improvements are suggested:
- Fig. 3d and e: add labels on the plots so the reader can directly understand what they refer to without having to go through the legend. For both these plots a xenic control should also be provided

- Fig. 4b: color difference between the time points 12h and 18h is very difficult to see, especially in printed copy. Please change the color scheme or label the points in the plot
- Fig. 5a, Extended Data Figure 10 c – genes not belonging to a specific DEG cluster should also be included in these plots

Referee #3

(Remarks to the Author)
I co-reviewed this manuscript with one of the reviewers who provided the listed reports.

Referee #4

(Remarks to the Author)
In the article “A close unicellular relative reveals aggregative multicellularity was key to the evolution of animals” the authors reveal that the marine free-living bacterivorous filasterean Ministeria vibrans, a close protistan relative of animals, can undergo aggregation and thus describing another filasterean, which can an undergo aggregation. Interestingly, they found that Ministeria can form aggregates with reproducible kinetics that have long-term stability when cultured with an alphaproteobacterium (this is in striking difference to Capsaspora, the other filasterean which can undergo aggregation, where aggregates are induced by BFS and not a bacterium). In addition, the authors have characterized the transcriptional profile of aggregation and found many multicellularity genes involved in animal cell adhesion, signaling, and transcriptional regulation are used during this process. The authors conclude that cell aggregation that the last unicellular ancestor of animals had the capacity to aggregate by using key animal multicellularity genes and that feeding and sexual reproduction could have been the direct drivers of this process.

The topic is very compelling, significant, and original. The authors performed a wealth of carefully executed & convincing experiments. For example, just to name a few, they produced the very first monoxenic culture of Ministeria and performed experiments with replication blocker aphidicolin to strengthen their findings that Ministeria forms large clusters of hundreds of cells through aggregation and NOT cell division. Moreover, they show beautiful SEM and TEM images of Ministeria and thereby provide strong evidence for the presence of filopodial contacts and phagocytosed bacteria. Their results hint at aggregates being better at feeding and promoting opportunities for mating between cells. Thus, the here presented work advances the current state of knowledge and understanding. Their findings, together with previous findings that Capsaspora can aggregate, provide evidence that aggregation should be given greater emphasis on the different discussed hypotheses about the evolutionary origin of animals.

Several concerns need to be addressed:
- The authors mention in the abstract: It is unclear whether aggregation in these species is derived or ancestral, and its relevance for animal origins remains unknown. I wished this would have been discussed more clearly in the manuscript. Is it derived or ancestral?
- This touches on the point raised above: The title of the paper is: “A close unicellular relative reveals aggregative multicellularity was key to the evolution of animals”. Was it really key to the evolution of animals? There are other explanations, and the authors should discuss these in the paper.
- The key findings predominantly rely on transcriptomics. Would the authors have the chance/opportunity to use small molecule inhibitors or maybe even Crispr/Cas9 to provide evidence of direct participation in the aggregation of at least one upregulated protein/factor. If this is not possible (due to Ministeria not yet being fully developed as model organism, although inhibitor test should be feasible) this should be clearly mentioned in the manuscript.
- Have the authors identified new “multicellularity genes involved in animal cell adhesion, signaling, and transcriptional regulation” which previously were not known in filastereans? If so, these could be highlighted more prominently.

(Remarks on code availability)
I was not able to review the code (https://figshare.com/articles/dataset/dx_doi_org_10.6084/m9.figshare.29027924), as "page was not found"

Version 1:

Reviewer comments:

Referee #1

(Remarks to the Author)

Here are the answers to the authors of the revised manuscript.

1. Relevance of the Monoxeny of Colony-Inducing Bacteria
The authors have provided satisfactory evidence that *M. vibrans* is capable of aggregating in more natural, xenic environments. In addition, their new results using a second *M. vibrans* strain, together with the detailed bacterial profiling of the aggregation-inducing and aggregation-inhibiting species, further strengthen one of their key conclusions. These findings also lay a solid foundation for more detailed future investigations into the ecological relevance of *M. vibrans* aggregation.

2. Evidence of Sexual Reproduction
The authors have provided two new results: (1) a substantial number of meiosis-related genes are upregulated in the *M. vibrans* colony, in addition to the previously identified genes that are primarily involved in membrane fusion between gametes; and (2) a subpopulation of double-stained cells is observed in the colony after mixing two differently labeled cell populations.

The first result strongly suggests the presence of gamete production within the colony, which is highly intriguing. In light of this, the authors' revision that downplays the role of colony formation is somewhat disappointing. To be frank, if the manuscript had been submitted in its current form from the beginning, I would likely have recommended rejection for publication in Nature. As Reviewer 2 also pointed out, the work is inevitably descriptive — which is acceptable — but what makes it stand out is the clear indication of the ecological roles of colony formation. Although the authors claim this is not a major point, I would argue that it is the major point. Without this emphasis, the significance of the manuscript is greatly diminished; it risks becoming merely another report describing aggregation behavior in a unicellular relative of animals and documenting its expression profiles, a line of work already pursued in other protists.

In this context, the second result is, as the authors themselves acknowledge, too weak to convince a broad Nature readership that *M. vibrans* undergoes sexual reproduction within colonies in response to specific bacterial species. First, the phenomenon has not been rigorously quantified; presenting a single image of double-stained cells is clearly insufficient. A statistical comparison between the colony-forming stage and the solitary stage is also missing, as the authors rightly note. Second, the possibility of cannibalism as an explanation for the double-stained cells cannot be excluded based on the current data. Third, direct evidence for the presence of gametes is entirely lacking, even though the authors place considerable emphasis on the upregulation of meiosis-related genes and hinge their argument for the occurrence of meiosis in *M. vibrans* on this point. Overall, in my view, more robust evidence that sexual reproduction preferentially occurs within colonies is indispensable for publication in a high-profile journal such as Nature.

3. Minor Rewording
The current description of the "Eph-like" receptor remains confusing. What exactly does "Eph-like" mean in this context? The manuscript gives the impression that two (or two sets of) orthologous genes have been identified in choanoflagellates and *M. vibrans*, and that these resemble animal Eph receptor genes (presumably in terms of domain architecture). However, in the referenced papers, I could not find solid evidence that choanoflagellates possess tyrosine kinases resembling the Eph receptor, nor do the authors provide evidence that the choanoflagellate genes are orthologous to the *M. vibrans* genes. These genes are therefore unlikely to represent true Eph receptors, and the authors should avoid implicating them (even indirectly) in animal morphogenesis.

Line 520: "a RBH" -> "an RBH"

Referee #2

(Remarks to the Author)
I think the authors have addressed all comments successfully. The improvements to the differential expression analysis, along with the new experiments involving the second Ministeria strain, xenic and axenic conditions, microscopy, and the identification of aggregation-inhibiting bacteria, have strengthened the manuscript overall.

Referee #3

(Remarks to the Author)
I co-reviewed this manuscript with one of the reviewers who provided the listed reports.

Referee #4

(Remarks to the Author)
The authors have addressed my concerns satisfactory. This paper is a very import contribution to the field and beyond.

(Remarks on code availability)

Ministeria vibrans functional annotation output files, alongside files used for analysis of RNA-sequencing data. RNA-seq data are accessible, so data provided are a usable resource for the community

Version 2:

Reviewer comments:

Referee #1

(Remarks to the Author)
The authors have provided sufficiently compelling evidence to convince readers of the occurrence of mating within aggregates of *M. vibrans*. The statistical demonstration of increased mating compared to the non-aggregative state, together with the video capturing the moment of cell fusion, is particularly persuasive. The revisions throughout the manuscript have further strengthened the central findings, including the proposed advantages of aggregation behavior in the unicellular ancestor of animals, rather than presenting merely a transcriptomic survey of another filasterean species.
I support publication of this manuscript in *Nature*, pending minor revisions to the Introduction section.

1. To improve accessibility for a broader readership, please clarify why other unicellular holozoans have not permitted detailed analyses of dynamic gene expression during the aggregation process, particularly around the sentence “Furthermore, gene expression has not been studied…”.
2. The Introduction is overly long and extends even into detailed results and conclusions, resembling an “extended summary”. Please revise it to be more concise.

Referee #2

(Remarks to the Author)
The authors have addressed my concerns and made a substantial effort to respond to the issue regarding mating. Although the data do not unequivocally demonstrate that sexual reproduction occurs in these cultures, the new experiments provide convincing evidence for fusion based on the fluorescent markers. I would recommend that the limitation of not being able to fully control or monitor the sexual life cycle of these organisms be explicitly acknowledged in the manuscript as a caveat. That said, I believe the authors have done as much as reasonably possible to address this issue experimentally.

Open Access This Peer Review File is licensed under a Creative Commons Attribution 4.0 International License, which permits use, sharing, adaptation, distribution and reproduction in any medium or format, as long as you give appropriate credit to the original author(s) and the source, provide a link to the Creative Commons license, and indicate if changes were made.
In cases where reviewers are anonymous, credit should be given to 'Anonymous Referee' and the source.
The images or other third party material in this Peer Review File are included in the article’s Creative Commons license, unless indicated otherwise in a credit line to the material. If material is not included in the article’s Creative Commons license and your intended use is not permitted by statutory regulation or exceeds the permitted use, you will need to obtain

permission directly from the copyright holder.
To view a copy of this license, visit https://creativecommons.org/licenses/by/4.0/

October 13, 2025

Dear Editor,

Enclosed please find our revised manuscript entitled, "A close unicellular relative reveals aggregative multicellularity was key to the evolution of animals" (manuscript 2025-05-12188) that we are resubmitting for consideration in *Nature*.

We were delighted to learn that all four Reviewers found the work interesting. As requested, we have revised the manuscript considering the Editor and Reviewers' comments. Our point-by-point responses to the queries and our changes to the text are provided in detail below:

<u>Reviewer 1:</u>

1. The Reviewer states: *'No rationale provided why monoxenic culture with the specific bacterial species T. lucentensis can induce the aggregation in Ministeria culture.*
Being free-living, Ministeria would hardly be in a monoxenic condition in nature. Such ability should immediately be removed during the course of evolution. Given that, the authors' claim that it was co-opted for constructing the primitive metazoan multicellular system does not make so much sense. Authors should thus conduct a couple of more experiments to understand why monoxenic culture of T. lucentensis can induce the aggregation and check if it is the only condition to do so. For example, they should check if other monoxenic culture with different bacterial species can form colonies, and how Ministeria would behave in dixenic (or even trixenic) culture with/without T. lucentensis. The authors should also eliminate the possibility that the original (multixenic) culture may contain a specific bacterial species inhibiting the colony formation.
The authors should also provide a reasonable explanation why the colony formation occurs only in a monoxenic culture, in other words, why Ministeria does not form colonies in a multixenic condition. The authors have depicted the efficient feeding and mating as selective advantages of colony formation, but it would be the same in a multixenic condition.'

We thank the reviewer for this thoughtful feedback, which has yielded fruitful results (new panels K-N in figure 6 & new sections in the results: "A second *M. vibrans* isolate aggregates in xenic culture with diverse bacteria" and "Aggregation is inhibited by two bacteria co-isolated with the Tong *M. vibrans* strain").

First, as suggested by the reviewer, we have investigated whether bacteria in the xenic culture inhibit aggregation (as opposed to *T. lucentensis* uniquely inducing aggregation). Indeed, this seems to be the case. We made dixenic and trixenic cultures by adding the other two most abundant bacteria from the xenic culture (*Vibrio plantisponsor* and *Pseudoalteromonas rhizospherae*) to the monoxenic *T. lucentensis* culture. We found that *P. rhizospherae* eliminated aggregation when it was present at < 1:10 ratio with *T. lucentensis* (its ratio in the xenic culture). *V. plantisponsor* also inhibited aggregation at its abundance in xenic culture, but to a lesser degree. These new data are presented in Fig. 6m & Fig. 6n.

Furthermore, this comment led us to re-investigate the original xenic culture of the second *M. vibrans* isolate ("L27", DSM 114853), which we previously discussed in the context of making its monoxenic *T.*

800 E. Kirkwood Ave., Room A563 **Department of Chemistry** 812/855-2068
Bloomington, IN 47405 jpgerdt@iu.edu

lucentensis culture (previous Fig. 3e). We had initially seen hints of minor aggregation with this xenic culture. We went back and carefully studied this xenic culture after removing a contaminating eukaryotic species (*Paraphysomonas imperforata*) and using our optimized growth medium. We found that under the optimized conditions this xenic culture could clearly aggregate, although the aggregate areas were smaller than those in monoxenic culture with *T. lucentensis* (new Fig. 6k & ED Fig. 10l). Nonetheless, obvious multicellular aggregates formed in this xenic culture, in which we found 14 different bacteria present by 16S amplicon sequencing (Data S13). Notably, none of these bacteria were closely related to *T. lucentensis*.

Overall, these new findings suggest that *M. vibrans* <u>can aggregate in more natural xenic environments</u>—as long as certain 'aggregation-inhibiting' bacteria are not present.

> 2. The Reviewer states: *'The evidence of sexual reproduction within the aggregation is too weak The authors describe the up-regulation of some meiosis-related genes as good evidence of sexual reproduction occurring in the aggregation. However, the two genes picked up as examples (GEX1 and HAP2) are best known to be involved in membrane fusion of plant gametes after pollination, not in meiosis per se. The authors should check the expression of other key meiosis-related genes such as those involved in double-stranded DNA break and synaptonemal complex assembly.*
> *If meiosis is vigorously occurring within the aggregation, it should contain many haploid cells, which the authors can confirm by simple DNA staining. The extremely stable Ministeria colony would allow them to do so.*
> *Lastly, the authors should explain why Ministeria do both grow and reproduce sexually at the same time. When a life can grow well in a certain condition, it would rather avoid undergoing sexual reproduction, which is costly. What is the ecological relevance of doing growth and sexual reproduction at the same time?'*

We agree with both Reviewers 1 & 2 that our evidence for meiosis and mating is not strong. Overall, we have downplayed this in our revised manuscript, since it is not the main point of the paper. We note a few specific responses/changes below:

Many other genes encoding proteins beyond GEX1 and HAP2 are included in the extended data and extensive supplementary information. We have now mentioned genes encoding proteins directly related to meiosis, and not only syngamy, that we found differentially expressed in the main text [lines 652-667 of marked manuscript], added more detail to Supplementary Discussion 3, added panel g to Extended Data Fig. 10, added Fig. S6, adjusted the phrasing in the figure and legends of Extended Data Fig. 10 and Fig. S5, and added additional differential expression information to Data S10 (formerly Data S11). We have also improved our phrasing throughout the manuscript and now refer to meiosis- and mating (specifically syngamy)- related genes separately throughout the text. Thank you for pointing out the poor wording. Specifically related to meiosis, we had previously found that genes encoding proteins involved in sister chromatid cohesion (SMC1, SMC3, and RAD21), introducing double-stranded breaks (MRE11 and RAD50), homologous recombination (RAD51, HOP2, and MND1), crossover resolution pathway I (MLH1), and gene conversion by mismatch repair (MSH2, MSH6, and PMS2) were differentially expressed (Extended Data Fig. 10 and Fig. S4). Given it could be that only a subpopulation of cells is undergoing meiosis and mating during aggregation we have newly investigated whether additional meiosis-related genes were differentially expressed with higher significance cutoffs (adjusted p-value < 0.00001 and < 0.001). We thereby found that additional genes encoding key proteins, for example, involved in synamptonemal complex assembly (PCH2, MSH4, and MSH5) and double-stranded breaks (SPO11), are also differentially expressed during the *M. vibrans* aggregation process. In fact, given the higher cutoffs, nearly all meiosis-related genes that we investigated and found in the *M. vibrans* genome were found to be differentially expressed (with the exception of HOP1, ZIP4, MER3, and DMC1). To better reflect these findings, we have updated Extended Data Fig. 10 by adding an additional panel (now

panel g) indicating which syngamy and meiosis-related genes investigated are differentially expressed, which are specific to these processes, and the significance cutoff used to detect them. In addition, we have added Fig. S6 which shows the expression profiles for each of these genes.

Even so, our evidence for meiosis and mating is still weak without any phenotypic validation (e.g., quantification of DNA content—which is challenging due to the pervasive presence of bacterial DNA in the culture and inside *Ministeria* food vacuoles; and fusion of cells—which is difficult to confidently observe within aggregates). We did perform an initial test to see if two stained populations of *M. vibrans* form double-stained cells in aggregation-inducing conditions. If so, cells may be fusing. Indeed, we found double-stained cells (new Fig. 6i & ED Fig. 10i–k). We emphasize the limitations of this in the text, noting this is an 'initial test', that 'we did not quantify these events relative to non-aggregation conditions', and that 'more extensive investigation is necessary to test if meiosis and mating are truly occurring in aggregates'.

To the second point, we appreciate the concern that mating is generally considered a 'stress response' in unicellular organisms. This should be at odds with our observed robust cell division in the aggregation-inducing conditions. We added further discussion of this point [lines 669-676 of marked manuscript], raising this concern along with 2 counter-points: (1) our RNAseq results were a 'bulk' measurement of the entire population. The putative meiosis/mating cells may be a separate subset of cells than those that are replicating quickly. If the meiosis/mating subset highly upregulate those genes, they would still appear as upregulated at the population level, (2) the hypothesis that mating is counter to robust cell growth has not been comprehensively tested across all organisms. It is possible that *Ministeria* actually does mate under good growth conditions. We note that the choanoflagellate *S. rosetta* mates in the presence of *Vibrio fischeri*, a condition that is still compatible with growth.

Ultimately, we have toned down our emphasis of this observation, making explicit discussion of the caveats in the results section [lines 666-667 of marked manuscript] and making the uncertain nature more clear in the discussion [lines 823-828 of marked manuscript]. We also removed the statement from the abstract, since it is less certain and not the main outcome of the work.

3. The Reviewer states: *'The followings are some minor problems that the author may want to fix.'*

Line 274: In the legend of figure 5a, the colour code must be clearly described (being the same as in figure 4c).

We have now explicitly stated that the DEG clusters correspond to those outlined in Fig. 4c and that the colour legend can be found in panel c of Fig. 5.

Line 283-: In the legend of figure 5c, the commas and semicolons are in a reversed order. For instance, it has to be like "Spectrin, SPT; fascin, FSCN; …".

We have adjusted as requested both here and throughout the manuscript.

Line 336: With the reference 57, which lacks the comprehensive phylogenetic analysis including other tyrosine kinases, the receptor tyrosine kinase gene that the authors found upregulated would not confidently be identified as an eph receptor homologue. Please look more in detail and rephrase.

We have rephrased and added a caveat as requested [lines 519–524 of marked manuscript], alongside now referring to the gene as an "eph-like receptor tyrosine kinase".

Line 470-472: A reference should be included.

We have added references for these statements as requested.

Reviewer 2:

1. The Reviewer states: *'The authors investigate whether disaggregated cells grow less effectively than those remaining in aggregates, and they indeed show that aggregated cells exhibit better growth. However, could the authors comment on the possibility that the disaggregation protocol itself may have interfered with cell division?'*

Indeed, it is plausible that pipetting up/down could damage cells to lower their cell count, or have some other effect on cellular replication. We have more explicitly mentioned this possibility [lines 597–599 of marked manuscript]

We have also added a second experiment that avoids pipetting up/down and supports the same conclusion. We have grown the culture on tissue culture-treated surfaces that prevent aggregation and compared replication to cells grown in the normal condition on low-attachment surfaces that allow aggregation. Again, in this case, the non-aggregated cells (on higher attachment surfaces) replicated worse (new Extended Data Fig. 10b–c). This new experiment carries its own caveat that the *M. vibrans* cells migrate slower on the tissue culture-treated surface, which may decrease their ability to capture bacteria, regardless of their ability to aggregate. We have explicitly noted this caveat in the manuscript [lines 612–614 of marked manuscript]. Therefore, the experiments each have caveats that decrease the certainty of our conclusion that aggregation promotes growth. Since these caveats are independent of each other, both would need to be true to discard the hypothesis. Therefore, we believe the most likely explanation for our results is that aggregation promotes growth of *M. vibrans*.

2. The Reviewer states: *'The authors show that Hap2 and Gex, genes known to be involved in mating, are upregulated during aggregation. There seems to be a misunderstanding in the conclusions because these two proteins function in cell and nuclear fusion, respectively and not directly in meiosis. Given that Hap2 is a well-established fusogen, it would have been important to provide evidence of cell fusion in the aggregates, which would support the claim that sexual reproduction plays a role in driving aggregative behavior.'*

Thank you for pointing out our poor phrasing, we have now adjusted our wording throughout the manuscript to directly refer to meiosis and mating (specifically genes encoding proteins involved in syngamy for the later) separately. We had also found that a large proportion of genes encoding proteins related to meiosis in the *M. vibrans* genome were differentially expressed during aggregation, including meiosis-specific markers PCH2, MSH4, MSH5, SPO11, HOP2, and MND1. Some of these became evident after loosening the significance cutoff, which we explained in the text. We include these additional genes in the main text, have expanded the Supplementary Discussion 3, added a panel to Extended Data Fig. 10 (panel g), and added Fig. S6. Please see the response to reviewer 1 for more details on these additions. Regardless, without experimental evidence of cell fusion, as suggested, our evidence for meiosis and mating occurring during aggregation is not strong. As this is not a major finding of our work, we have now downplayed these results in our revised manuscript.

Even so, our evidence for meiosis and mating is still weak without any phenotypic validation (e.g., quantification of DNA content—which is challenging due to the pervasive presence of bacterial DNA in the culture and inside *Ministeria* food vacuoles; and fusion of cells—which is difficult to confidently observe within aggregates). We did perform an initial test to see if two stained populations of *M. vibrans* form double-stained cells in aggregation-inducing conditions. If so, cells may be fusing. Indeed, we found double-stained cells (new Fig. 6i & ED Fig. 10i–k). We emphasize the limitations of this in the text, noting

this is an 'initial test', that 'we did not quantify these events relative to non-aggregation conditions', and that 'more extensive investigation is necessary to test if meiosis and mating are truly occurring in aggregates'.

Ultimately, as this is not a major finding of our work, we have now downplayed these results in our revised manuscript.

3. The Reviewer states: *'It would strengthen the manuscript if the authors highlighted more clearly the divergence times from the last common ancestor of Filozoa, Capsaspora, and choanoflagellates.'*

Although these timelines are not firm, an approximation is ~1000 million years ago (MYA) for the last common ancestor (LCA) of Filozoa, ~900 MYA for the LCA of Choanozoa, and ~750 MYA for the LCA of Metazoa (doi: 10.1073/pnas.1110633108). We have added these numbers to Fig. 1a.

4. The Reviewer states: *'It would be useful to clarify the genetic diversity of the original M. vibrans strain used in the study. Are the aggregating cells genetically distinct individuals, or is the culture largely clonal? This distinction affects the interpretation of the aggregation as a true social/multicellular behavior where competition and cheating could happen. Testing aggregation of mixed-strain cultures would help clarify this point (eg. M. vibrans ATCC and M. vibrans DSM 114853 both used in this study).'*

Thank you for the suggestion. We believe the culture is largely clonal. In any case, we have now performed a mixed-strain aggregation experiment (new Fig. 6l). The two strains co-aggregate very well. This suggests that they have the same mechanism of aggregation and that there is not a strong mechanism of discriminating between clonal mates and more distantly related kin.

5. The Reviewer states: *'The ecological relevance of the observed aggregative phenotype could be discussed in more detail. Given that aggregation is only induced in monoxenic conditions, the relationship between this phenotype and the natural ecological niche of M. vibrans (which is likely diverse, given the also diverse bacterial community present in the original culture, lines 110-113) deserves further exploration.'*

As discussed above in response to Reviewer 1, our new experiments suggest that xenic *M. vibrans* cultures can aggregate (new Fig. 6k & ED Fig. 10l), as long as an 'aggregation inhibiting bacterium' is not present at high levels (new Fig. 6m–n). Therefore, many natural niches likely promote aggregation.

6. The Reviewer states: *'The authors elegantly show that initial aggregate formation is independent of division (aphidicolin experiment, Fig.3). However, the reduced aggregate area in the aphidicolin condition compared to the control suggests that subsequent clonal division, coenocyte formation, or even mating may contribute to aggregate growth and maintenance. This possibility, also acknowledged by the authors, could be further explored using time-lapse microscopy and single-cell tracking to clarify the cellular dynamics and composition of long-term aggregates.'*

We agree that it would be fascinating to track cell movement and replication within aggregates. However, in our opinion, this is a large undertaking beyond the scope of this manuscript and deserving of its own focus in subsequent work.

7. The Reviewer states: *'The role of Thalassospira bacteria in the aggregate formation remains somewhat ambiguous. Is aggregate formation triggered by direct physical contact, feeding or secreted factors? While the authors briefly acknowledge this uncertainty, the manuscript would benefit from*

experiments testing whether bacterial conditioned ASW alone can induce aggregation, or if live bacterial presence is required.'

As discussed above in point 5 and in response to Reviewer 1, our new experiments suggest that xenic *M. vibrans* cultures can aggregate (new Fig. 6k & ED Fig. 10l), as long as an 'aggregation inhibiting bacterium' is not present at high levels (new Fig. 6m–n). In ongoing work, we are trying to elucidate the specific factors that inhibit aggregation, but these experiments are beyond the scope of this work.

8. The Reviewer states: *'The authors mention the formation of a "putative bacterial biofilm" within the aggregate (Fig. 6). This should be better characterized using microscopy (e.g., staining for bacterial cells, extracellular matrix, TEM) and bacterial cell tracking to determine whether the bacteria actively proliferate within the aggregates. Can the authors be sure that the cells accidentally are not being 'stuck together' because of the bacterial biofim?'*

We thank the reviewer for the suggestion. We have now fluorescently labeled the *T. lucentensis* bacteria using the selective stain 5-Cyano-2,3-ditolyl tetrazolium chloride (CTC) and presented new confocal fluorescence microscopy images that show bacteria within the intracellular spaces of the aggregate (new Fig. 6f).

However, tracking individual bacterial cells within the aggregate is too technically challenging at this point. Therefore we cannot be sure that the bacteria are dividing within aggregates. We make that clear in the text [lines 835–836 of marked manuscript].

To the third point, our SEM images (Extended Data Fig. 4) are highly suggestive that *M. vibrans* cells aggregate by retracting intertwined filopodia (which also matches our previous observations from live-cell imaging in *C. owczarzaki*). In the *M. vibrans* SEM images, there is no obvious bacterial biofilm between or around the filopodia. Therefore we are fairly confident that the aggregation is not just biofilm crudely 'gluing' cells into a clump.

9. The Reviewer states: *'Overall, the figures are clear and well-presented. However, a few minor improvements are suggested:*

- Fig. 3d and e: add labels on the plots so the reader can directly understand what they refer to without having to go through the legend. For both these plots a xenic control should also be provided

We have added labels as requested and added xenic data to Fig. 3d and the former Fig. 3e (now Fig. 6k). We especially thank the reviewer for this suggestion that led us to reinvestigate the xenic *M. vibrans* L27 culture and see that it actually did aggregate. This observation helped us respond to other reviewer comments and re-order the manuscript for clarity.

- Fig. 4b: color difference between the time points 12h and 18h is very difficult to see, especially in printed copy. Please change the color scheme or label the points in the plot

We thank the Reviewer for this feedback and have added labels to panel 4b

- Fig. 5a, Extended Data Figure 10 c – genes not belonging to a specific DEG cluster should also be included in these plots'

We have now adjusted Fig. 5a, Extended Data Fig. 9a, and Extended Data Fig. 10c (now panel e) so that all differentially expressed genes not assigned to a specific DEG cluster are also indicated. For

Extended Data Fig. 9a, we have also now excluded groups represented by only one DEG. For the revised Extended Data Fig. 10, please see the response to Reviewer 1.

Reviewer 4 (no unique comments provided by Reviewer 3):

1. The Reviewer states: *'The authors mention in the abstract: It is unclear whether aggregation in these species is derived or ancestral, and its relevance for animal origins remains unknown. I wished this would have been discussed more clearly in the manuscript. Is it derived or ancestral?'*

We thank the Reviewer for this feedback and have added some more discussion of this point in the first and third paragraphs of the discussion section.

2. The Reviewer states: *'This touches on the point raised above: The title of the paper is: "A close unicellular relative reveals aggregative multicellularity was key to the evolution of animals". Was it really key to the evolution of animals? There are other explanations, and the authors should discuss these in the paper.'*

We thank the Reviewer for this feedback and have added two new paragraphs to the discussion section exploring alternative explanations.

3. The Reviewer states: *'The key findings predominantly rely on transcriptomics. Would the authors have the chance/opportunity to use small molecule inhibitors or maybe even Crispr/Cas9 to provide evidence of direct participation in the aggregation of at least one upregulated protein/factor. If this is not possible (due to Ministeria not yet being fully developed as model organism, although inhibitor test should be feasible) this should be clearly mentioned in the manuscript.'*

Indeed, we thank the reviewer for raising this important limitation of our study and have added more text at the end of the transcriptomics results section emphasizing the need for functional validation of the discovered pathways [lines 559–564 of marked manuscript]. *M. vibrans* has no genetic tools yet. We are working on developing that, but we anticipate it taking a long time. We have likewise begun exploring different chemical inhibitors. However, the results are complicated by both (a) the lack of validation of many inhibitors outside of mammals and (b) the off-target effects of many inhibitors that could cause stresses that prevent aggregation. A third possibility is to perform genetic knockouts of the same pathways in *C. owczarzaki*, which already has validated genetic tools. We are pursuing all three options for subsequent work.

4. The Reviewer states: *'Have the authors identified new "multicellularity genes involved in animal cell adhesion, signaling, and transcriptional regulation" which previously were not known in filastereans? If so, these could be highlighted more prominently.'*

We only identified a few cases of multicellularity gene homologs previously unknown in filastereans or with limited prior information in filastereans, hence we have not highlighted this. But the few potential cases that we did find are already mentioned in the main text and accompanying Supplementary Discussion 2. Specifically, genes encoding the canonical C-type lectin protein domain (PF00059), which as far as we are aware hasn't previously been reported in Filasterea, and genes encoding the hint/hog protein domain (PF01079), which had previously been found in an older *M. vibrans* cDNA library (https://doi.org/10.1371/journal.pone.0002098) but was not captured in the more recent transcriptome, and as far as we are aware has not been reported in other filastereans. In addition, we found that the *M. vibrans* genome encodes an expanded repertoire of putative cadherins (PF00028) relative to reports from other filastereans (eight vs. the one reported in *C. owczarzaki*). We have now better highlighted the

additional information about the multicellularity genes identified in Supplementary Discussion 2. We have also added colour-coding to Data S9 (formerly Data S10) so that it is easier to see which genes we did and did not find putative homologs of.

5. The Reviewer states: *'I was not able to review the code (https://figshare.com/articles/dataset/dx_doi_org_10.6084/m9.figshare.29027924), as "page was not found"'*

Thank you for pointing out the difficulty in accessing the data. We have updated the DOI "10.6084/m9.figshare.29027924" in the manuscript to a direct link "https://doi.org/10.6084/m9.figshare.29027924" to facilitate easier access.

We also wanted to point out that we have updated Data S4 and the Data Availability statement to include the GenBank accession for the assembled *Thalassospira lucentensis* Mv1 genome and the 16S rDNA amplicon sequence reads for the *Ministeria vibrans* DSM_114853 xenic culture.

We have included a marked copy of the revised manuscript that shows changes made on revision clearly highlighted, as requested. We believe that these revisions have further strengthened the manuscript, and we are grateful to the Reviewers for their comments. We thank you in advance for your consideration of this revised manuscript.

Sincerely,

Joseph P. Gerdt, Ph.D.
Assistant Professor of Chemistry
Adjunct Professor of Biology

Iñaki Ruiz-Trillo, Ph.D.
ICREA Research Professor
Institute of Evolutionary Biology (CSIC-UPF)

April 6, 2026

Dear Editor,

Enclosed please find our revised manuscript entitled, "*A close unicellular relative reveals aggregative multicellularity was key to the evolution of animals*" (manuscript 2025-05-12188A) that we are resubmitting for consideration in *Nature*.

We are grateful for the thoughtful review of our manuscript by yourself and the reviewers. In this revised version, we have significantly strengthened the manuscript by providing three new pieces of data that are supportive of mating occurring in the aggregative stage.

While demonstrating mating molecularly remains a technical frontier in our non-model system (no genetic tools are available), we believe our new data provide supportive evidence of this behaviour. These include: (1) mixing assays of single-stained populations demonstrate a significant increase in the percentage of dual-stained cells under aggregation compared to non-aggregation conditions, (2) sub-cellular imaging reveals that the two stains of dual-stained cells are both distributed across the cells and not localized to food vacuoles, suggesting true cell integration rather than prey ingestion, and (3) a direct visualization (video) of a cell fusion event occurring between two cells in aggregation conditions.

Our point-by-point responses to the queries and our changes to the text are provided in detail below:

Reviewer 1:

1. The Reviewer states: *'1. Relevance of the Monoxeny of Colony-Inducing Bacteria. The authors have provided satisfactory evidence that M. vibrans is capable of aggregating in more natural, xenic environments. In addition, their new results using a second M. vibrans strain, together with the detailed bacterial profiling of the aggregation-inducing and aggregation-inhibiting species, further strengthen one of their key conclusions. These findings also lay a solid foundation for more detailed future investigations into the ecological relevance of M. vibrans aggregation.'*

We thank the reviewer for their positive feedback and time considering the new draft.

2. The Reviewer states: *'2. Evidence of Sexual Reproduction. The authors have provided two new results: (1) a substantial number of meiosis-related genes are upregulated in the M. vibrans colony, in addition to the previously identified genes that are primarily involved in membrane fusion between gametes; and (2) a subpopulation of double-stained cells is observed in the colony after mixing two differently labeled cell populations.*

The first result strongly suggests the presence of gamete production within the colony, which is highly intriguing. In light of this, the authors' revision that downplays the role of colony formation is somewhat disappointing. To be frank, if the manuscript had been submitted in its current form from the beginning, I would likely have recommended rejection for publication in Nature. As Reviewer 2 also pointed out, the work is inevitably descriptive — which is acceptable — but what makes it stand out is the clear indication of the ecological roles of colony formation. Although the authors claim this is not a major

800 E. Kirkwood Ave., Room A563
Bloomington, IN 47405
Department of Chemistry
812/855-2068
jpgerdt@iu.edu

point, I would argue that it is the major point. Without this emphasis, the significance of the manuscript is greatly diminished; it risks becoming merely another report describing aggregation behavior in a unicellular relative of animals and documenting its expression profiles, a line of work already pursued in other protists.'

We appreciate the reviewer's push to further highlight the potential gamete formation in aggregates. We certainly did not intend to downplay the significance of the sexual signatures; rather, our decision to focus on the genomics of aggregation was a deliberate choice to address what we consider the most foundational, yet unresolved, question in the field: the viability of the aggregative path to animal multicellularity.

While we agree with the reviewer that the potential for sexual reproduction within *M. vibrans* colonies is a fascinating discovery—one that we have now bolstered with three new lines of empirical evidence—we maintain that the primary point of this work is the dynamic deployment of the metazoan 'multicellular toolkit' during the aggregation process. Indeed, the hypothesis that the metazoan 'multicellular genetic toolkit' was first assembled and deployed within an aggregative context remains largely unexplored and lacks the formal genomic evidence we provide here.

Indeed, the reason we chose *M. vibrans* was specifically to test this: unlike the clonal models (e.g., choanoflagellates), *M. vibrans* offers a unique window into how animal-like genes (for adhesion, signaling, and now, meiosis) are recruited during cell aggregation. By proving that this toolkit is active and regulated in these aggregates, we are offering a fundamental rethinking of the 'clonal-only' dogma of animal origins.

However, we fully respect and appreciate the reviewer's perspective that the ecological/sexual angle is also interesting for a broad readership. Therefore, in this revision, we have:

-Integrated the sexual aspect more deeply into our manuscript, and

-Added new experimental data (cell mixing assays and live imaging) that provide additional evidence for this potential behavior.

We believe that by challenging the clonal origin theory while also providing exciting evidence for a potential ecological role, as the reviewer highlighted, we are indeed enhancing the manuscript's impact for *Nature*.

3. The Reviewer states: *'In this context, the second result is, as the authors themselves acknowledge, too weak to convince a broad Nature readership that M. vibrans undergoes sexual reproduction within colonies in response to specific bacterial species. First, the phenomenon has not been rigorously quantified; presenting a single image of double-stained cells is clearly insufficient. A statistical comparison between the colony-forming stage and the solitary stage is also missing, as the authors rightly note.'*

We agree and have now tested this phenomenon quantitatively with multiple replicates under aggregating and non-aggregating conditions. Approximately a 5x greater percentage of cells had both stains in the aggregation condition compared to the non-aggregation condition. See new Figure 6j.

4. The Reviewer states: *'Second, the possibility of cannibalism as an explanation for the double-stained cells cannot be excluded based on the current data.'*

We agree that distinguishing between cell fusion and phagocytosis is critical. We note that we have never directly observed cannibalism in *M. vibrans*, nor has it been reported in this organism. Nonetheless, to

address this concern, we performed high-resolution subcellular imaging to track the fate of the internalized fluorophores.

In a cannibalistic/phagocytic scenario, the 'prey' fluorophore would be, theoretically, sequestered within acidified, membrane-bound food vacuoles (phagosomes), appearing as discrete, high-intensity puncta distinct from the 'predator's' cytoplasm and plasma membrane. Instead, our imaging (see Video S4) reveals that in 100% of the dual-stained cells analyzed (n=16), both fluorophores are broadly distributed throughout the cell. Nonetheless, we cannot completely rule out the possibility that the stain gets re-distributed from the 'food' throughout the cell within the timeframe of the experiment. We have added a new video (Video S4; representative of 16 dual-stained cells) and the text below:

> "We considered the possibility that the double-staining occurred due to cannibalism instead of mating. In that case, we might expect one of the dyes to be localized to a food vacuole. However, we constructed 3D image stacks of several of the double-stained cells and found both dyes spread through each cell (Video S4 is representative of 16 cells imaged). Although we cannot rule out the possibility that dyes distribute from the food vacuole over the course of the experiment, our observation of widespread staining supports the hypothesis that plasma membranes and cytosols are fusing."

Moreover, with extensive time-lapse imaging, we were also able to capture a single cell-fusion event occurring in our aggregation conditions. To our eyes, it resembles a symmetric cell fusion event more than one cell phagocytosing another. See new Video S5 and Figure 6k.

5. The Reviewer states: *'Third, direct evidence for the presence of gametes is entirely lacking, even though the authors place considerable emphasis on the upregulation of meiosis-related genes and hinge their argument for the occurrence of meiosis in M. vibrans on this point.'*

The reviewer correctly notes the lack of ploidy data. We attempted to quantify DNA content via flow cytometry; however, the persistent presence of bacterial DNA (both in food vacuoles and extracellularly) creates a signal-to-noise ratio that precludes the identification of discrete ploidy peaks. This is a known hurdle in holozoan research, often requiring years of species-specific protocol development (e.g., nuclei isolation). We indeed attempted multiple methods but were, unfortunately, unsuccessful in counting nuclei.

Rather than 'hinging' our argument solely on gene expression, and thanks to the reviewer, we now present it as a concordant dataset: the simultaneous upregulation of meiotic markers, the observation of cell fusion (Video S5, Fig. 6k), and the cytoplasmic mixing (Video S4, Fig. 6i) collectively point to meiosis happening within aggregates. We have revised the text to frame this as a 'compelling signature of mating' rather than a 'proven meiotic event,' acknowledging that the formal characterization of gametes remains a major future objective for the field.

6. The Reviewer states: *'Overall, in my view, more robust evidence that sexual reproduction preferentially occurs within colonies is indispensable for publication in a high-profile journal such as Nature.'*

We believe that by providing three independent, reinforcing lines of empirical data—transcriptomic, population-level mixing, and live-cell imaging—we have moved this finding well beyond a plausible hypothesis. We thank the reviewer for pushing us to extend the evidence to the amount that we can within a reasonable timeframe for publication.

7. The Reviewer states: *'3. Minor Rewording. The current description of the "Eph-like" receptor remains confusing. What exactly does "Eph-like" mean in this context? The manuscript gives the impression that two (or two sets of) orthologous genes have been identified in choanoflagellates and M. vibrans, and that these resemble animal Eph receptor genes (presumably in terms of domain architecture). However, in the referenced papers, I could not find solid evidence that choanoflagellates possess tyrosine kinases resembling the Eph receptor, nor do the authors provide evidence that the choanoflagellate genes are orthologous to the M. vibrans genes. These genes are therefore unlikely to represent true Eph receptors, and the authors should avoid implicating them (even indirectly) in animal morphogenesis.'*

We thank the reviewer for reiterating this important point. We agree that our original wording could be interpreted as implying a formal orthologous relationship, which would require more extensive evolutionary analysis than is within the scope of this study. Consistent with previous work indicating that Eph receptors are animal-specific, we have removed all references to "Eph-like" receptors and now classify this gene more conservatively within the broader category of protein-tyrosine kinases.

In response to this comment, we also undertook a comprehensive reassessment of all gene annotations. Initial annotations were based on domain composition, gene family and ortholog assignments, and reciprocal best hits to human homologs. We have now refined these annotations by additionally verifying whether genes annotated as specific gene orthologs or homologs correspond to a previously described ortholog or homolog in the well-studied model filasterean *C. owczarzaki*. Where this criterion was not met, annotations were re-assessed and either removed or in most cases reassigned to more general categories. In several cases, we have now added "domain-containing" designations and included all genes encoding the relevant domains, rather than only including genes that were reciprocal best hits. We also reviewed all acronyms for clarity and consistency.

Specific changes include

- the reannotation of formin-like protein as a diaphanous-related formin (DRF)
- removal of GRB2 due to insufficient support with only protein domains and without additional evolutionary analyses, and
- reassignment of protein tyrosine kinases not previously described in *M. vibrans* (10.1126/scisignal.2002733) to the general category. We only retained protein tyrosine kinases in that category where we found catalytic motifs or similarity to those previously described in *M. vibrans*.
- In addition, the gene we previously annotated as paxillin was removed, as it more closely resembles a different but related LIM domain–containing protein. Despite paxillin being previously described in *M. vibrans* (10.1016/j.cub.2020.08.061), we could not detect a clear paxillin ortholog.
- We have also now excluded two talin-like sequences with partial domain structure, and retained only a high-confidence talin homolog with strong similarity to *C. owczarzaki* talin.
- ERM was relabelled as ERM–merlin to better reflect the broader gene family, LIM-HD relabelled as HD due to insufficient support without additional analyses, and SOX as SOX-like due to the absence of the diagnostic Tyr72 residue (instead encoding Phe72, consistent with some SOX-like genes described in filozoans; 10.1038/s41467-024-54152-x).
- Protein kinase C (PKC) annotations were revised, with only atypical PKC now shown in Figure 5 and PKCs moved to Extended Data Figure 9.
- We expanded hint and patched annotations to include all genes encoding these domains, and not only reciprocal best hits to human homologs, and now refer to these as hint domain–containing and patched domain–containing proteins.
- Genes annotated as NOTCH were similarly revised to "Notch domain–containing proteins."

- The IP_3R/RyR category was also expanded to include all such domain containing proteins, and homologs previously described in *M. vibrans* (10.1093/molbev/msv098) are now also explicitly annotated.
- These changes, alongside additional minor rewording of additional gene categories and abbreviations, required updates to the plots and heatmaps in Figure 5, Extended Data Fig. 9, Figure S3, and associated repository files.

Given that we were implementing these changes, we made further improvements to clarity throughout the manuscript. Genes associated with small GTPase signalling were expanded to include RasGEFs and RasGAPs (previously only RhoGEFs and RhoGAPs were included, even though both Ras and Rho GTPases had been included). Axis and legend labels were improved and made more consistent, and category groupings in Extended Data Figure 9 were also refined with genes from the same signalling pathway grouped together. To improve transparency, we have also added a "Note" column to Data S9 summarizing the evidence supporting each gene annotation, including domain presence and structure, gene family and ortholog assignments, corresponding genes from *C. owczarzaki*, and references to previously characterized *M. vibrans* genes. Rows corresponding to genes not detected in *M. vibrans* were also removed to improve clarity. We have also revised phrasing throughout the manuscript to reflect the uncertainty inherent in gene age inference and the potential for changes to estimates with expanded taxon sampling. Finally, we added additional Pfam domains to the Supplementary Discussion.

These refinements primarily affect annotation stringency and terminology and <u>do not alter our conclusions</u>. The results remain robust, with 92%, 77%, and 55% of differentially expressed genes associated with cell adhesion, cell signalling, and transcriptional regulation assigned to net upregulated clusters (previously 89%, 76%, and 55%). We thank the reviewer for prompting this reassessment, which has strengthened the rigor and clarity of our analysis.

8. The Reviewer states: *'Line 520: "a RBH" -> "an RBH"'*

We thank the reviewer for pointing this out. This sentence has been removed to address a previous comment.

<u>Reviewer 2:</u>

1. The Reviewer states: *'I think the authors have addressed all comments successfully. The improvements to the differential expression analysis, along with the new experiments involving the second Ministeria strain, xenic and axenic conditions, microscopy, and the identification of aggregation-inhibiting bacteria, have strengthened the manuscript overall.'*

We thank the reviewer for their positive feedback and time considering the new draft.

<u>Reviewer 4 (no unique comments provided by Reviewer 3):</u>

1. The Reviewer states: *'The authors have addressed my concerns satisfactory. This paper is a very import contribution to the field and beyond.'*

We thank the reviewer for their positive feedback and time considering the new draft.

2. The Reviewer states: *'Ministeria vibrans functional annotation output files, alongside files used for analysis of RNA-sequencing data. RNA-seq data are accessible, so data provided are a usable resource for the community'*

We thank the reviewer for their time checking the data accessibility.

We have included a marked copy of the revised manuscript that shows changes made on revision clearly highlighted, as requested. We believe that these revisions have further strengthened the manuscript, and we are grateful to the Reviewers for their comments. We thank you in advance for your consideration of this revised manuscript.

Sincerely,

Joseph P. Gerdt, Ph.D.
Assistant Professor of Chemistry
Adjunct Professor of Biology

Iñaki Ruiz-Trillo, Ph.D.
ICREA Research Professor
Institute of Evolutionary Biology (CSIC-UPF)
